# Efficacy and safety of antibody–drug conjugates in the treatment of urothelial cell carcinoma: a systematic review and meta-analysis of prospective clinical trials

**DOI:** 10.3389/fphar.2024.1377924

**Published:** 2024-06-12

**Authors:** Jun-Wei Ren, Ze-Yu Chen, Yun-Jin Bai, Ping Han

**Affiliations:** ^1^ Department of Urology, West China Hospital, Sichuan University, Chengdu, China; ^2^ Institute of Urology, West China Hospital, Sichuan University, Chengdu, China

**Keywords:** antibody–drug conjugate, ADC, transitional cell carcinoma, urothelial cell carcinoma, single-arm meta-analysis

## Abstract

**Introduction:** Urothelial carcinoma (UC) is a refractory disease for which achieving satisfactory outcomes remains challenging with current surgical interventions. Antibody–drug conjugates (ADCs) are a novel class of targeted therapeutics that have demonstrated encouraging results for UC. Although there is a limited number of high-quality randomized control trials (RCTs) examining the use of ADCs in patients with UC, some prospective non-randomized studies of interventions (NRSIs) provide valuable insights and pertinent information. We aim to assess the efficacy and safety of ADCs in patients with UC, particularly those with locally advanced and metastatic diseases.

**Methods:** A systematic search was conducted across PubMed, Embase, the Cochrane Library, and Web of Science databases to identify pertinent studies. Outcomes, such as the overall response rate (ORR), disease control rate (DCR), progression-free survival (PFS), overall survival (OS), adverse events (AEs), and treatment-related adverse events (TRAEs), were extracted for further analyses.

**Results:** Twelve studies involving 1,311 patients were included in this meta-analysis. In terms of tumor responses, the pooled ORR and DCR were 40% and 74%, respectively. Regarding survival analysis, the pooled median PFS and OS were 5.66 months and 12.63 months, respectively. The pooled 6-month PFS and OS were 47% and 80%, while the pooled 1-year PFS and OS were 22% and 55%, respectively. The most common TRAEs of the ADCs were alopecia (all grades: 45%, grades ≥ III: 0%), decreased appetite (all grades: 34%, grades ≥ III: 3%), dysgeusia (all grades: 40%, grades ≥ III: 0%), fatigue (all grades: 39%, grades ≥ III: 5%), nausea (all grades: 45%, grades ≥ III: 2%), peripheral sensory neuropathy (all grades: 37%, grades ≥ III: 2%), and pruritus (all grades: 32%, grades ≥ III: 1%).

**Conclusion:** The meta-analysis in this study demonstrates that ADCs have promising efficacies and safety for patients with advanced or metastatic UC.

**Systematic review registration:** https://www.crd.york.ac.uk/prospero/, identifier: CRD42023460232

## 1 Introduction

Urothelial carcinoma (UC) is the sixth most common tumor reported in developed countries (Siegel et al., 2021). Bladder cancer (BC) is the most common malignant neoplasm of the urothelial tract, and its mortality rate is approximately four times higher than those among women globally (Sung et al., 2021). Upper-tract UC is relatively infrequent, accounting for only 5%–10% of all UC cases, and the estimated annual incidence rate in western countries is nearly 2 cases per 100,000 residents (Siegel et al., 2021). In clinical practice, surgical tumor resections alone often fail to achieve satisfactory treatment outcomes, necessitating additional treatment modalities. Antibody–drug conjugates (ADCs) have emerged as promising therapeutics in this field and have captured the attention of researchers. ADCs generally integrate the benefits of monoclonal antibodies for precise targeting as well as payloads for efficient killing and represent biotechnological drugs combining humanized or human monoclonal antibodies, a linker, and a payload (Tarantino et al., 2022). Common ADC payloads include microtubule inhibitors and DNA-damaging agents, with the microtubule inhibitors constituting over half of the ADC drugs in clinical development (Wang et al., 2023). Despite reported clinical trials on over 100 ADCs, only 14 have received the approval of the United States Food and Drug Administration (FDA) for clinical use (Samantasinghar et al., 2023). However, the design of ideal ADCs remains challenging, necessitating continuous efforts. Non-randomized studies on interventions (NRSIs) constitute a crucial evidence base in various fields and offer high precision through extensive datasets. Owing to practical or ethical constraints, randomized control trials (RCTs) may not always be feasible; therefore, NRSIs are important for evaluating the effectiveness of interventions in these domains (Igelström et al., 2021). Herein, we integrate prospective clinical trial data, including RCTs and NRSIs, with the aim of quantitatively integrating the findings and enhancing both the efficacy and safety evaluations by consolidating data from these studies for the overall response rate (ORR), disease control rate (DCR), progression-free survival (PFS), overall survival (OS), and adverse events (AEs), specifically the treatment-related adverse events (TRAEs).

## 2 Materials and methods

This study was registered in the International Prospective Register of Systematic Reviews (number: CRD42023460232).

### 2.1 Search strategy

This work is reported in line with the preferred reporting items for systematic reviews and meta-analyses (PRISMA) 2020 statement and the guidelines of a measurement tool to assess systematic reviews (AMSTAR) (Shea et al., 2017; Page et al., 2021). We systematically searched the PubMed, Embase, Cochrane Library, and Web of Science databases for relevant studies. The date of the last search was 9 September 2023; MeSH terms and free-text keywords were employed in the search process, as demonstrated in the Supplementary Table S1.

**FIGURE 1:**
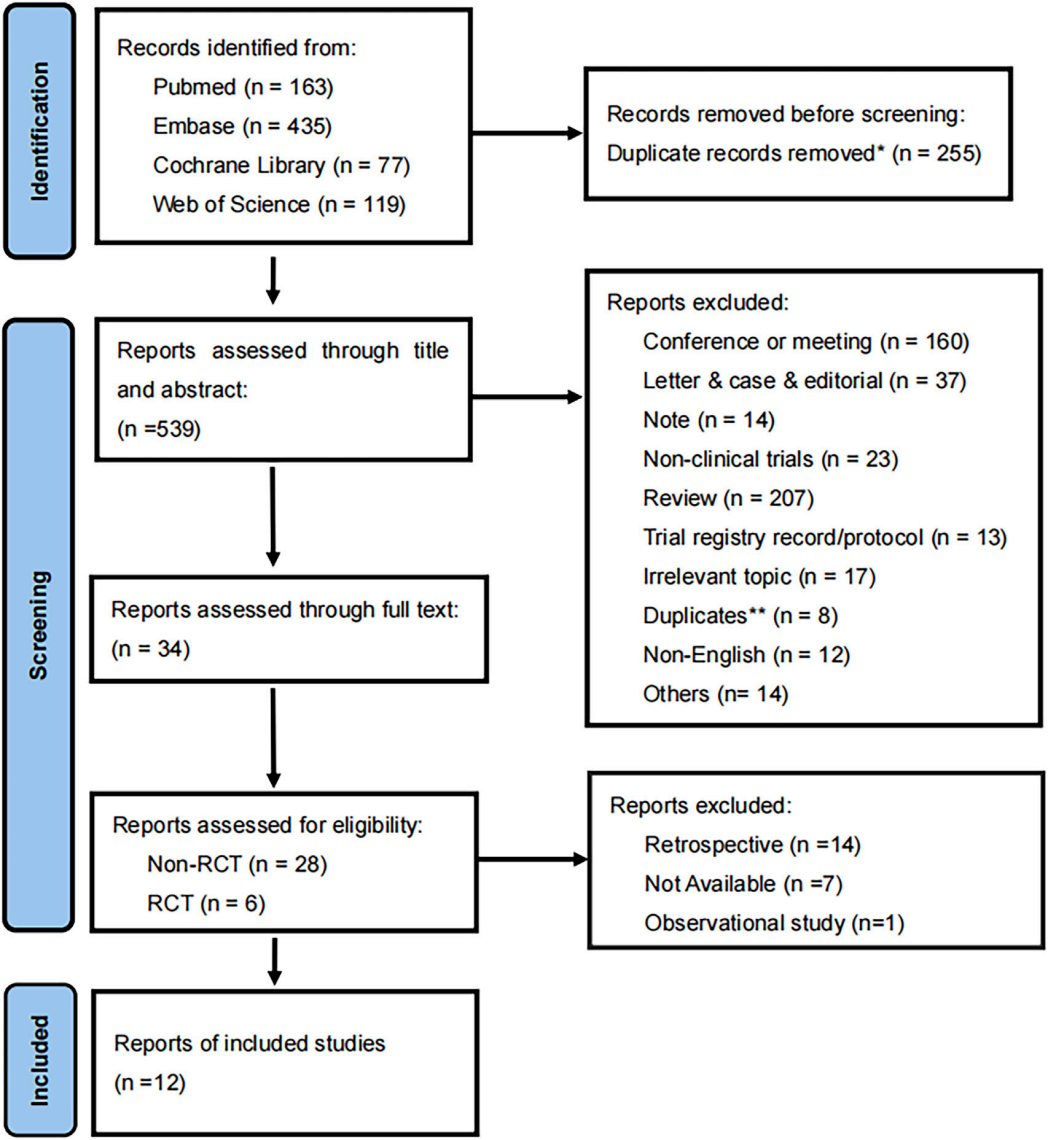
Flow diagram of the meta-analysis for inclusion and exclusion of studies. *255 were excluded by automation tools. **8 were excluded by two reviewers.

Padua et al. conducted a scoping review on the applications of ADCs in urothelial cancer, following which we initiated a supplementary search term in the screening process to identify potential or already approved ADC drugs that could complement existing treatments for UCs (Padua et al., 2022). The ADC drugs identified in this screening include enfortumab vedotin (EV), sacituzumab govitecan (SG), trastuzumab emtansine (TE), disitamab vedotin (DV), and ASG-15ME (Padua et al., 2022). The search language was restricted to English; additionally, we evaluated the included articles in the further study (Figure 1).

### 2.2 Inclusion and exclusion criteria

The prospective clinical cohorts were considered eligible for inclusion if they satisfied the following criteria: (1) inclusion of patients diagnosed with urothelial carcinoma; (2) investigation of ADCs as monotherapies; (3) inclusion of cohorts within clinical studies employing single- or multi-arm designs; (4) reporting of at least one of the following outcomes: ORR, DCR, PFS, and OS. The tumor responses were evaluated using the response evaluation criteria in solid tumors (RECIST) version 1.1 (Eisenhauer et al., 2009). The exclusion criteria encompassed the following: (1) interventions not involving ADCs; (2) interventions involving concurrent administration of ADCs with other therapeutic agents; (3) studies categorized as reviews, letters, case reports, editorials, conference abstracts, retrospective analyses, and *in vitro*/*vivo* experiments; (4) studies that did not report any helpful outcomes; (5) studies not presented in the English language.

The eligible studies included RCTs and NRSIs in all phases of development, such as the phase I, II, and III clinical trials. All included studies were prospective. The toxic effects were evaluated for their incidence and severity using the common terminology criteria for adverse events (CTCAE) (Trotti et al., 2003).

### 2.3 Data extraction

The required data from all the included studies were independently extracted by two investigators, followed by a quality assessment of these studies. The summarized characteristics included authors, publication year, region, sample size, median age, median follow-up, types of ADCs, dosage, reported endpoints, and other details essential for the analyses. The indexes used for clinical and safety outcomes included ORR, DCR, OS, and PFS, as well as the incidence of any AEs and TRAEs (all grades and grades ≥ III). Furthermore, we applied Engauge Digitizer version 12.1 to extract the 6-month and 1-year survival rates from the Kaplan–Meier curves. Supplementary Table S2 presents these key details.

### 2.4 Quality evaluation and risk of bias (ROB) assessment

RCT studies usually utilize the ROB quality assessment tool to evaluate randomized interventions (Higgins et al., 2011). In contrast, the Newcastle–Ottawa scale (NOS) is employed for NRSIs along with consideration of the heterogeneities among single-arm cohorts. Thus, five RCTs underwent ROB assessments, which encompassed seven key domains to assess the quality of the cohorts from selection, performance, detection, attrition, reporting, and other biases (Supplementary Figure A1). Seven non-randomized cohorts were assessed using the NOS, which categorized these studies into three dimensions based on eight items, including population election, comparability, and outcome or exposure evaluation (Supplementary Table A1) (Stang, 2010).

Furthermore, the quality assessments were conducted independently by two authors, and resolution was achieved through discussion with a third party in the case of discrepancies. Throughout the evaluation process, opinions were also sought from ChatGPT 3.5 as a reference; the final decisions on adoption or rejection of these opinions were contingent upon joint assessments by the three authors.

### 2.5 Statistical analysis

The data were analyzed using RStudio (version 2023.09.0 + 463) and R (version 4.3.1). The *p*-value and I^2^ statistics were used to check heterogeneity. A *p*-value less than 0.05 was deemed to be statistically significant. Considering the notable proportion of single-arm cohorts, a random-effects model was employed to consolidate the results. The funnel plot, asymmetry test, and trim-and-fill method were also employed to assess the presence of publication bias and detect potential asymmetries in the effect sizes. Subgroup analyses were performed to obtain additional insights by considering the ADC classes, dosage, and other relevant factors. Sensitivity analysis was performed by excluding studies that significantly influenced the overall results and reanalyzing the data. The R code for the ORR subgroup analysis is available in the Supplementary Method S1.

## 3 Results

Twelve prospective clinical studies covering four types of ADC drugs along with five RCTs and seven single-arm cohorts, involving a collective population of 1,311 patients diagnosed with urothelial cell carcinoma, were included in the meta-analysis that focuses on the administration of ADCs as monotherapy. These clinical cohorts consisted of two phase I, two phase I/II, five phase II, and three phase III trials. The ADCs meeting the inclusion criteria were EV, TE, DV, and SG, and the clinical trials were published from 2019 to 2023, whose characteristics and findings are included in Table 1(Matsubara et al., 2023; de Vries et al., 2023; O'Donnell et al., 2023; Rosenberg et al., 2023; Sheng et al., 2021; Powles et al., 2021; Yu et al., 2021; Tagawa et al., 2021; Bardia et al., 2021b; Rosenberg et al., 2020; Takahashi et al., 2020; Rosenberg et al., 2019).

**TABLE 1:** Characteristics of the clinical trials included in this study.

Study	No. of pts	ADC	PFS			OS			CR	PR	AE	TRAE	
			mPFS (95% CI), mo	PFS, 6 m, %	PFS, 12 m, %	mOS (95% CI), mo	OS, 6 m, %	OS, 12 m, %			n	n	Death
Matsubara et al. (2023)	36	EV	6.47 (5.39, 12.94)	54	32	15.18 (11.56, -)	94.28	67.2	2	9	33	NR	NR
de Vries et al. (2023)	13	TE	2.20 (1.18, 4.30)	NR	NR	7.03 (3.75, -)	NR	NR	0	5	11	11	0
	7	TE	NR	NR	NR	NR	NR	NR	0	4	7	7	0
	6	TE	NR	NR	NR	NR	NR	NR	0	1	4	4	0
O'Donnell et al. (2023)	73	EV	8.0 (6.05, 10.34)	62.4	35.8 (21.86, 49.89)	21.7 (15.20, -)	83.6	70.7 (58.12, 89.9)	3	30	NR	NR	2
Rosenberg et al. (2023)	301	EV	5.55 (5.32, 6.28)	33.3	18.26	12.91 (11.01, 14.92)	78	53	20	99	NR	278	7
Sheng et al. (2021)	43	DV	6.9 (5.6, 8.9)	59.10 (42.60, 72.30)	21.9	13.9 (9.1, -)	84	55.8 (39.8, 69.1)	0	22	NR	43	0
Powles et al. (2021)	301	EV	5.55 (5.32, 5.82)	44	21.7	12.88 (10.58, 15.21)	78	51.5 (44.6, 58.0)	14	103	290	278	7
Yu et al. (2021)	89	EV	5.8 (5.03, 8.28)	50 (38.60, 60.40)	33 (21.9, 43.6)	14.7 (10.51, 18.20)	85.3	59.4	18	28	89	86	3
Tagawa et al. (2021)	113	SG	5.4 (3.5, 7.2)	44.1	12.5	10.9 (9.0, 13.8)	66.6	45	6	25	111	107	1
Bardia et al. (2021)	45	SG	6.8 (3.6, 9.7)	NR	NR	16.8 (9.0, 21.9)	NR	NR	2	11	NR	NR	NR
Rosenberg et al. (2020)	155	EV	NR	NR	NR	NR	NR	NR	8	49	NR	145	NR
	112	EV	5.4 (5.06, 6.28)	42.3	19.3	12.3 (9.33, 15.31)	74	51.8	5	43	NR	NR	NR
	27	EV	NR	NR	NR	NR	NR	NR	3	2	NR	NR	NR
	14	EV	NR	NR	NR	NR	NR	NR	0	3	NR	NR	NR
	2	EV	NR	NR	NR	NR	NR	NR	0	1	NR	NR	NR
	23	EV	NR	NR	NR	NR	NR	NR	2	8	NR	NR	NR
	89	EV	NR	NR	NR	12.3 (9.33, 16.10)	76	51	3	35	NR	NR	NR
	74	EV	6.6 (5.32, 8.15)	53.3	24.4	NR	NR	NR	8	25	NR	NR	NR
Takahashi et al. (2020)	17	EV	8.1 (3.5, -)	NR	NR	NR	NR	NR	1	5	NR	4	NR
	9	EV	NR	NR	NR	NR	NR	NR	1	3	NR	2	NR
	8	EV	NR	NR	NR	NR	NR	NR	0	2	NR	2	NR
Rosenberg et al. (2019)	125	EV	5.8 (4.9, 7.5)	49.5	18.35	11.7 (9.1, -)	79.7	49.8	15	40	125	117	0

^a^ The same study may contain multiple cohorts, with information from different cohorts presented separately. EV, enfortumab vedotin; TE, trastuzumab emtansine; DV, disitamab vedotin; SG, sacituzumab govitecan; mPFS, median progression-free survival; mOS, median overall survival; CR, complete response; PR, partial response; AE, adverse event; TRAE, treatment-related adverse event; NR, not reported.

### 3.1 Efficacy

#### 3.1.1 Pooled ORR

**FIGURE 2:**
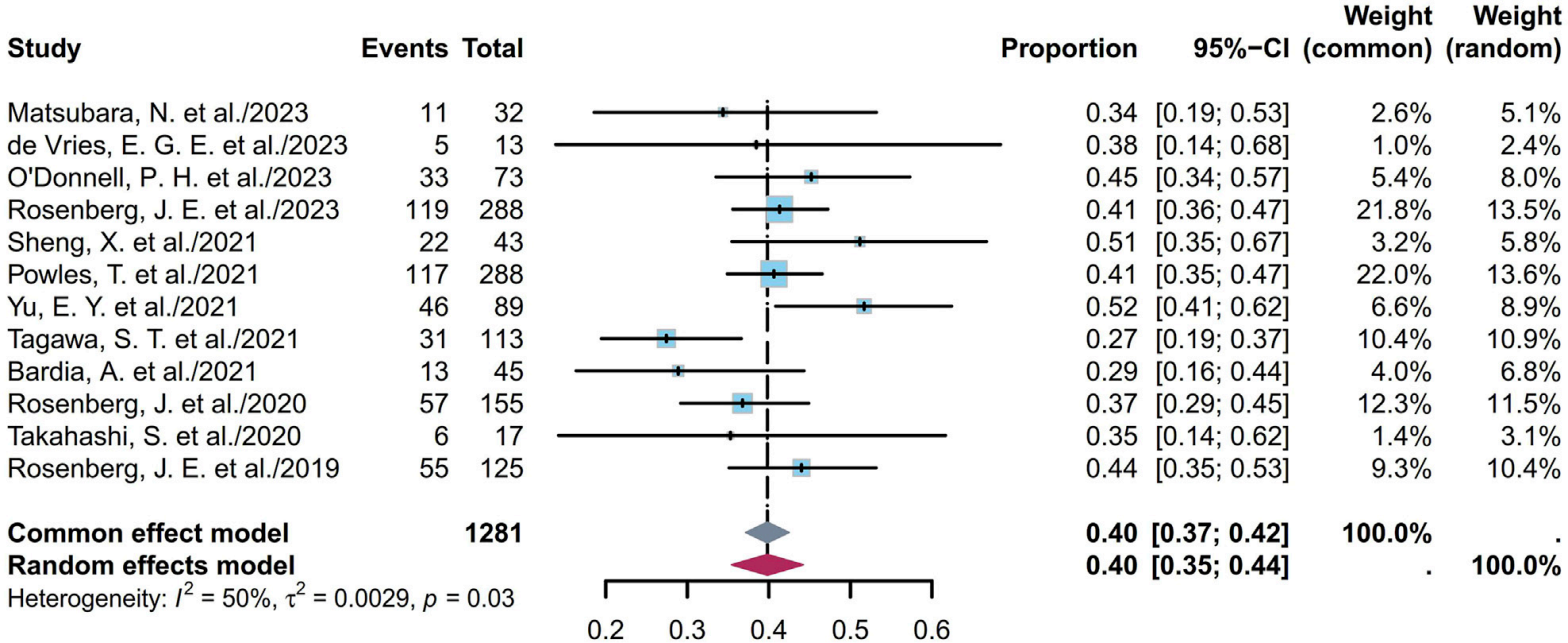
Forest plot of the ORR depicted using the sample-size-weighted random-effects model. ORR, objective response rate; CI, confidence interval.

**FIGURE 3:**
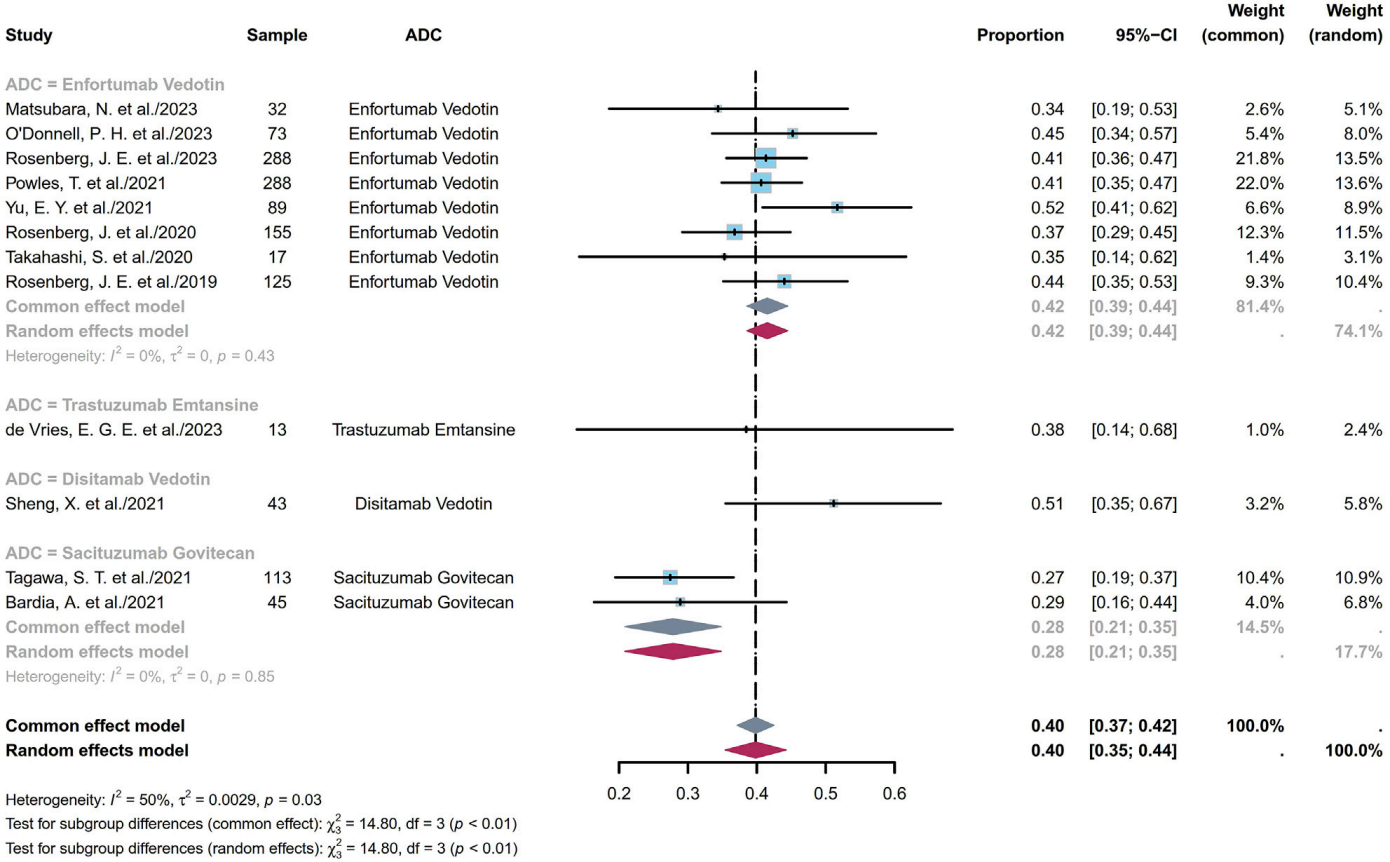
Forest plot of the subgroup analysis of ORR depicted based on the sample-size-weighted random-effects model. ORR, objective response rate; CI, confidence interval.

ORR refers to the proportion of patients whose tumor volumes shrank to a predetermined value that was maintained over a minimum time duration. Twelve studies comprising 1,281 patients provided ORR information ranging from 27% to 52%. The pooled ORR was calculated as 40% (95% confidence interval (CI) [0.35, 0.44]; I^2^ = 50%; *p* = 0.03; Figure 2). Supplementary Figures S1A, B depict the funnel plot and Egger test result for the ORR, with a *p*-value of 0.962 indicating no evidence of publication bias. Subgroup analysis was conducted based on the ADC classes (Figure 3), revealing that any heterogeneity stemmed from the different ADC types. Furthermore, as some studies share the same ClinicalTrials.gov identifier, subgroup analysis was performed based on the identifier (Supplementary Figure S2), demonstrating that heterogeneity arose mainly from the diversity in the clinical study designs. Additional subgroup analyses, based on the classification of studies (Supplementary Figures S3, S4), revealed high consistency within the RCT group (I^2^ = 0; *p* = 0.84) and high heterogeneity in the non-RCT group (I^2^ = 69%; *p* < 0.01). This discrepancy is attributable to the fact that RCTs primarily focus on EV studies, whereas non-RCTs involve various ADCs and exhibit differences among the single-arm studies. Considering different image assessments within one study, eight EV studies were further extracted and categorized into blinded independent central review (BICR) and investigator review (IA) groups for analyses (Supplementary Figures S5, S6). The results show low heterogeneities in both groups, with higher internal consistency in the BICR group (I^2^ = 0; *p* = 0.71). The twelve clinical reports encompassed 17 cohorts with varying ADCs and their dosages. The EV cohorts with the most abundant data were chosen for subgroup analysis based on dosage (Supplementary Figure S7); these results revealed a remarkably low heterogeneity in the 1.25 mg/kg subgroup (I^2^ = 0; *p* = 0.46), indicating that a therapeutic dosage of 1.25 mg/kg would be ideal for EV. After grouping the 17 cohorts based on the classification of studies, a subgroup analysis was conducted on the dosage. Compared with the previous analysis, the RCT subgroup remained largely consistent (Supplementary Figure S8). In the non-RCT ADC subgroup, the heterogeneity of the TE subgroup was attributed to the small sample sizes in the original studies, whereas that of the EV subgroup could be a result of the varied doses and small sample sizes (Supplementary Figure S9). In the EV dosage analysis of the non-RCT group, there was high heterogeneity among the different doses, suggesting that the dosage could be a key variable contributing to variations in EV efficacy across studies (Supplementary Figure S10).

#### 3.1.2 Pooled DCR

**FIGURE 4:**
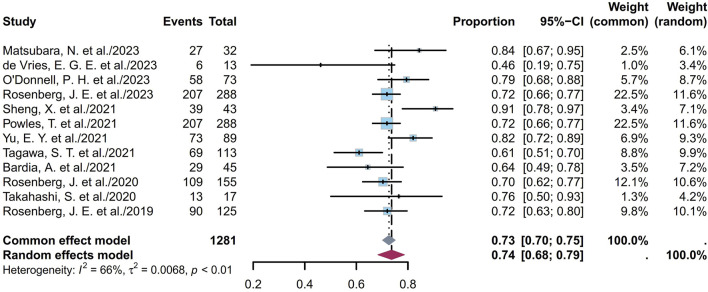
Forest plot of the DCR depicted using the sample-size-weighted random effects model. DCR, disease control rate; CI, confidence interval.

**FIGURE 5:**
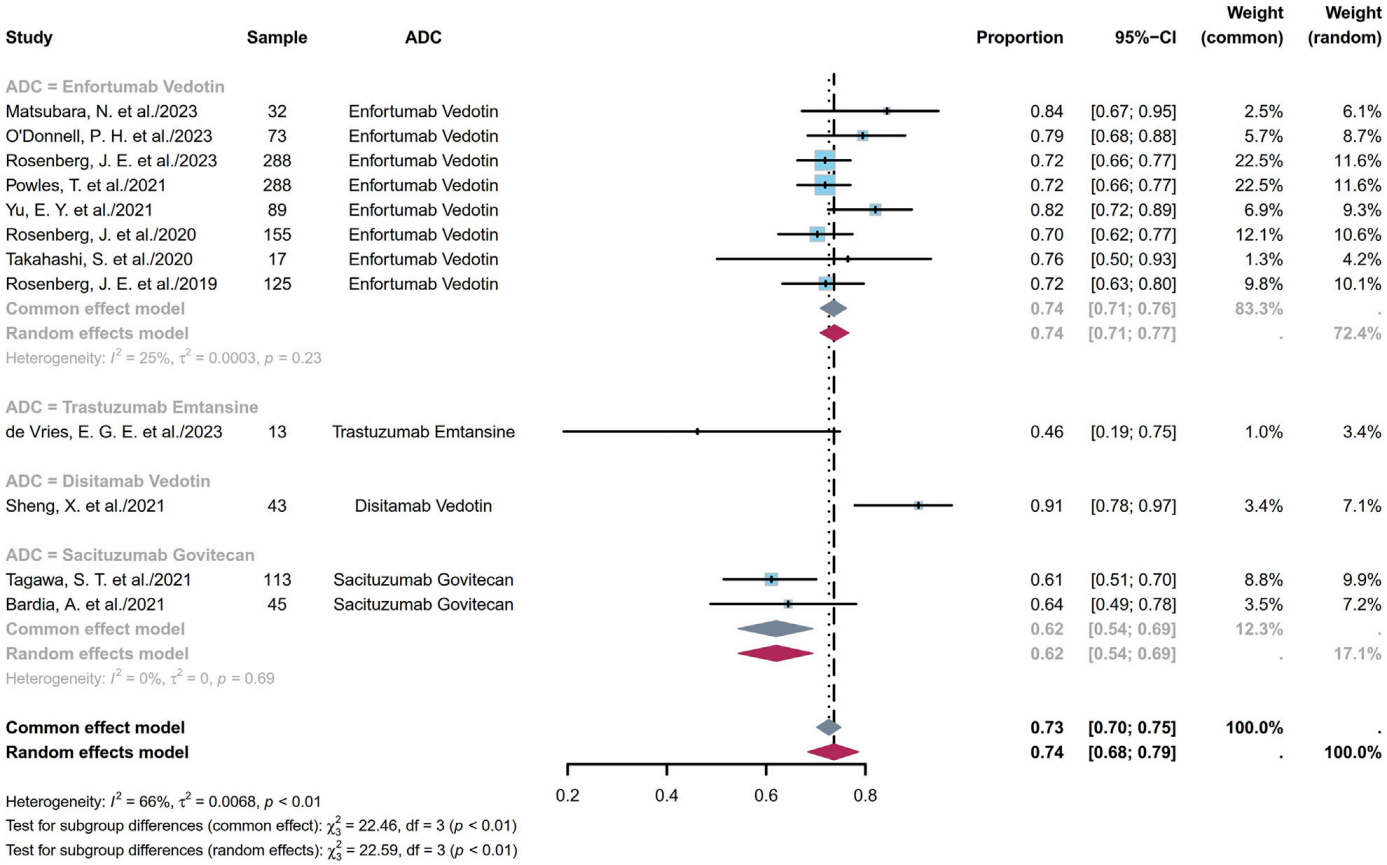
Forest plot of the subgroup analysis of DCR depicted using the sample-size-weighted random effects model. DCR, disease control rate; CI, confidence interval.

The DCR indicates the proportion of patients who achieve either a complete response (CR), a partial response (PR), or stable disease (SD). The twelve studies comprised 1,281 patients with DCRs ranging from 46% to 91%; the pooled DCR was calculated to be 74% (95% CI [0.68, 0.79]; I^2^ = 66%; *p* < 0.01; Figure 4). Supplementary Figures S11A, B depict the funnel plot and Egger test result for DCR, with a *p*-value of 0.5844 indicating no evidence of publication bias. Subgroup analysis was conducted for the ADCs (Figure 5), revealing that the heterogeneity stems from different ADC types. Furthermore, as some studies shared the same ClinicalTrials.gov identifier, subgroup analysis was performed based on the identifier (Supplementary Figure S12), demonstrating that the heterogeneity arose from diversity in the study designs and clinical background. Interestingly, the heterogeneity contributions from the two trials originating from the same identifier (NCT03219333) were not evident in the ORR analysis but manifested clearly for the DCR (Supplementary Figures S2, S12). Additional subgroup analyses based on the classification of studies (Supplementary Figures S13, S14) revealed low heterogeneity within the RCT group (I^2^ = 30%; *p* = 0.22) and high heterogeneity among the non-RCT group (I^2^ = 81%; *p* < 0.01). This discrepancy may also have the same reason as that explained for the ORR. Eight EV studies were categorized into BICR or IA groups for further analyses (Supplementary Figures S15, S16); their results showed low heterogeneity in the BICR group (I^2^ = 15%; *p* = 0.31) and high heterogeneity in the IA group (I^2^ = 56%; *p* = 0.03). The funnel plot and Egger test result of the IA group with a *p*-value of 0.4184 signify the absence of publication bias (Supplementary Figures S17A, B). This heterogeneity could arise from differences in the studies or researcher bias in the IA group.

The EV cohorts of the 17 cohorts were chosen for subgroup analyses based on dosage (Supplementary Figure S18). The results revealed mild heterogeneity in the 1.25 mg/kg subgroup (I^2^ = 34%; *p* = 0.17). Furthermore, a subgroup analysis of the dosage in the RCT group was conducted (I^2^ = 76%; *p* < 0.01; Supplementary Figure S19), whose funnel plot and Egger test with a *p*-value of 0.3910 indicate no evidence of publication bias (Supplementary Figures S20A, B). This implies that the heterogeneity is primarily due to dosage variations. In the non-RCT group, high heterogeneity of the TE subgroup was observed, which could be due to the small sample sizes in the original studies (Supplementary Figure S21). In the EV cohorts of the non-RCT group, instead of the high heterogeneity observed for ORR, the DCR showed low heterogeneity (Supplementary Figures S9, S21). Likewise, instead of the high heterogeneity in the ORR (Supplementary Figure S10), the DCR demonstrated low heterogeneity across studies in the EV dosage analysis of the non-RCT group (I^2^ = 18%; *p* = 0.3; Supplementary Figure S22). These results could be stochastic events or indicative of the fact that despite ORR variations, different dosages of EV tend to exhibit consistent DCRs. In summary, in the dosage analysis of the EV cohorts, DCR exhibited high heterogeneity in the RCT group compared to the non-RCT group. Furthermore, ORR displayed low heterogeneity compared to DCR in the RCT group, whereas the opposite was observed in the non-RCT group.

#### 3.1.3 Pooled PFS

PFS is defined as the duration from the start of the study treatment to the appearance of objective tumor progression or death from any cause, whichever comes first. The twelve studies included 1,268 patients, whose pooled median PFS was 5.66 months (95% CI [4.89, 6.43]; I^2^ = 59%; *p* < 0.01; Supplementary Figure S23). Supplementary Figures S24A and S24B depict the corresponding funnel plot and Egger test result with a *p*-value of 0.6519, denoting no evidence of publication bias. Further sensitivity analysis revealed that the TE studies were the primary source of the heterogeneity (Supplementary Figure S25), indicating potential divergent efficacy within the TE subgroup (de Vries et al., 2023). Subgroup analyses of the different ADCs revealed that the heterogeneity was primarily attributable to the ADC type (Supplementary Figure S26). Based on classification according to dosage in the EV cohorts, the pooled median PFS was 5.57 months (95% CI [5.37, 5.77]; I^2^ = 0; *p* = 0.53; Supplementary Figure S27).

**FIGURE 6:**
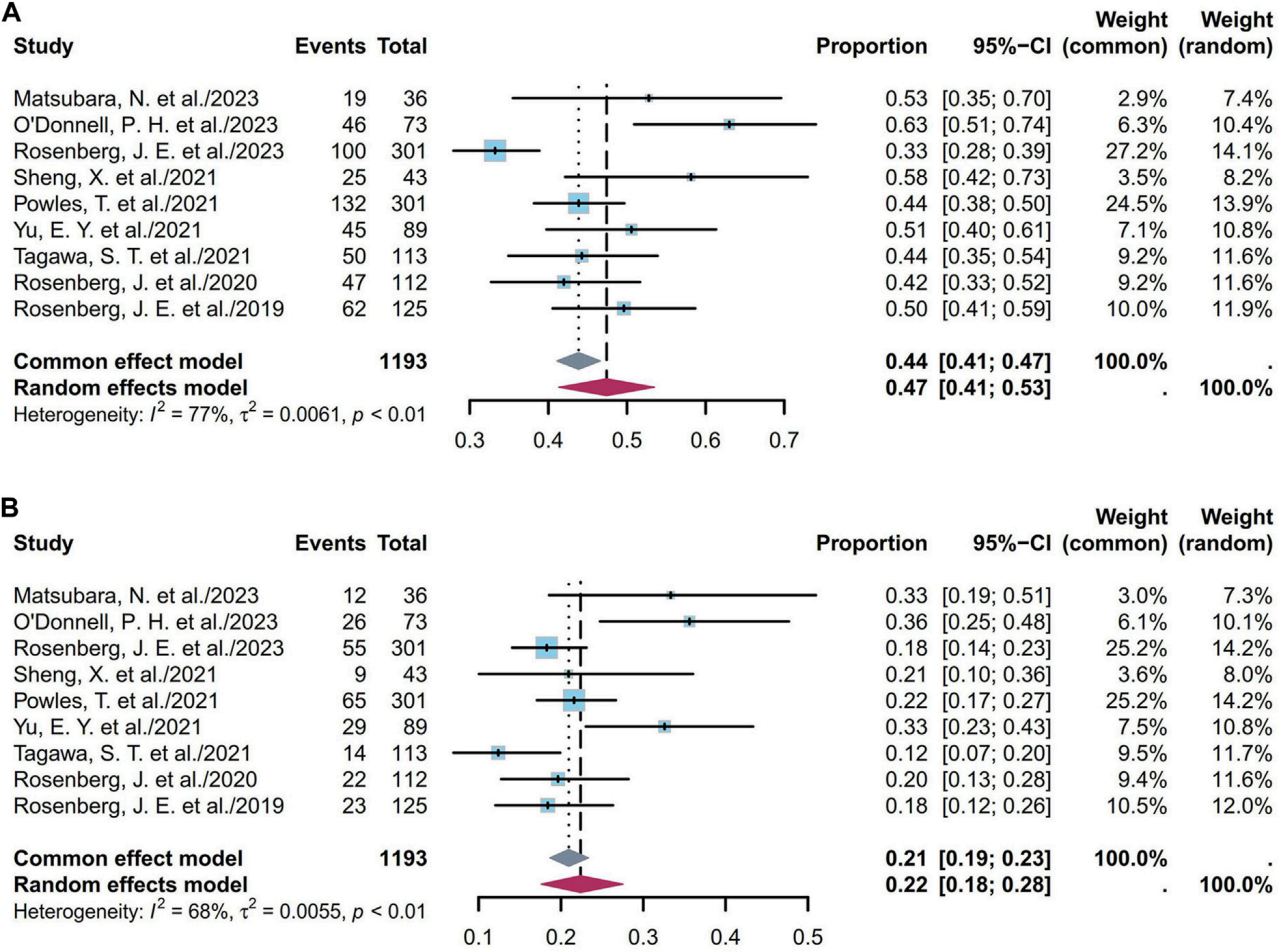
Forest plot of the PFS rate: **(A)** 6-month PFS; **(B)** 1-year PFS. The sample-size-weighted random-effects model was applied to depict the forest plot. PFS, progression-free survival; CI, confidence interval.

Nine studies including 1,193 patients indicated that the 6-month PFS ranged from 33% to 63%. Figure 6A depicts the forest plot of the 6-month PFS, where the pooled 6-month PFS was 47% (95% CI [0.41, 0.53]; I^2^ = 77%; *p* < 0.01). Supplementary Figures S28A and S28B depict the corresponding funnel plot and Egger test with a *p*-value of 0.016, denoting the presence of publication bias. Subgroup analyses of the different ADCs revealed that the heterogeneity was primarily attributable to the EV cohorts (Supplementary Figure S29). Classification based on dosage among the EV cohorts showed that the pooled 6-month PFS was 47% (95% CI [0.4, 0.54]; I^2^ = 81%; *p* < 0.01; Supplementary Figure S30). Nine studies including 1,193 patients indicated that the 1-year PFS ranged from 12% to 36%. Figure 6B depicts the forest plot for the 1-year PFS, and the pooled 1-year PFS was 22% (95% CI [0.18, 0.28]; I^2^ = 68%; *p* < 0.01). Supplementary Figures S31A and S31B depict the corresponding funnel plot and Egger test result with a *p*-value of 0.2122, denoting no presence of publication bias. Subgroup analyses of the different ADCs revealed that the heterogeneity was primarily attributable to the EV cohorts (Supplementary Figure S32). Classification based on dosage among the EV cohorts showed that the pooled 1-year PFS was 24% (95% CI [0.19, 0.30]; I^2^ = 67%; *p* < 0.01; Supplementary Figure S33). Overall, the pooled 6-month and 1-year PFS rates were 47% and 22%, respectively.

#### 3.1.4 Pooled OS

OS is defined as the time from the start of the study treatment to the date of death from any cause. Eleven studies including 1,251 patients showed that the pooled median OS was 12.63 months (95% CI [11.64, 13.63]; I^2^ = 42%; *p* = 0.07; Supplementary Figure S34). Supplementary Figures S35A and S35B depict the corresponding funnel plot and Egger test result with a *p*-value of 0.2154, denoting no evidence of publication bias. Furthermore, a sensitivity analysis revealed that TE studies were the primary source of the heterogeneity (Supplementary Figure S36) (de Vries et al., 2023); this heterogeneity may also have the same reason as that explained for the PFS. Subgroup analyses of the different ADCs indicate that the heterogeneity arises from both the ADC type and SG cohorts (Supplementary Figure S37). Classification based on dosage in the EV cohorts showed that the pooled median OS was 13.13 months (95% CI [12.02, 14.24]; I^2^ = 19%; *p* = 0.29; Supplementary Figure S38).

**FIGURE 7:**
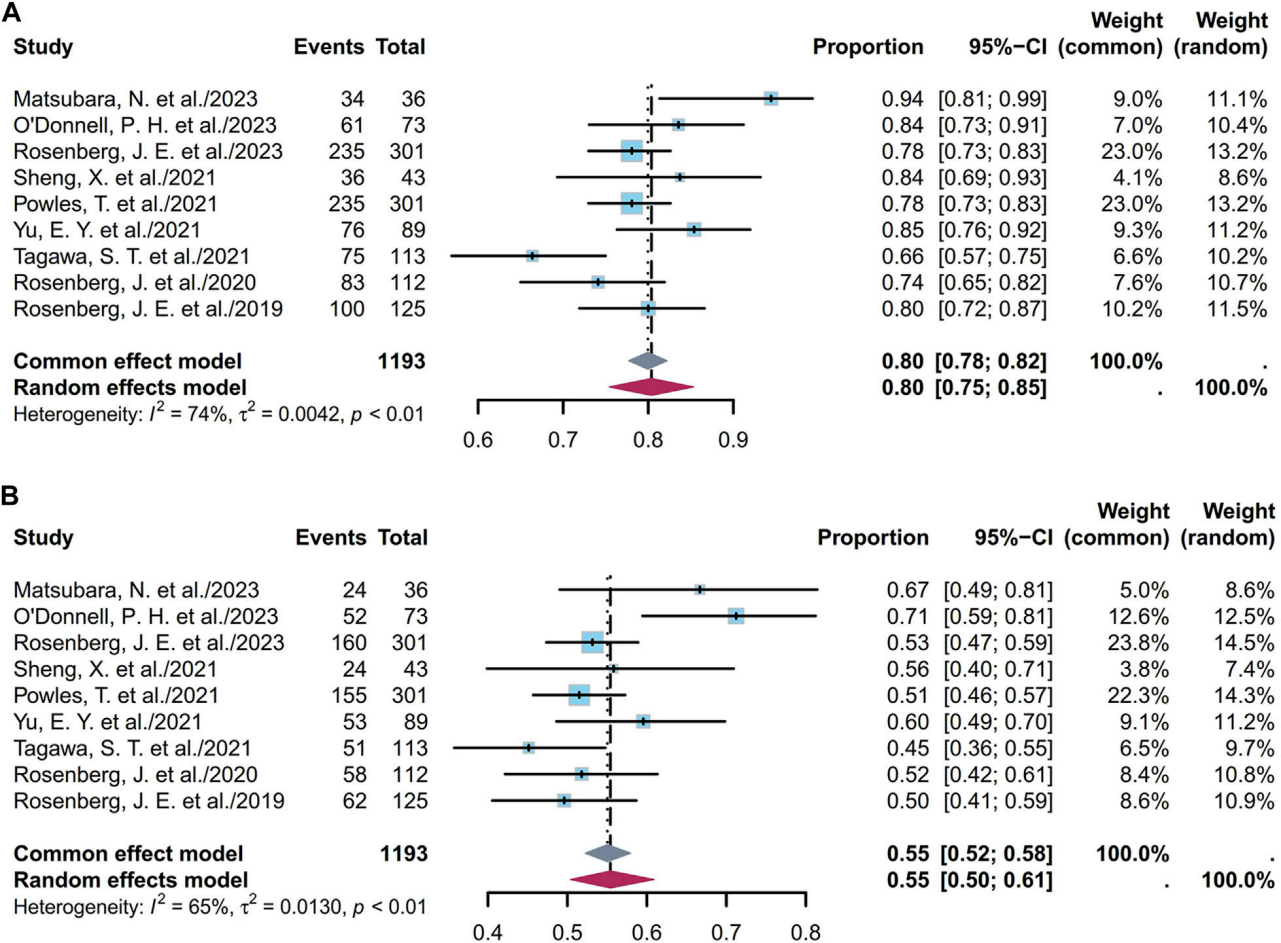
Forest plot of the OS rate: **(A)** 6-month OS; **(B)** 1-year OS. The sample-size-weighted random-effects model was applied to depict the forest plot. OS, overall survival; CI, confidence interval.

Nine studies including 1,193 patients indicated that the 6-month OS ranged from 66% to 94%. Figure 7A depicts the forest plot for the 6-month OS, and the pooled 6-month OS was 80% (95% CI [0.75, 0.85]; I^2^ = 74%; *p* < 0.01). Supplementary Figures S39A and S39B depict the corresponding funnel plot and Egger test result with a *p*-value of 0.691, denoting no presence of publication bias. Subgroup analyses of the different ADCs revealed that the heterogeneity was primarily attributable to the EV cohorts (Supplementary Figure S40). Classification based on dosage in the EV cohorts showed that the pooled 6-month OS was 82% (95% CI [0.77, 0.87]; I^2^ = 72%; *p* < 0.01; Supplementary Figure S41). Nine studies including 1,193 patients indicated that the 1-year OS ranged from 12% to 36%. Figure 7B depicts the forest plot for the 1-year OS, and the pooled 1-year OS was 55% (95% CI [0.50, 0.61]; I^2^ = 65%; *p* < 0.01). Supplementary Figures S42A and S42B depict the corresponding funnel plot and Egger test result with a *p*-value of 0.7344, denoting no presence of publication bias. Subgroup analyses of the different ADCs revealed that the heterogeneity was primarily attributable to the EV cohorts (Supplementary Figure S43). Classification based on dosage in the EV cohorts showed that the pooled 1-year OS was 57% (95% CI [0.51, 0.63]; I^2^ = 68%; *p* < 0.01; Supplementary Figure S44). Overall, the pooled 6-month and 1-year OS rates were 80% and 55%, respectively.

#### 3.1.5 Pooled duration of response (DOR) and duration of treatment (DOT)

The DOR refers to the time from the date of the first documented response to the date of the first documented objective tumor progression or death from any cause, whichever comes first. Eleven studies including 1,289 patients showed that the pooled median DOR was 7.39 months (95% CI [6.17, 8.62]; I^2^ = 71%; *p* < 0.01; Supplementary Figure S45). Supplementary Figures S46A and S46B depict the corresponding funnel plot and Egger test result with a *p*-value of 0.0318, denoting the evidence of publication bias. After applying the trim-and-fill method, the pooled median DOR was 6.49 months (95% CI [5.18, 7.79]; I^2^ = 71.2%; *p* < 0.01; Supplementary Figure S47). Furthermore, a sensitivity analysis revealed that the TE studies were the primary source of the heterogeneity (Supplementary Figure S48) (de Vries et al., 2023), indicating potential divergent efficacy within the TE subgroup. Subgroup analyses of the different ADCs revealed that the heterogeneity primarily stemmed from the distinct drug classes, with high internal consistency in each class (Supplementary Figure S49). In the dosage analysis of the EV cohorts, the highest consistency was observed in the 1.25 mg/kg group (Supplementary Figure S50).

DOT refers to the period that a patient undergoes a specific treatment before the disease progresses or ceases to respond to treatment. Eleven studies including 1,071 patients showed that the pooled median DOT was 5.06 months (95% CI [4.50, 5.62]; I^2^ = 50%; *p* = 0.05; Supplementary Figure S51). Supplementary Figures S52A and S52B depict the corresponding funnel plot and Egger test result with a *p*-value of 0.877, denoting no evidence of publication bias. Furthermore, a sensitivity analysis revealed that the SG studies were the primary source of the heterogeneity, indicating potential divergent efficacy within the SG subgroup (Supplementary Figure S53) (Tagawa et al., 2021). Subgroup analyses of the different ADCs revealed that the moderate heterogeneity primarily stemmed from the distinct drug classes and that the EV group had high internal consistency (Supplementary Figure S54). In the dosage analysis of the EV cohorts, the highest consistency was observed in the 1.25 mg/kg group (Supplementary Figure S55).

### 3.2 Safety

**FIGURE 8:**
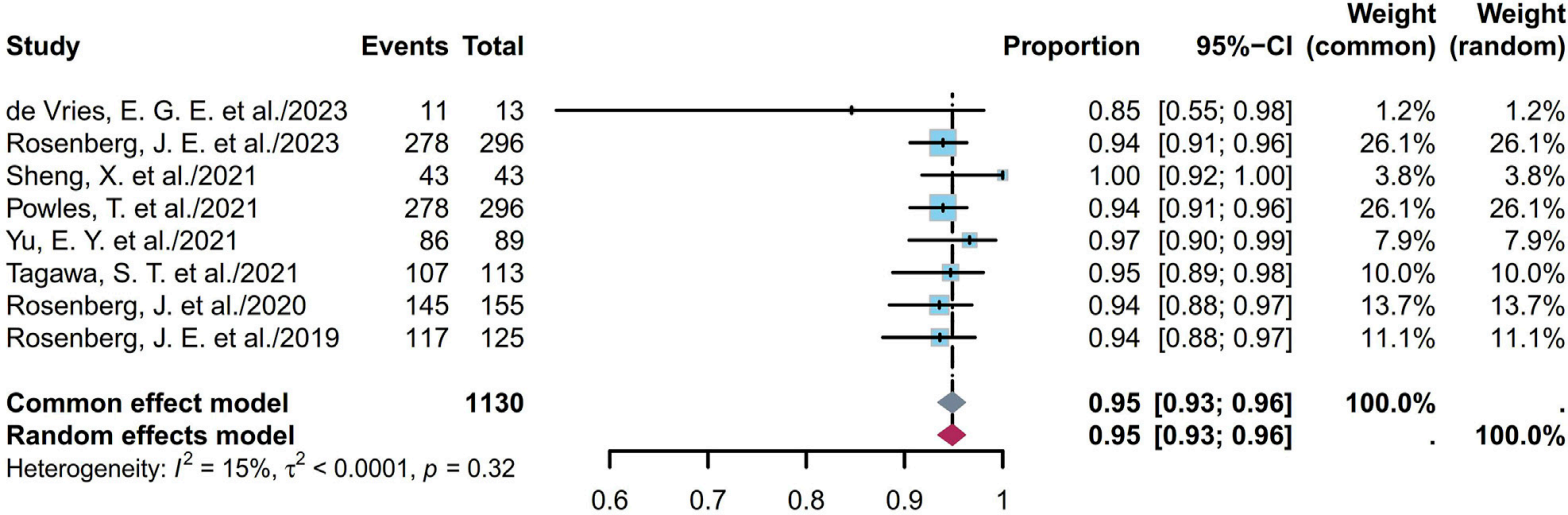
Forest plot of TRAEs rate (after excluding an outlier study). The sample-size-weighted random-effects model was applied to depict the forest plot. TRAEs, treatment-related adverse events.

**FIGURE 9:**
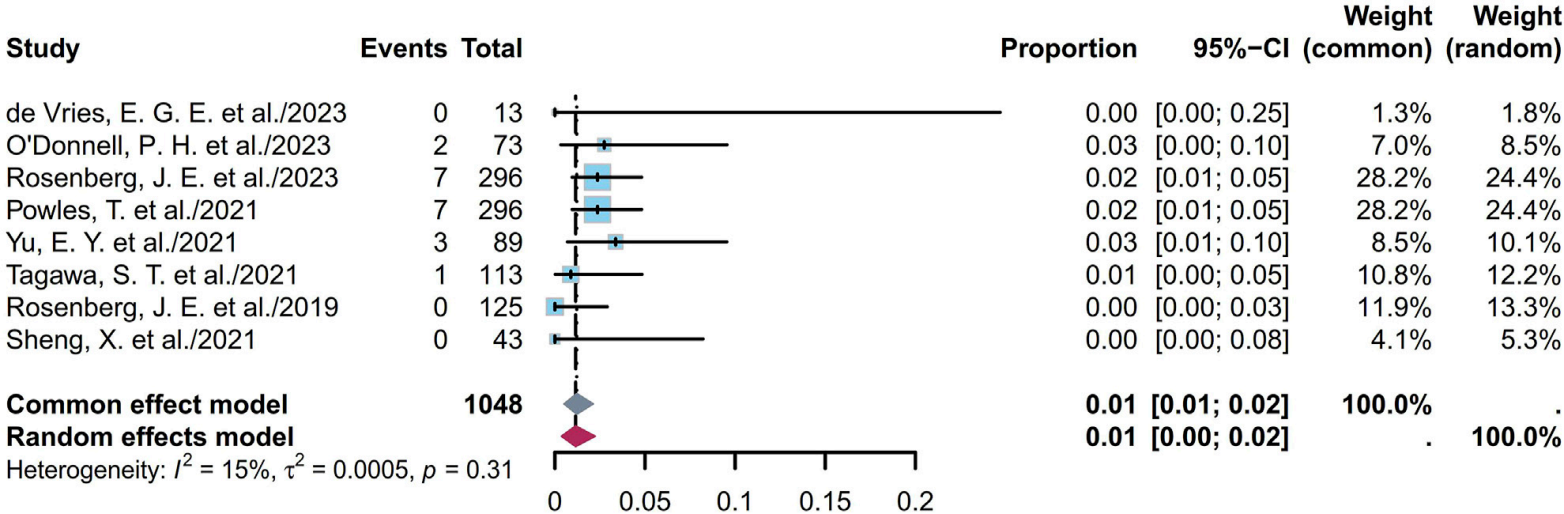
Forest plot of TRAEs leading to death rate. The sample-size-weighted random-effects model was applied to depict the forest plot. TRAEs, treatment-related adverse events.

Some endpoints, such as the AEs, TRAEs (all grades and grades ≥ III), TRAEs leading to death, and the most common TRAEs, were analyzed. Six studies involving 672 patients showed that the prevalence of AEs ranged from 85% to 100%, with a pooled rate of 99% (95% CI [0.95, 1.00]; I^2^ = 69%; *p* < 0.01; Supplementary Figure S56). Eleven studies involving 1,147 patients showed that the prevalence of TRAEs ranged from 85% to 100%, with a pooled rate of 91% (95% CI [0.79, 0.99]; I^2^ = 85%; *p* < 0.01; Supplementary Figure S57). In the sensitivity analysis, exclusion of an outlier study resulted in a significant reduction in the heterogeneity (95% CI [0.93, 0.96]; I^2^ = 15%; *p* = 0.32; Figure 8); this could be attributed to the small sample sizes or researcher bias. Six studies involving 1,034 patients showed that the prevalence of TRAEs with grades ≥ III ranged from 34% to 55%, with a pooled rate of 49% (95% CI [0.43, 0.56]; I^2^ = 73%; *p* < 0.01; Supplementary Figure S58). Seven studies involving 1,048 patients showed that the prevalence of TRAEs leading to death ranged from 0% to 3%, with a pooled rate of 1% (95% CI [0.00, 0.02]; I^2^ = 15%; *p* = 0.31; Figure 9). The corresponding funnel plots and Egger test results of these endpoints are presented in Supplementary Figure S59. Five of the studies reported TRAEs leading to death. The disclosed fatal TRAEs include multiorgan dysfunction syndrome, abnormal hepatic function, hyperglycemia, pelvic abscess, pneumonia, septic shock, acute kidney injury, metabolic acidosis, and febrile neutropenia. Two patients experienced multiorgan dysfunction syndrome, while the remaining events occurred in one patient each.

The seven most common TRAEs documented in the included studies were alopecia, decreased appetite, dysgeusia, fatigue, nausea, peripheral sensory neuropathy, and pruritus (Table 2), with the pooled rates for all grades being 45%, 34%, 40%, 39%, 45%, 37%, and 32% (I^2^ ranging from 0% to 85%) while the rates for grades ≥ III were 0, 3%, 0, 5%, 2%, 2%, and 1% (I^2^ ranging from 0% to 54%), respectively (Supplementary Figures S60A, B).

**TABLE 2:** Most common TRAEs reported in the included studies.

Study	Sample	Grade	1	2	3	4	5	6	7
Matsubara et al. (2023)	36	Any grade	19	9	18	NR	NR	17	NR
		Grade ≥3	0	2	0	NR	NR	0	NR
O'Donnell et al. (2023)	73	Any grade	26	NR	NR	29	NR	32	19
		Grade ≥3	0	NR	NR	6	NR	2	1
Rosenberg et al. (2023)	296	Any grade	135	92	NR	93	NR	103	96
		Grade ≥3	NR	9	NR	20	NR	15	4
Sheng et al. (2021)	43	Any grade	24	NR	NR	NR	NR	NR	NR
		Grade ≥3	1	NR	NR	NR	NR	NR	NR
Powles et al. (2021)	296	Any grade	134	91	NR	92	NR	100	95
		Grade ≥3	0	9	NR	19	NR	9	4
Yu et al. (2021)	89	Any grade	45	29	NR	30	NR	39	27
		Grade ≥3	0	5	NR	6	NR	3	3
Tagawa et al. (2021)	113	Any grade	47	NR	NR	52	60	NR	NR
		Grade ≥3	0	NR	NR	4	4	NR	NR
Rosenberg et al. (2020)	155	Any grade	61	56	52	71	58	55	NR
		Grade ≥3	0	1	0	3	1	1	NR
Takahashi et al. (2020)	17	Any grade	9	NR	9	NR	NR	NR	8
		Grade ≥3	NR	NR	NR	NR	NR	NR	NR
Rosenberg et al. (2019)	125	Any grade	61	55	50	62	NR	50	NR
		Grade ≥3	0	3	0	7	4	2	NR

^a^ This summary encompasses all TRAEs, occurring in 10% or more of the patients, covering all grades and grades ≥ III TRAEs. TRAEs, treatment-related adverse events; 1-Alopecia; 2-Decreased appetite; 3-Dysgeusia; 4-Fatigue; 5-Nausea; 6-Peripheral sensory neuropathy; 7-Pruritus.

## 4 Discussion

Although the concept of ADCs has been around for several years, the number of successfully implemented ADCs in clinical use remains limited. Given the progression of clinical experiments, it is imperative to build high-quality evidence evaluations. This meta-analysis presents the efficacy and safety of ADCs in patients with advanced or metastatic UC, providing evidence for their future clinical applications. Our study emphasizes pooled data on the clinical response rates, survival rates, occurrence of TRAEs, and fatal TRAEs associated with ADCs.

The efficacy and toxicity characteristics of an ADC are significantly influenced by each of its components. Modifications to any of these components can result in substantial alterations to the clinical profile of the agent (Schlam et al., 2023). The ADCs considered in this analysis include DV, EV, TE, and SG. DV is a newly developed human epidermal growth factor receptor 2 (HER2)-targeting ADC composed of trastuzumab linked to monomethyl auristatin E (MMAE) via a cleavable linker (Shi et al., 2022). EV consists of a fully human monoclonal antibody against nectin cell-adhesion molecule 4 (Nectin-4) and MMAE linked through a linker (Tang et al., 2022). TE is formed by linking trastuzumab and a derivative of maytansine through a linker (Barok et al., 2014). SG is composed of an antitrophoblast cell-surface antigen 2 (TROP2) IgG1 kappa antibody linked to SN-38 (a topoisomerase inhibitor) (Bardia et al., 2021a). ADCs bind to the tumor surface antigens, followed by antigen internalization into the tumor cells through endocytosis. They are then transported to the lysosomes, where the cytotoxic payloads are released. The liberated payloads induce apoptosis through DNA damage or microtubule inhibition and can also kill the adjacent cancer cells via the bystander effect (Jin et al., 2022). Figure 3 illustrates that EV and SG exhibit consistent ORRs across various studies, indicating uniform and stable receptor expressions, particularly by EV, in the treatment of advanced UC patients. Although the data for TE and DV are limited, tumor heterogeneities do not influence the ORRs in patients undergoing HER2-targeting ADC therapy (Lei et al., 2023). This suggests the potential of ADCs to provide consistent therapeutic effects despite the variabilities in tumor biology.

The study population was roughly divided into two categories based on whether they received treatment in the past. Among them, there was only one report of untreated individuals (O'Donnell et al., 2023), while more studies focused on individuals who had previously undergone immunotherapy and/or chemotherapy. Therefore, there is insufficient evidence to recommend standalone ADCs as the first-line therapy, especially for locally advanced or metastatic UC patients who have not been treated previously. However, the latest data indicate that the results of using EV in combination with pembrolizumab are significantly superior to those of chemotherapy in untreated advanced UC patients (Powles et al., 2024). This also suggests a bright future for ADCs, particularly EV, in clinical frontline therapy. Additionally, in treated locally advanced or metastatic UCs, EV alone consistently provides significant survival advantages and safety over standard chemotherapy (Rosenberg et al., 2022; Rosenberg et al., 2023). Several studies have also shown that the safety profile of EV monotherapy is manageable (Rosenberg et al., 2019; Halford et al., 2021; Hoimes et al., 2023). For advanced UC patients who are ineligible for platinum-based chemotherapy, current evidence suggests that treatment with immune checkpoint inhibitors (ICIs) is an alternative for those with programmed death ligand 1 (PD-L1)-positive tumors. Subsequently, EV has emerged as a preferred therapy following prior ICI and chemotherapy regimens (Jones et al., 2024). Based on current evidence, EV alone should be more suitable for such patients after ICI and chemotherapy. Moreover, an ongoing research on intravesical EV for treating BCG-unresponsive populations suggests that EV as an ADC could be a blockbuster drug in the field of urological cancers (Kamat et al., 2023).

One major contributor to heterogeneity among the ADCs, as revealed by our results, is the dosage. In pharmacokinetics, from absorption to excretion, the structure–activity relationships and solvent properties are intricately linked with the drug dosage. For ADCs, the appropriate linker as well as stability and hydrophilicity of the drug molecule are crucial factors. Kasper et al. demonstrated that ethynylphosphonamidate-linked ADCs exhibit high serum stabilities and antitumor activities (Kasper et al., 2019). The linker itself is currently believed to be non-toxic, but its stability significantly influences the subsequent toxicity of the payload (Donaghy, 2016). In terms of the antibodies, site-selective monofunctionalization of IgG enables construction of ADCs carrying a single cytotoxic drug on the heavy chain, facilitating targeted cancer therapy (Chen et al., 2023). Furthermore, engineering modifications of the ADCs to integrate defined quantities of the payload molecules at predetermined antibody sites allow creation of uniform populations of ADCs, leading to improved therapeutic windows, enhanced efficacies, and reduced toxicities (Donaghy, 2016).

In a retrospective real-world study, DV demonstrated favorable efficacy and manageable safety for patients with metastatic UC, regardless of being used as a monotherapy or in combination with programmed cell death-1 (PD-1) inhibitors (Xu et al., 2023). Another prospective real-world study revealed the significant activity of DV in combination with PD-1 inhibitors, particularly in terms of the ORR, for muscle-invasive bladder cancer (MIBC) patients with HER2 immunohistochemistry (IHC) 0/1+/2+/3+ (Wei et al., 2023). The combination of EV with pembrolizumab continued to show promising survival trends in locally advanced or metastatic urothelial carcinoma (la/mUC) patients deemed unsuitable for receiving cisplatin, demonstrating both rapid and durable responses (Gupta et al., 2023). These studies herald a new era in immunotherapy for UCs, including the use of ADCs in combination therapies.

We note that the pooled incidence of TRAEs in ADC therapy was 91%; this aligns with the findings of a meta-analysis by Zhu et al. (2023), which reported a TRAE rate of 91.2%. These results provide crucial guidance for urologic surgeons in managing the toxicities of ADCs in clinical practice. In addition, the rate of fatal TRAEs related to ADCs was 1% in our analysis. Similarly, Fu Z et al. found the risk of fatal TRAEs with HER2-targeted ADCs to be 0.78% (95% CI [0.0028, 0.0137]) compared to standard control treatments in cancer patients (Fu et al., 2023). Although this is not a small enough probability for the overall population, the benefit–risk balance for patients with severe or life-threatening diseases, such as advanced tumors, may favor benefits over the risks of TRAEs. Salvestrini et al. found that although combining TE with postoperative radiotherapy could increase the risk of AEs, the safety of this combination in the treatment of certain cancer patients would be acceptable given the potential benefits(Salvestrini et al., 2023). This aligns with our interpretation.

ADCs have paved the path for targeted therapies (Nishigaki et al., 2020; Drago et al., 2021; Fu et al., 2022; Dumontet et al., 2023; Uchida et al., 2023). In triple-negative diseases, ADCs have shown promising results in phase III trials compared to chemotherapy chosen by the physicians (Rugo et al., 2022). Furthermore, based on a phase II single-arm study, Tisotumab vedotin (Tivdak^®^) was approved in 2021 for gynecological tumors for the treatment of recurrent or metastatic cervical cancer progressing during or after chemotherapy (Coleman et al., 2021). Three ADCs have received approval for use in UCs (Padua et al., 2022). Based on a series of studies, Padcev^®^ (EV) has been approved for la/mUC patients after platinum-based chemotherapy and ICI failure as well as for cisplatin-ineligible individuals after first-line or multiple-line treatments for metastatic UC (Rosenberg et al., 2019; Powles et al., 2021; Yu et al., 2021; Padua et al., 2022). SG has obtained accelerated approval for metastatic UC patients intolerant to platinum-based chemotherapy and ICIs (Tagawa et al., 2021). Finally, EV has received the FDA breakthrough therapy designation for second-line treatment of HER2-expressing advanced UCs (Padua et al., 2022). In addition, for prostate cancers, ADCs are primarily focused on six-transmembrane epithelial antigen of prostate 1 (STEAP1), TROP2, prostate-specific membrane antigen (PSMA), CD46, and B7-H3 as the potential targets (Rosellini et al., 2021). Fahey CC et al. reported a case of metastatic penile squamous cell carcinoma responsive to EV (Fahey et al., 2023); this case supports the use of ADCs, including EV, in squamous cell carcinomas, including penile cancers, due to the observed high expression of Nectin-4 (Fahey et al., 2023; Grass et al., 2023).

In the future, by considering stringent inclusion criteria and high internal consistency within RCTs, further supplementation through large-scale real-world studies is needed in UCs to verify external consistency. Relying solely on a meta-analysis based on rates could reflect the overall efficiencies of ADC drugs. Furthermore, controlled studies including the gold standard of tumor treatment are needed to attain higher levels of evidence and address the question of drug effectiveness definitively. Longer-term follow-ups could also reveal the extended prognosis and adverse reactions associated with ADCs. Meanwhile, there has been a surge in novel ADCs. ADCs with dual payloads hold therapeutic potential in overcoming heterogeneity and drug resistance (Yamazaki et al., 2021). Bispecific ADCs combining the antitumor mechanisms of ADCs with the multifunctionality of bispecific antibodies can address the clinical challenges in ADC development (Gu et al., 2024). Furthermore, dual-targeting agents could include not only bispecific antibodies but also novel bispecific molecules (An et al., 2023). In subsequent research, collaborative designs involving various bispecific molecules and ADCs are expected to usher in a new era of multidimensional targeting. In addition, more ADC formats are under investigation, such as conditionally active ADCs (also known as probody–drug conjugates), immune-stimulating ADCs, and protein-degrader ADCs, each offering unique capabilities to address these diverse challenges (Tsuchikama et al., 2024).

However, certain limitations are noted for the current meta-analysis. First, potential factors such as baseline characteristics and histological classifications could contribute to the observed heterogeneities. Second, our study included more non-randomized cohorts than RCT cohorts, introducing potential heterogeneities between the two, despite the general belief that RCTs substantially mitigate confounding factors. Third, although prospective studies provide stronger evidence, their stringent patient selection criteria could restrict enrollment of early-stage UC patients undergoing ADC treatments. Consequently, the benefits for these patients remain uncertain compared to those for the gold standard of care. Moreover, given the limited availability of research, the extent of discrepancies among ADCs remains unclear, highlighting the need for increased attention to a broader range of ADCs.

## 5 Conclusion

In summary, the results of the current meta-analysis indicate that ADCs exhibit modest clinical response rates accompanied by acceptable survival rates and occurrences of fatal TRAEs in patients with advanced or metastatic UCs, providing evidence for the future research and clinical applications of such ADCs. Among the ADCs available today, EV stands out as the priority treatment and demonstrates favorable prospects.

## Data Availability

The original contributions presented in the study are included in the article/Supplementary Material; further inquiries can be directed to the corresponding authors.
