# Supplementary material for: Efficacy and safety of antibody–drug conjugates in the treatment of urothelial cell carcinoma: a systematic review and meta-analysis of prospective clinical trials
Source: Front Pharmacol. 2024 Jun 12;15:1377924. doi: 10.3389/fphar.2024.1377924 (PMC11199396; doi:10.3389/fphar.2024.1377924)
Supplement: Supplementary file 1 [file DataSheet1.pdf]

ORR

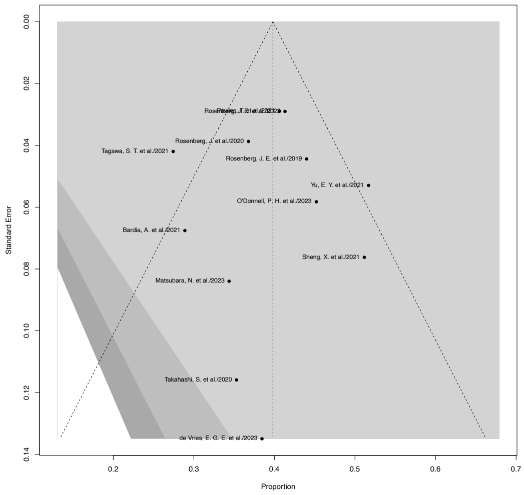

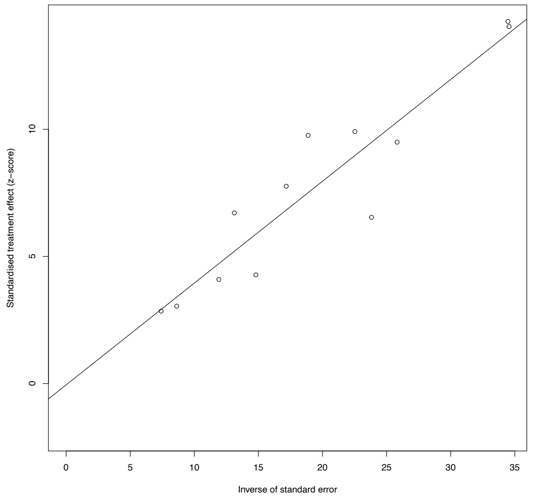

Figure S1A Funnel plot of ORR.
Figure S1B Egger test of ORR.

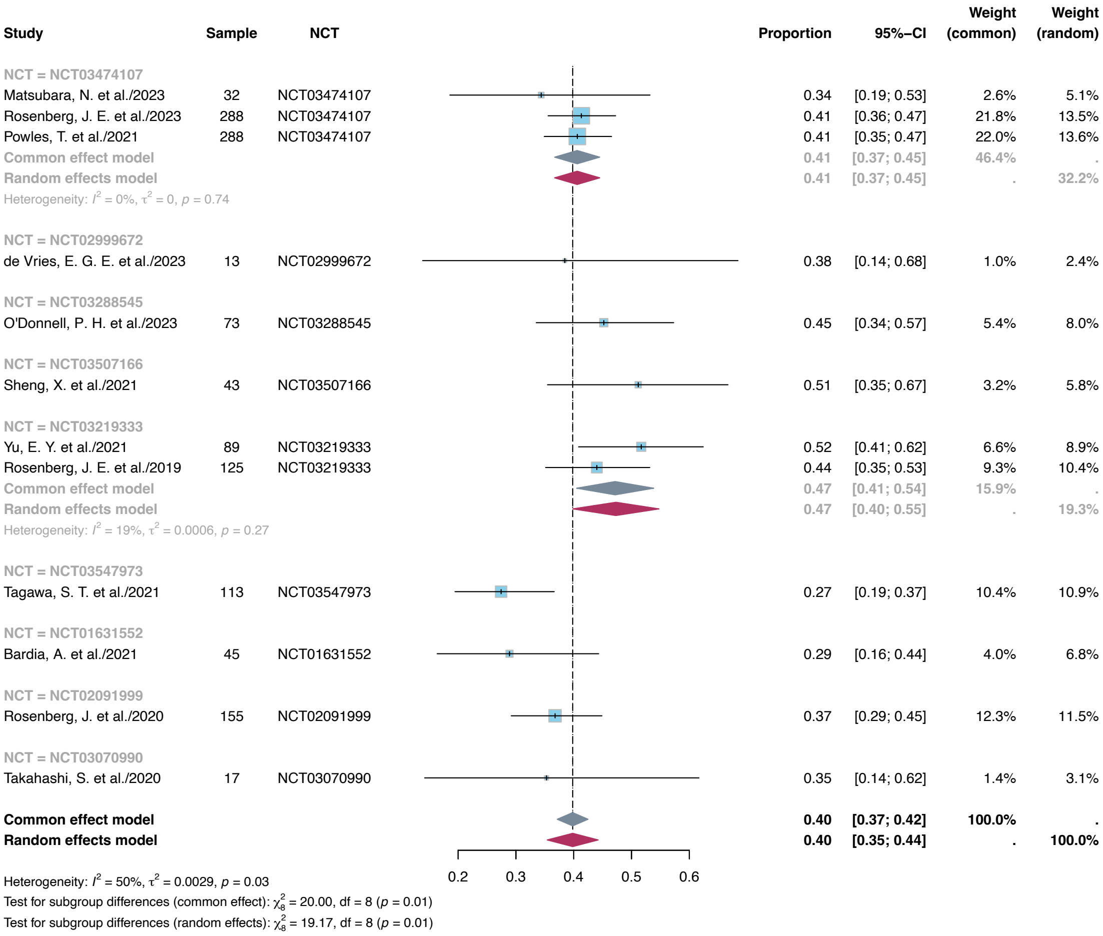

Figure S2 Forest plot of subgroup analysis of ORR based on trial identifier.

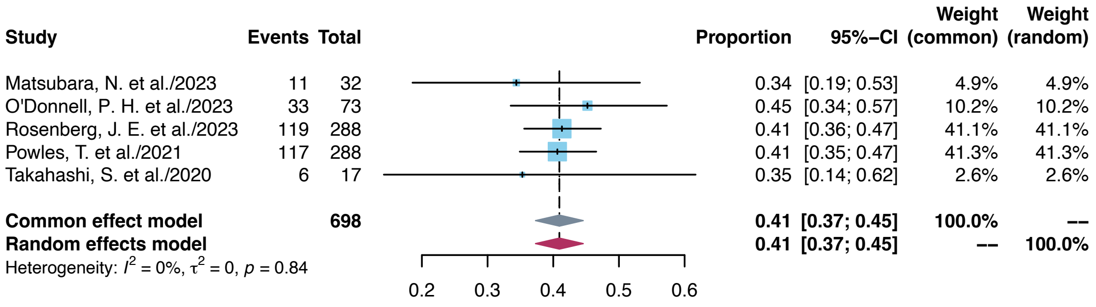

Figure S3 Forest plot of ORR based on RCTs.

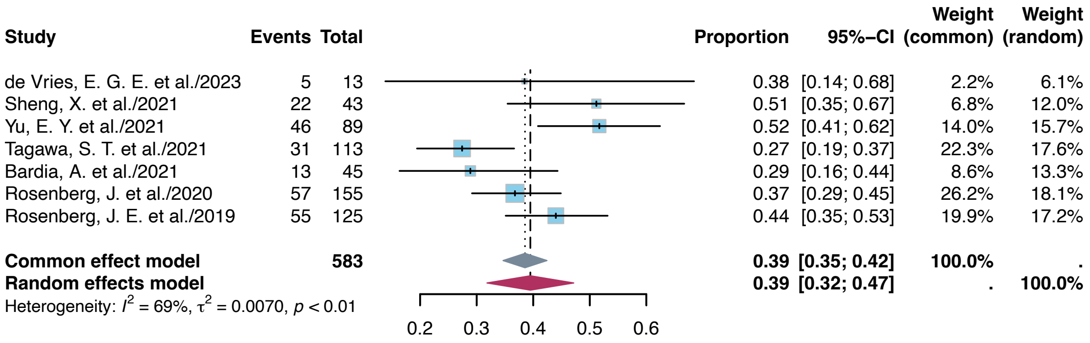

Figure S4 Forest plot of ORR based on non-RCTs.

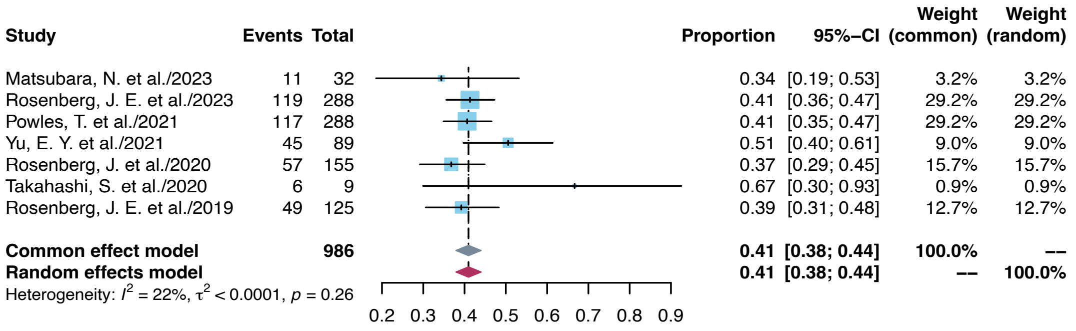

Figure S5 Forest plot of BICR group in EV cohorts.

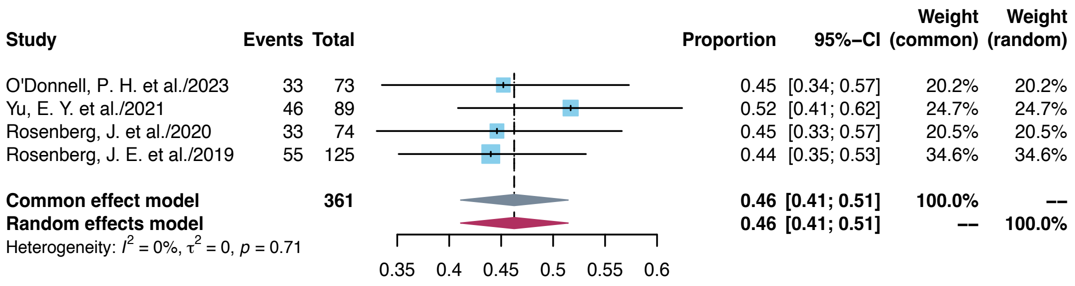

Figure S6 Forest plot of IA group in EV cohorts.

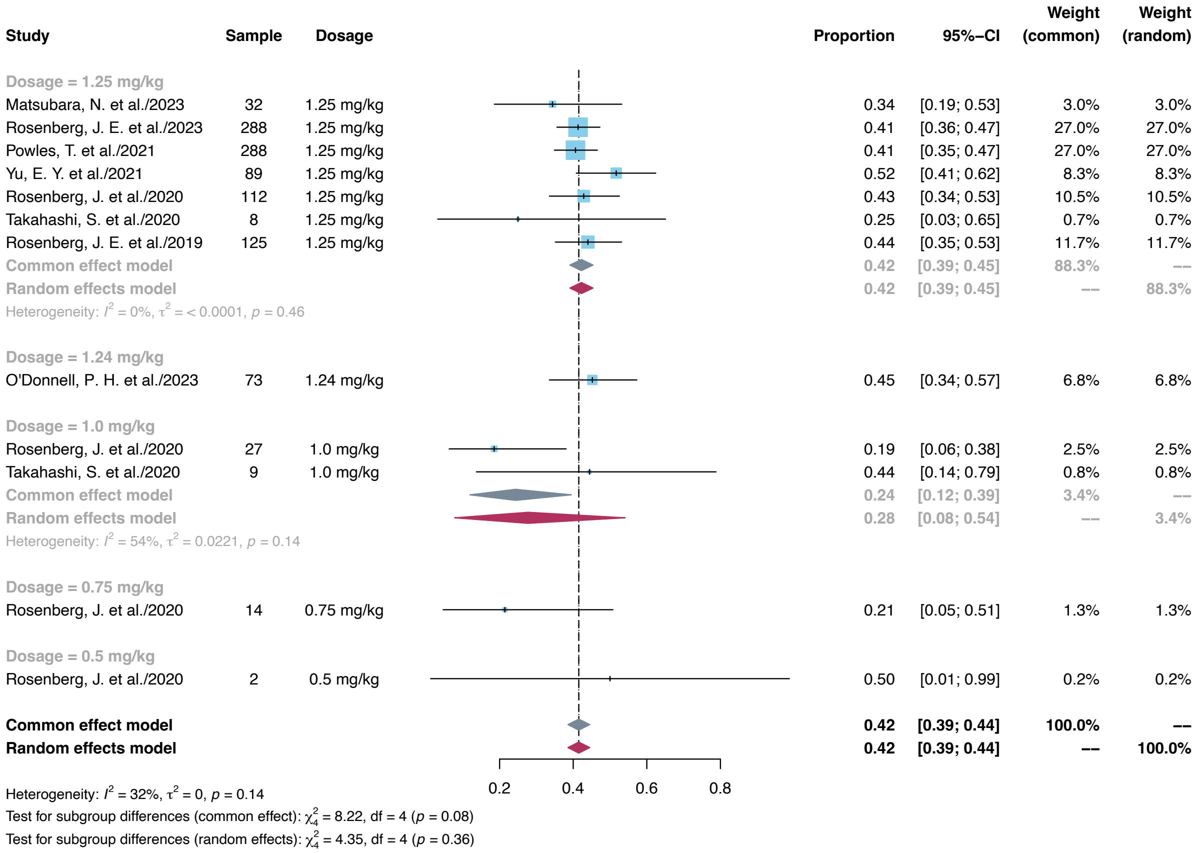

Figure S7 Forest plot of dosage analysis in EV cohorts.

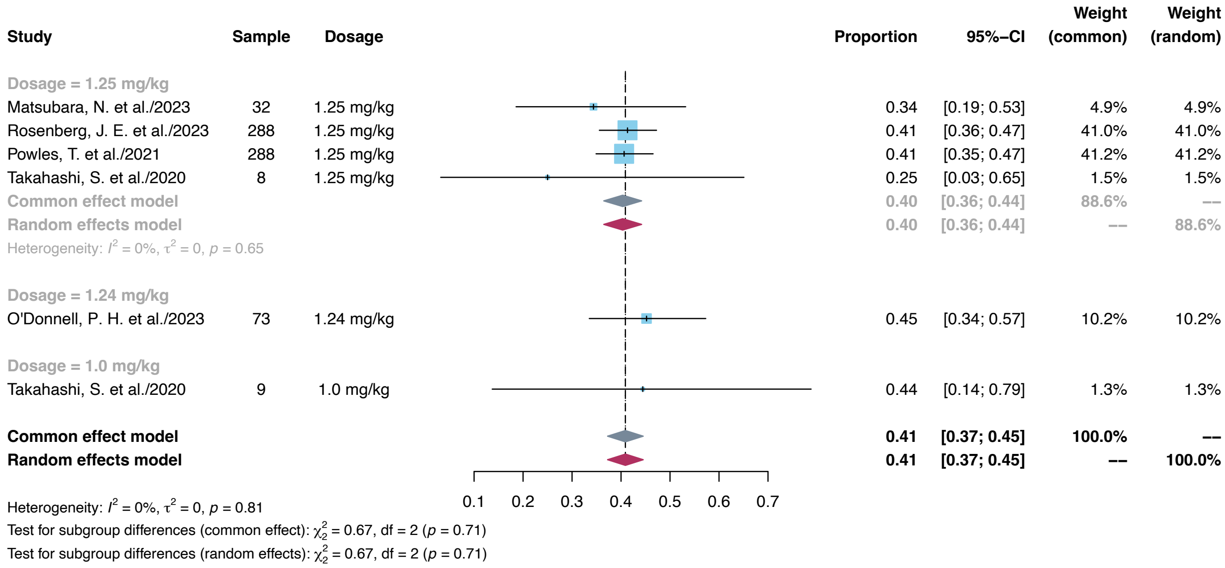

Figure S8 Forest plot of dosage analysis in RCTs group.

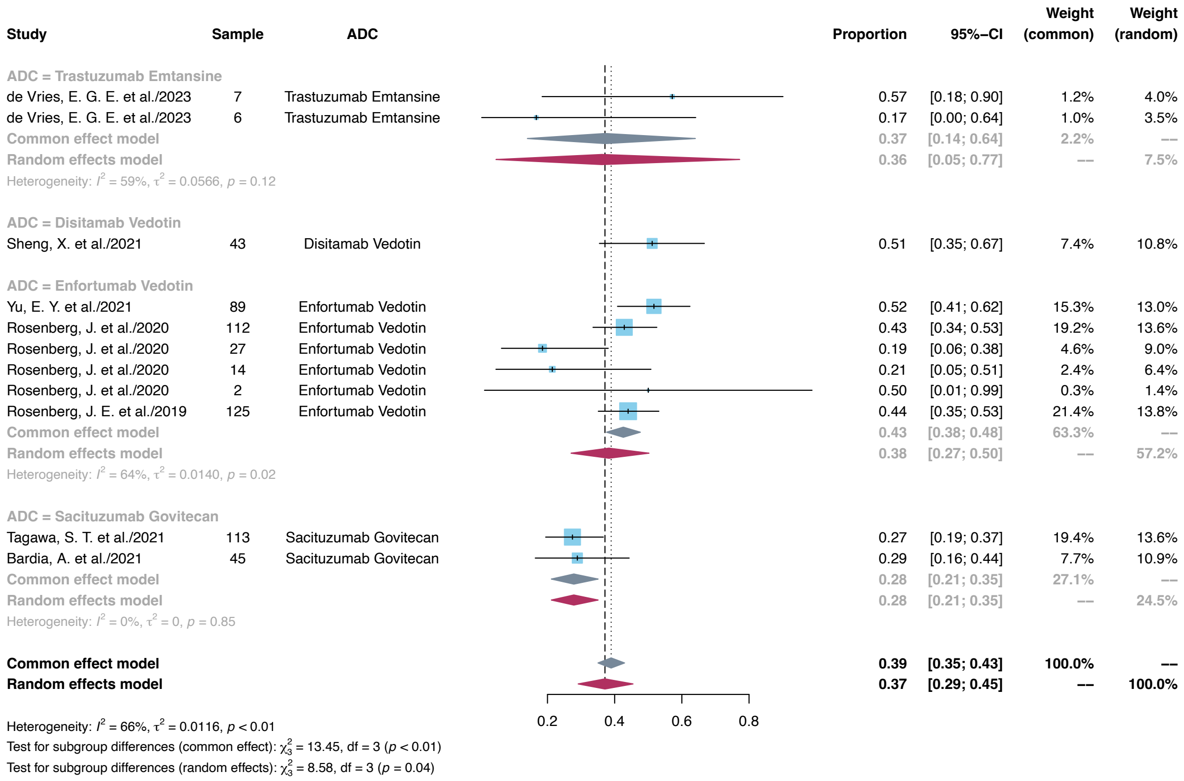

Figure S9 Forest plot of subgroup analysis of ORR based on ADC classes in non-RCTs group.

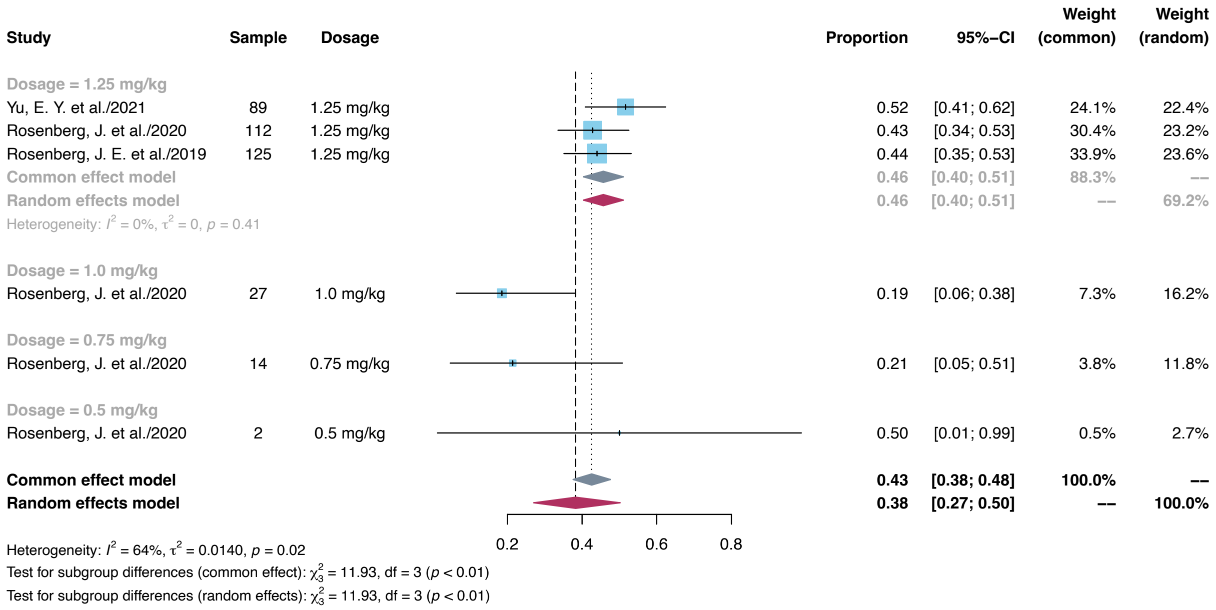

Figure S10 Forest plot of dosage analysis of EV cohorts in non-RCTs group.

DCR

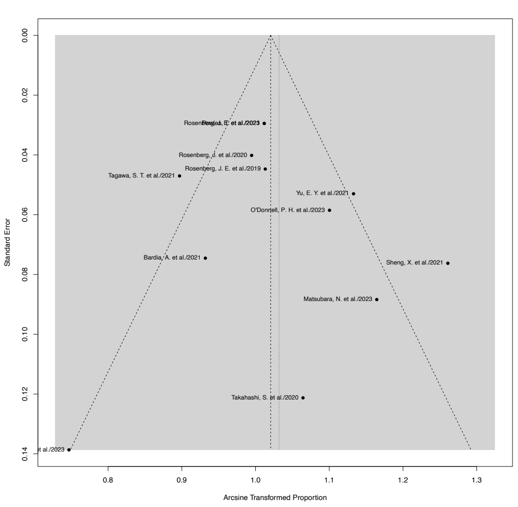

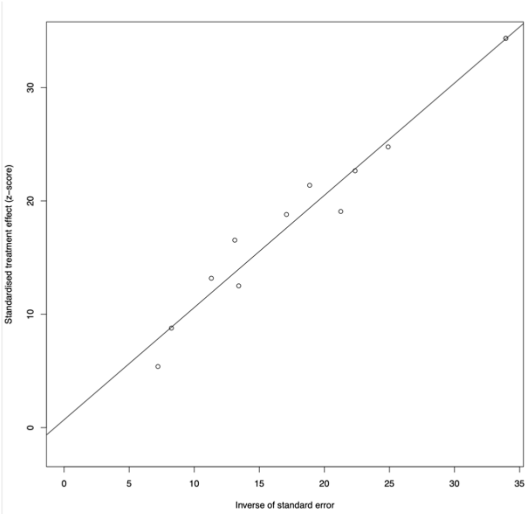

Figure S11A Funnel plot of DCR.
Figure S11B Egger test of DCR.

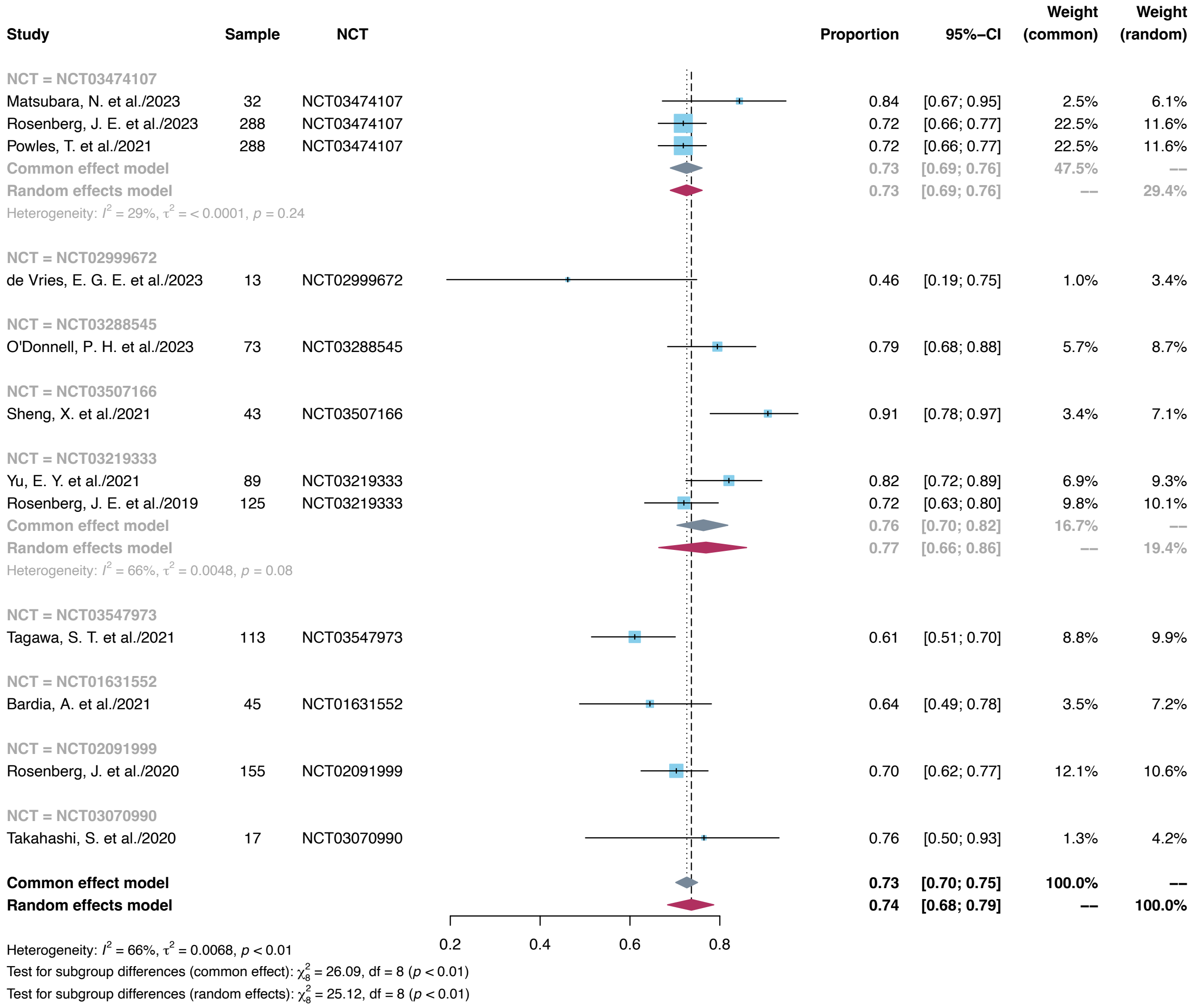

Figure S12 Forest plot of subgroup analysis of DCR based on trial identifier.

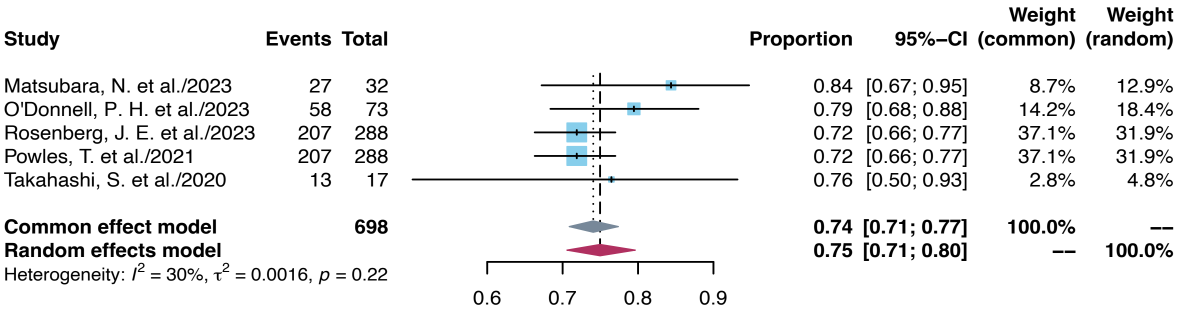

Figure S13 Forest plot of DCR based on RCTs.

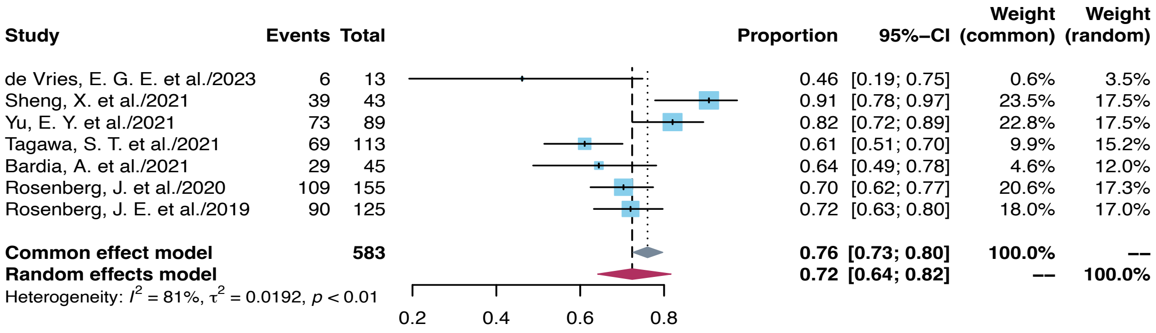

Figure S14 Forest plot of DCR based on non-RCTs.

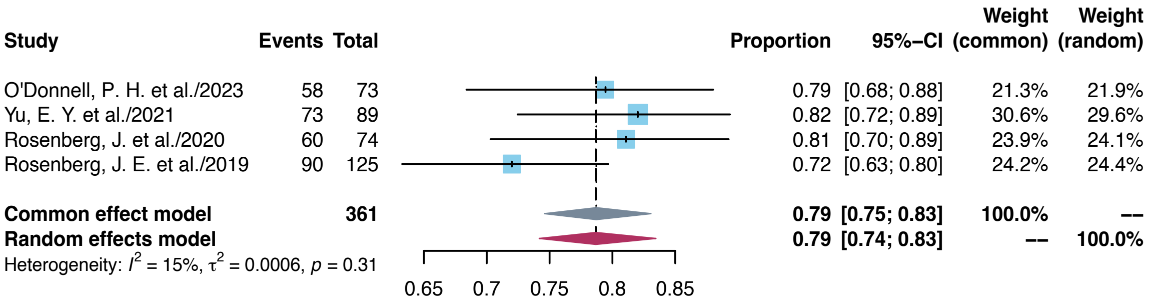

Figure S15 Forest plot of IA group in EV cohorts.

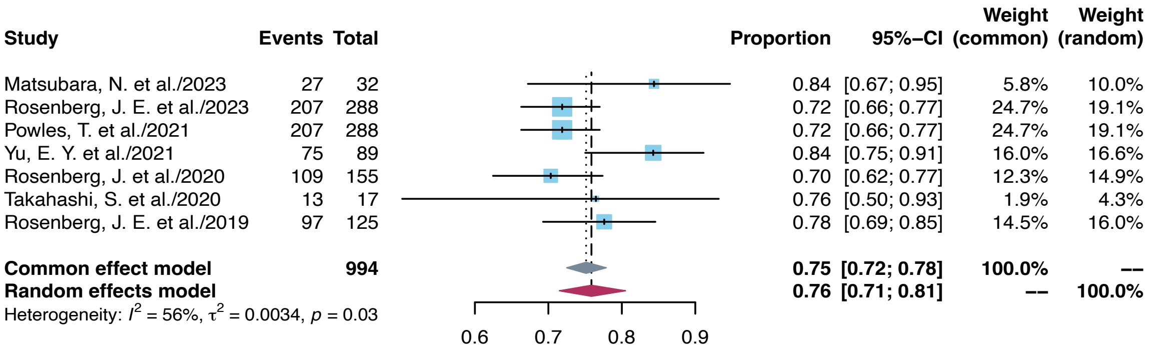

Figure S16 Forest plot of BICR group in EV cohorts.

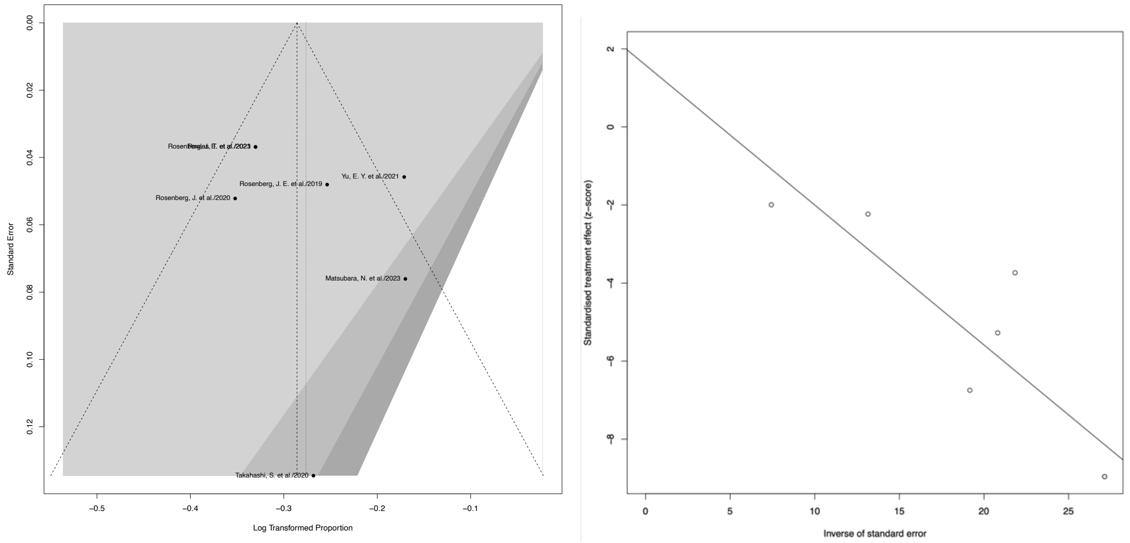

Figure S17A Funnel plot of IA group.
Figure S17B Egger tests of IA group.

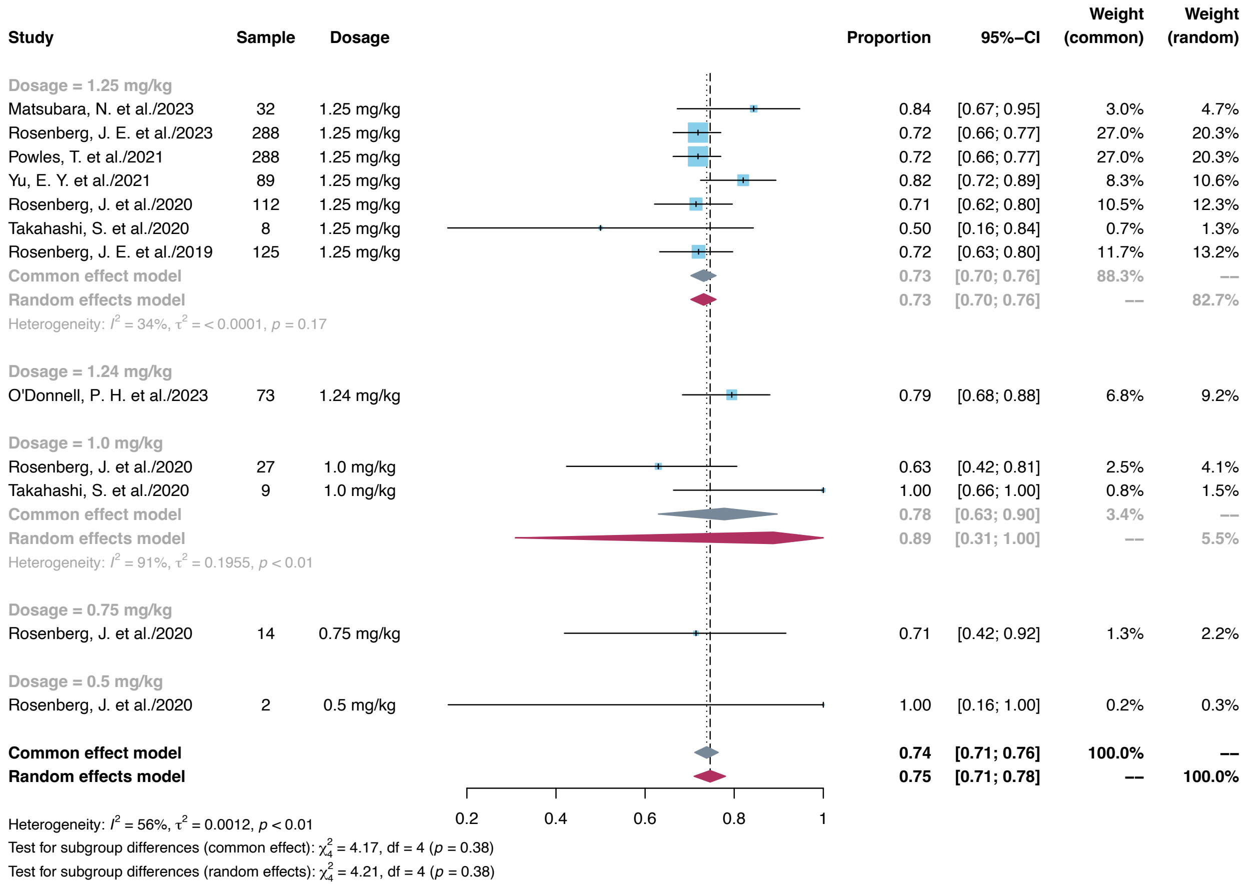

Figure S18 Forest plot of dosage analysis in EV cohorts.

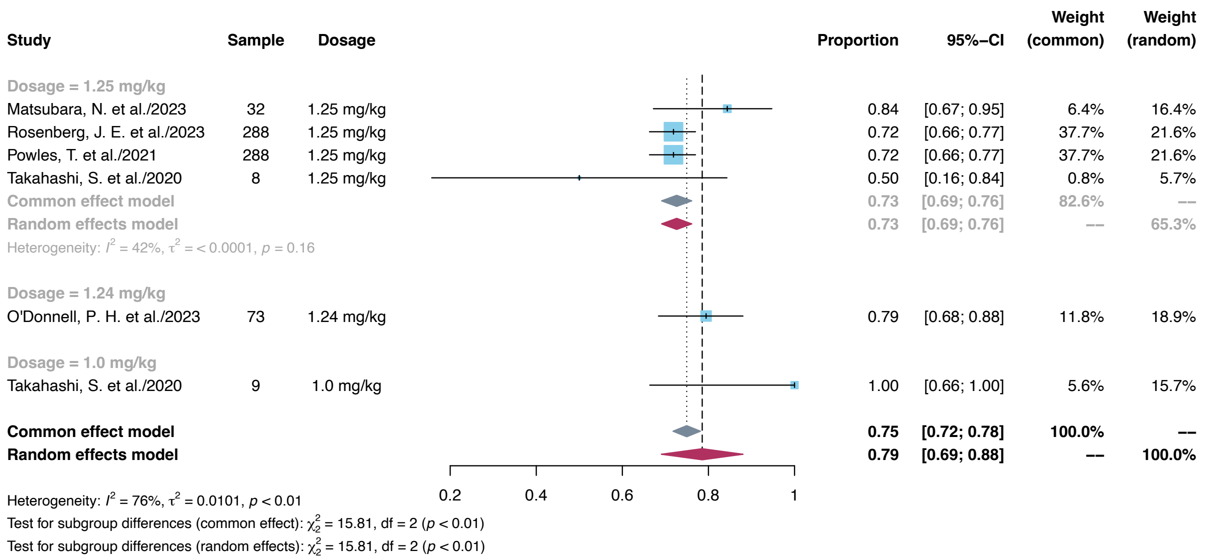

Figure S19 Forest plot of dosage analysis in RCTs group.

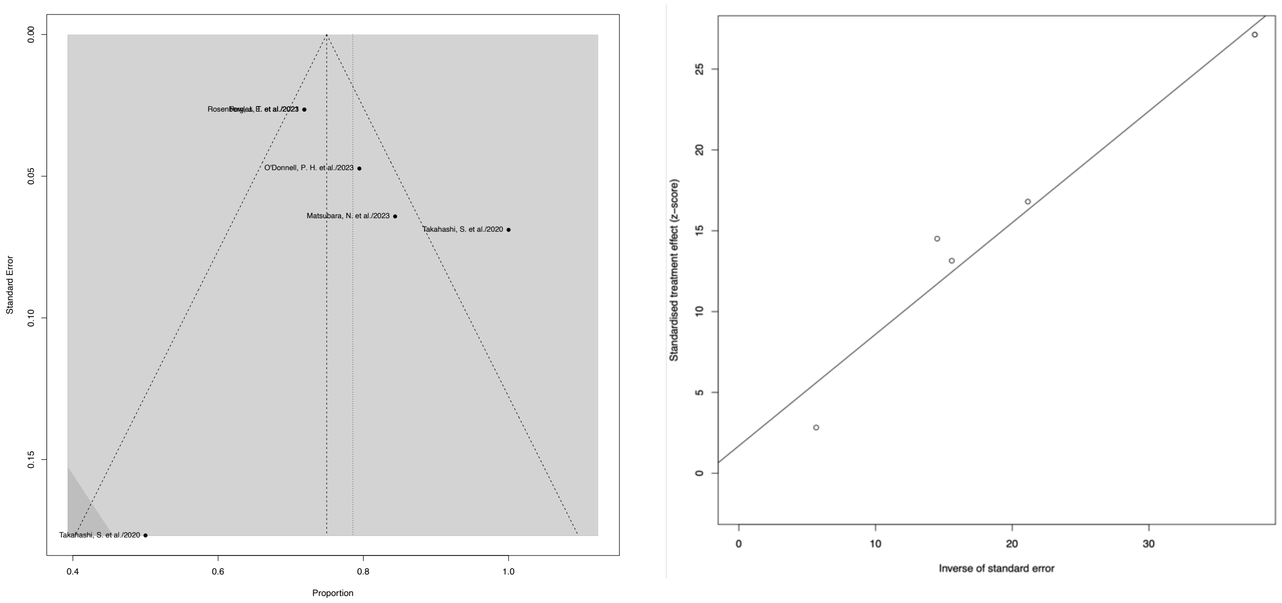

Figure S20A Funnel plot of DCR in RCT group.

Figure S20B Egger test of DCR in RCT group.

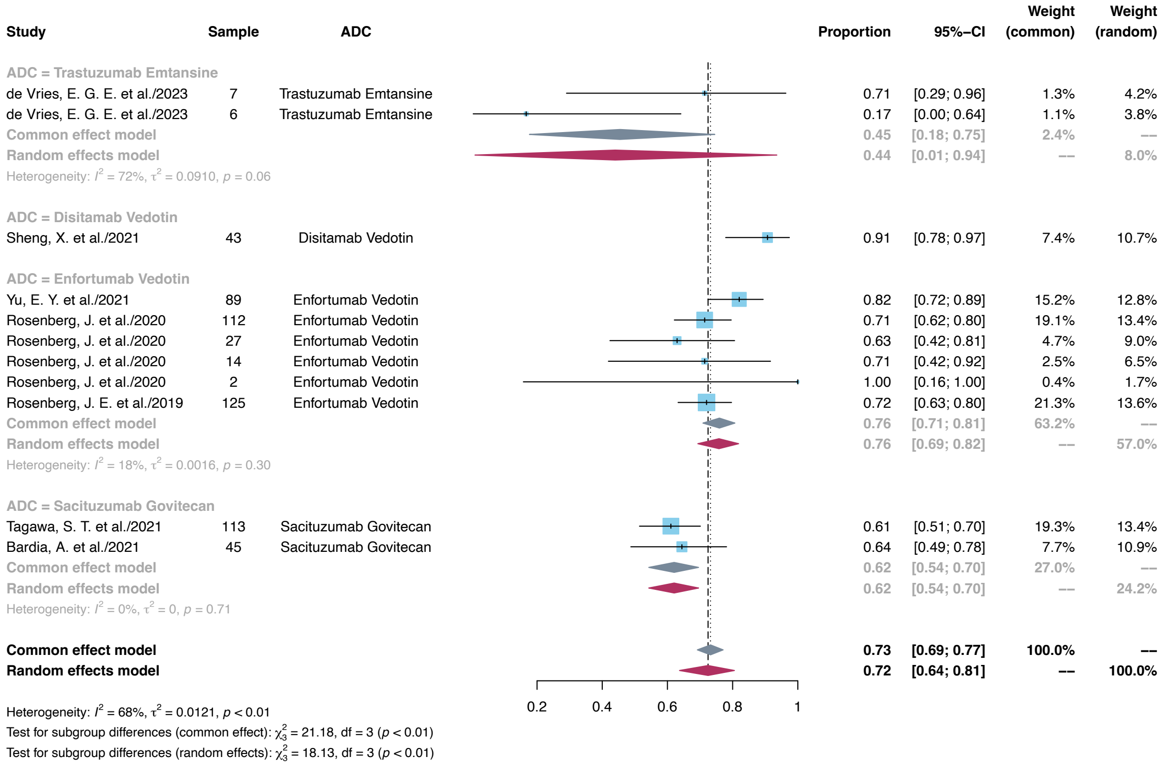

Figure S21 Forest plot of subgroup analysis of DCR based on ADC classes in non-RCTs group.

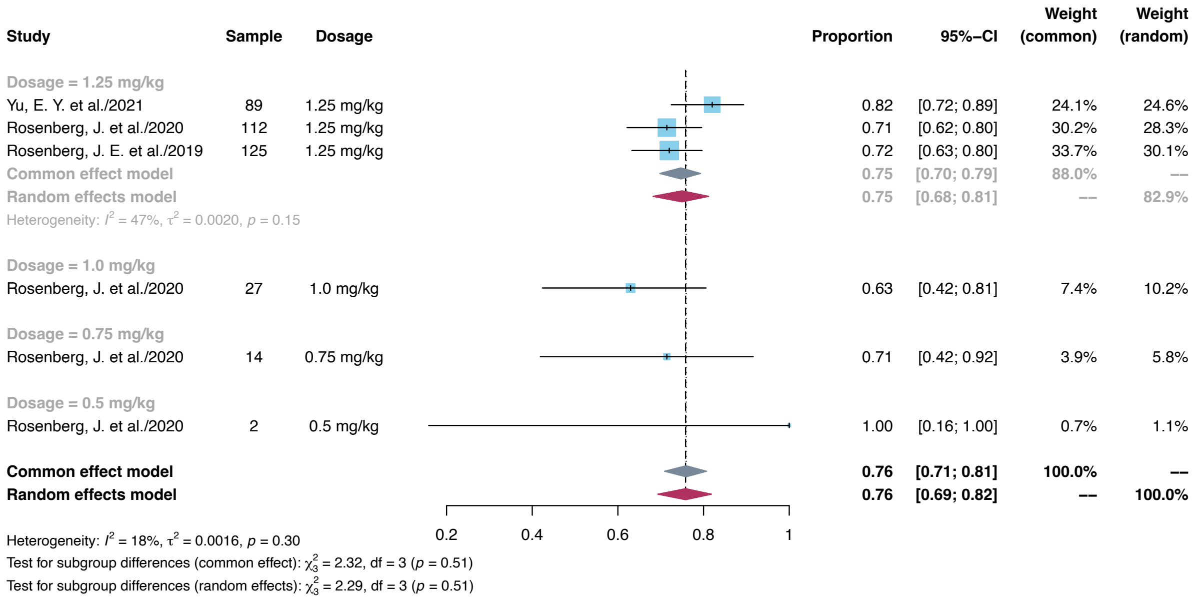

Figure S22 Forest plot of dosage analysis of EV cohorts in non-RCTs group.

PFS

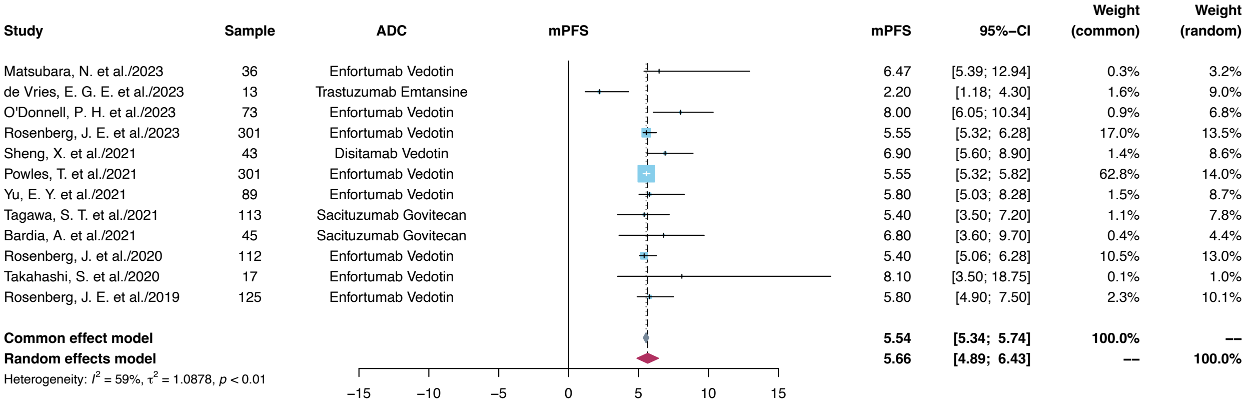

Figure S23 Forest plot of median PFS.

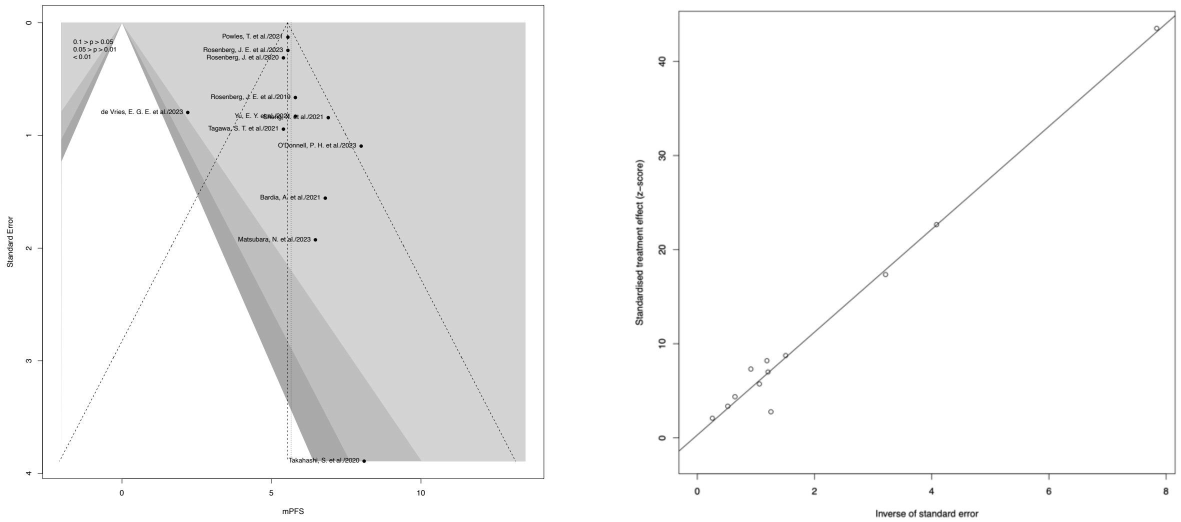

Figure S24A Funnel plot of median PFS.
Figure S24B Egger test of median PFS.

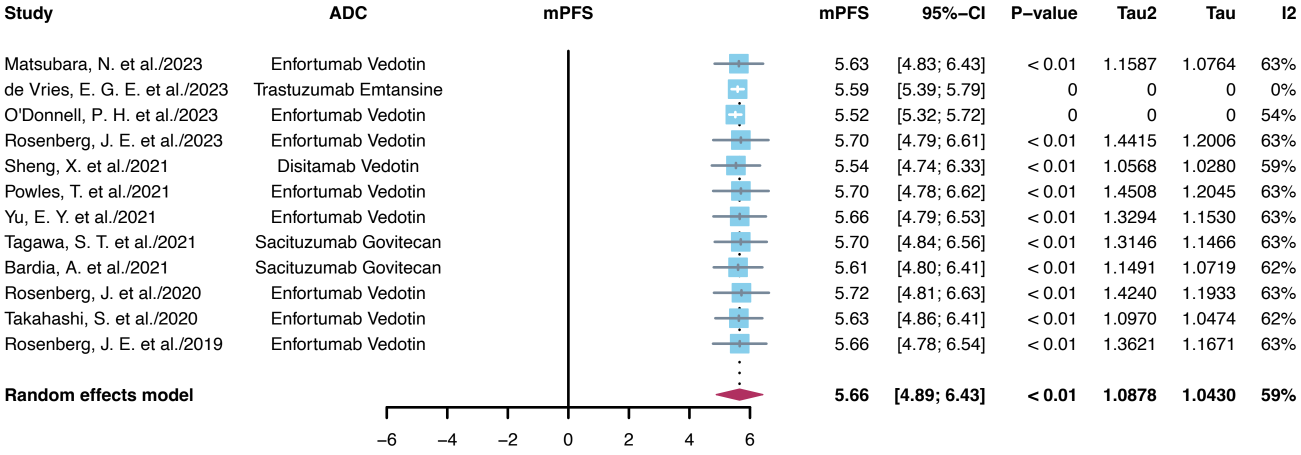

Figure S25 Sensitivity analysis of median PFS.

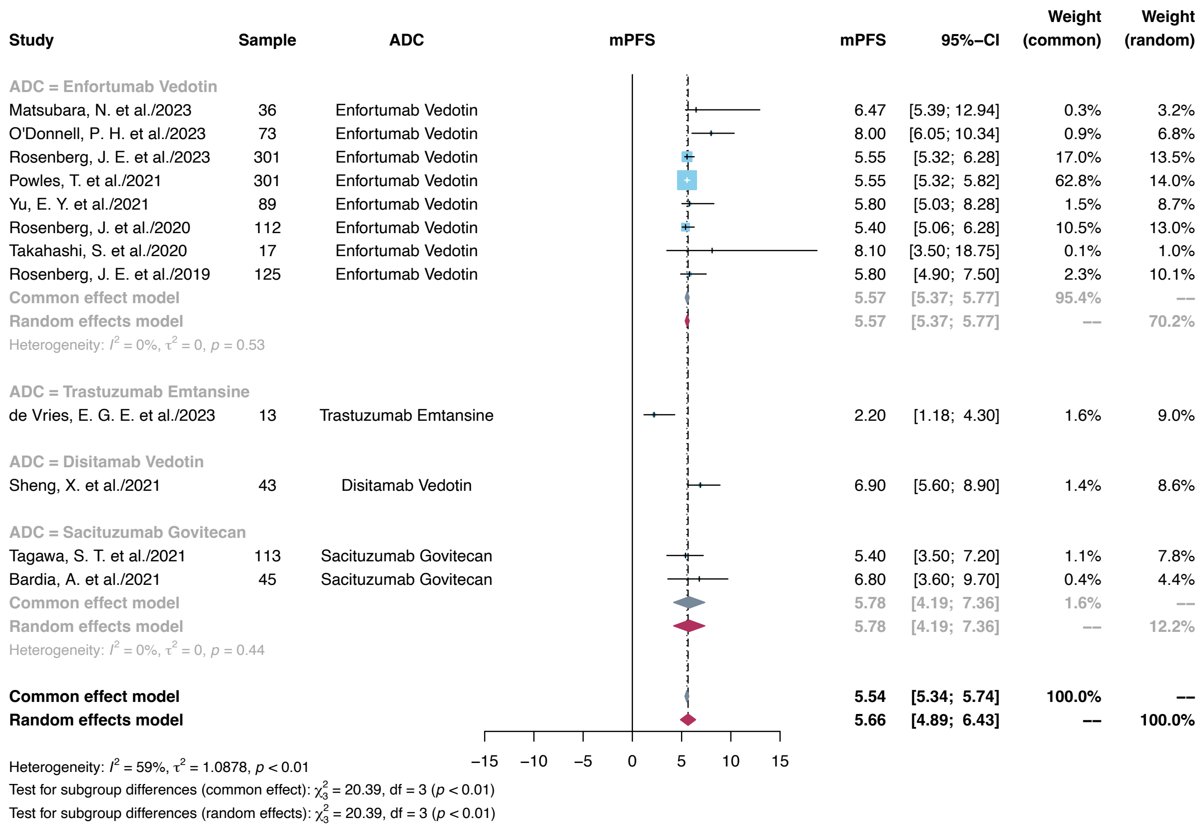

Figure S26 Forest plot of subgroup analysis of median PFS based on ADC classes.

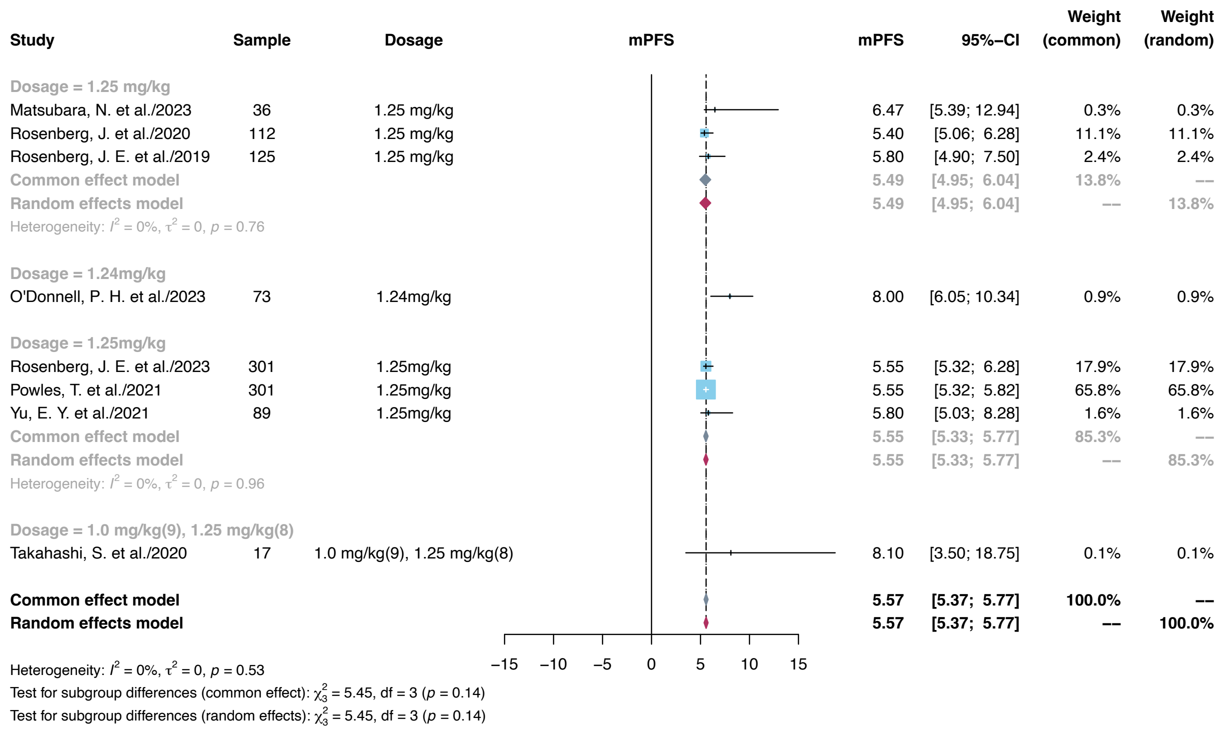

Figure S27 Forest plot of dosage analysis of median PFS in EV cohorts.

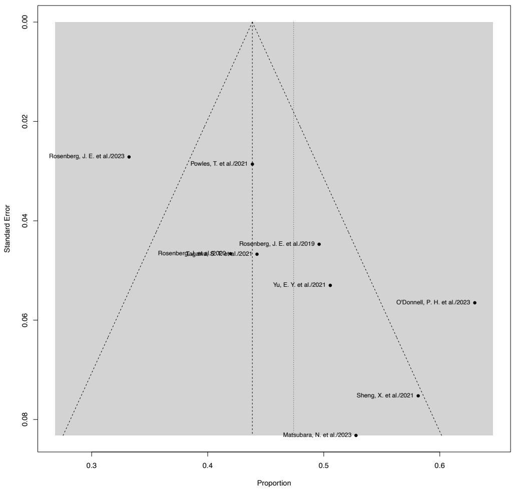

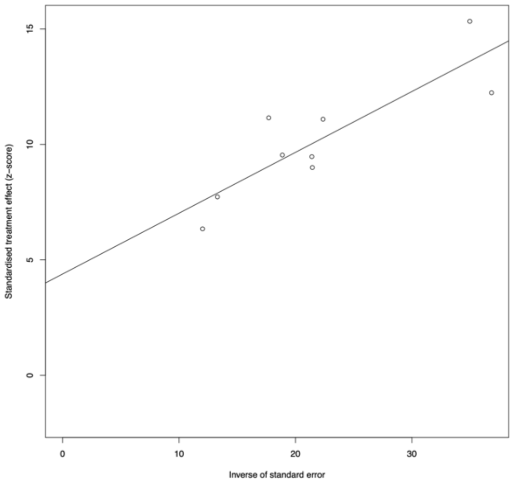

Figure S28A Funnel plot of 6-month PFS.

Figure S28B Egger test of 6-month PFS.

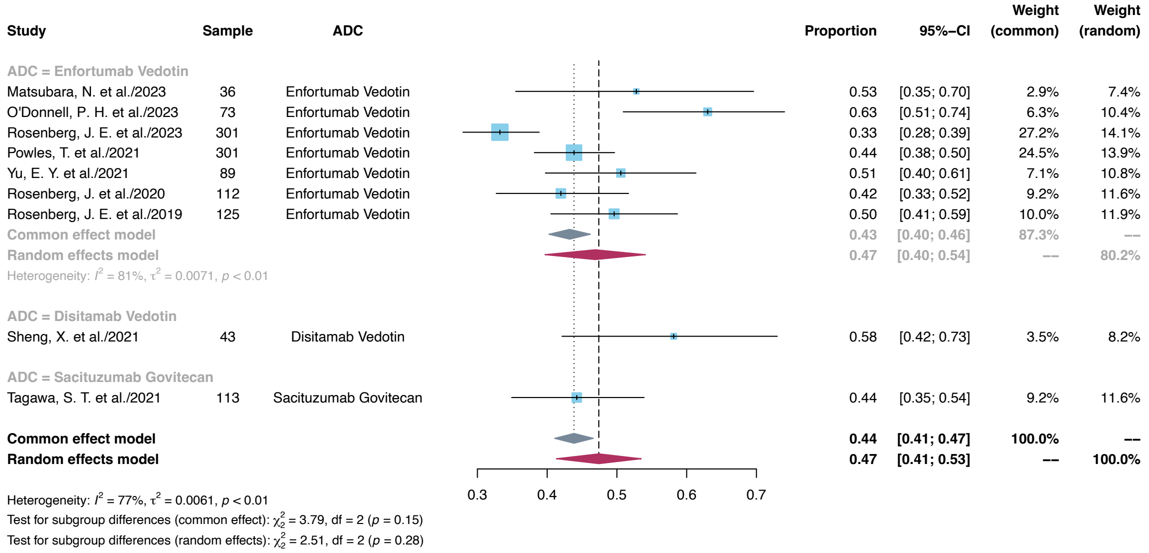

Figure S29 Forest plot of 6-month PFS analysis based on ADC classes.

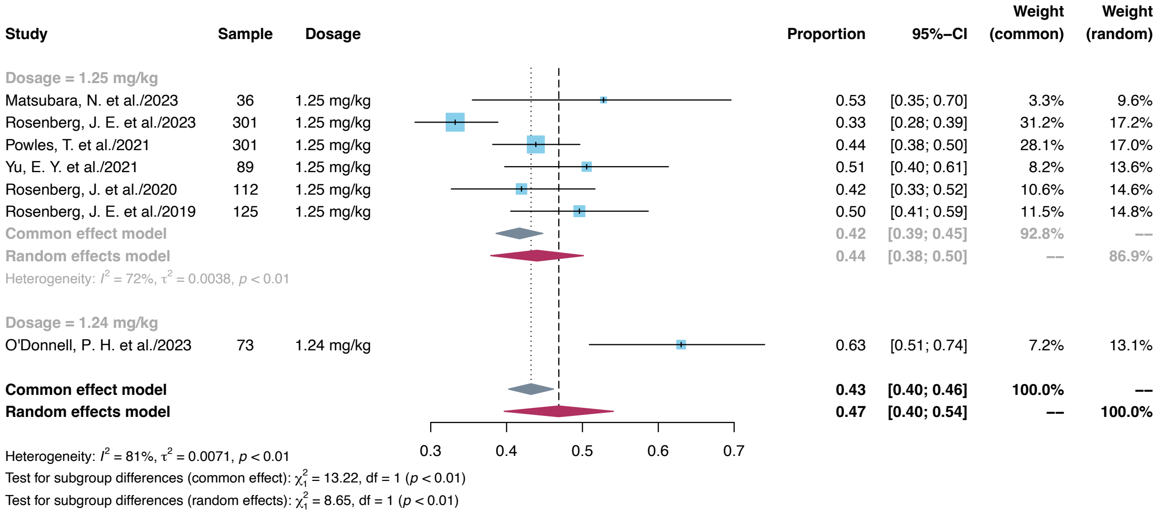

Figure S30 Forest plot of dosage analysis of 6-month PFS in EV cohorts.

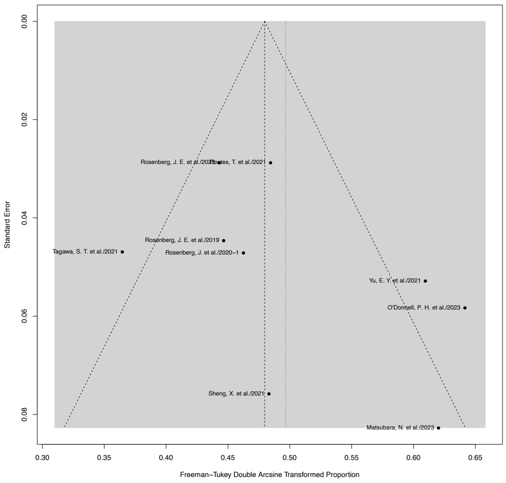

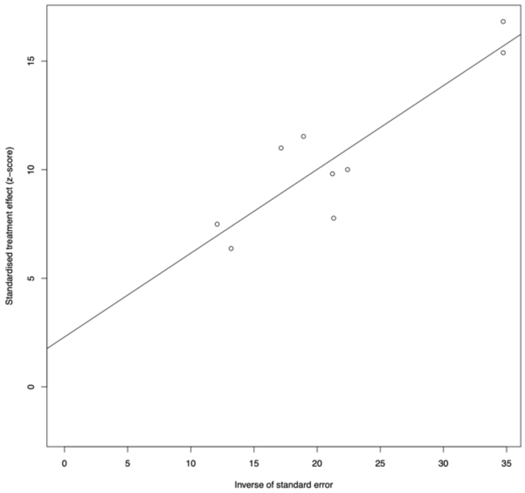

Figure S31A Funnel plot of 1-year PFS.

Figure S31B Egger test of 1-year PFS.

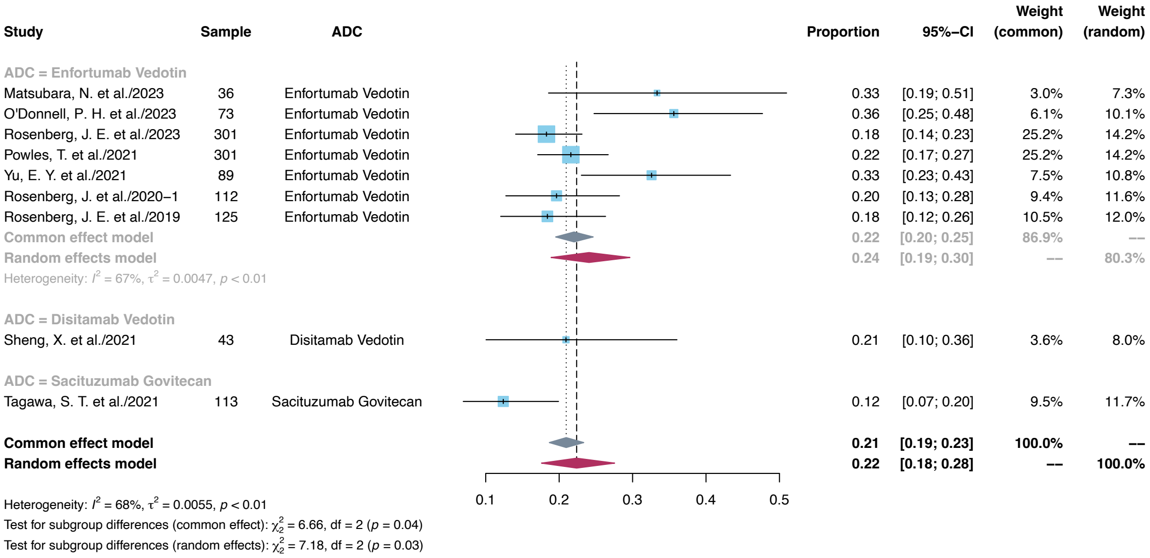

Figure S32 Forest plot of 1-year PFS analysis based on ADC classes.

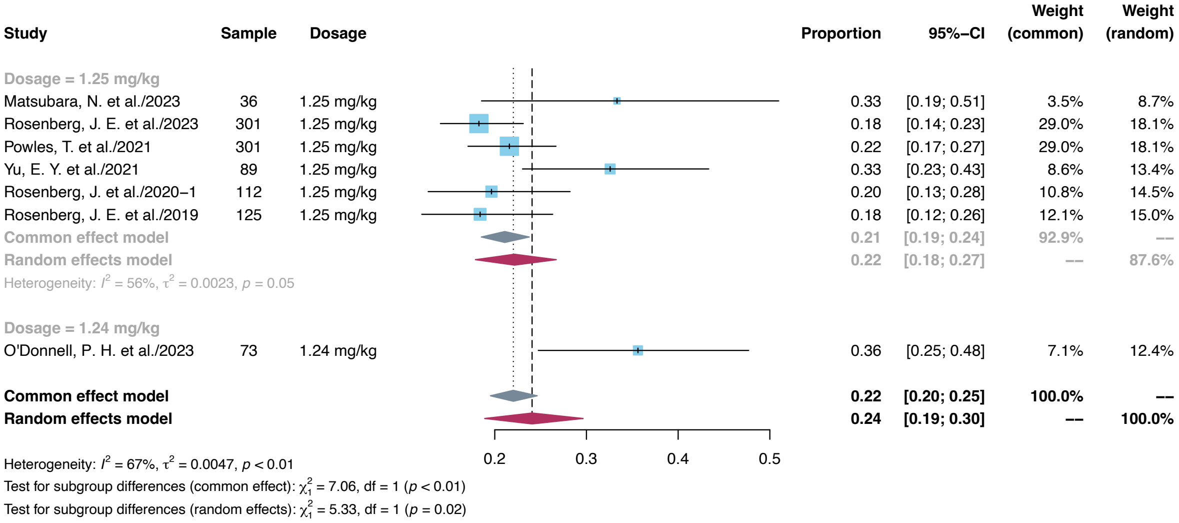

Figure S33 Forest plot of dosage analysis of 1-year PFS in EV cohorts.

OS

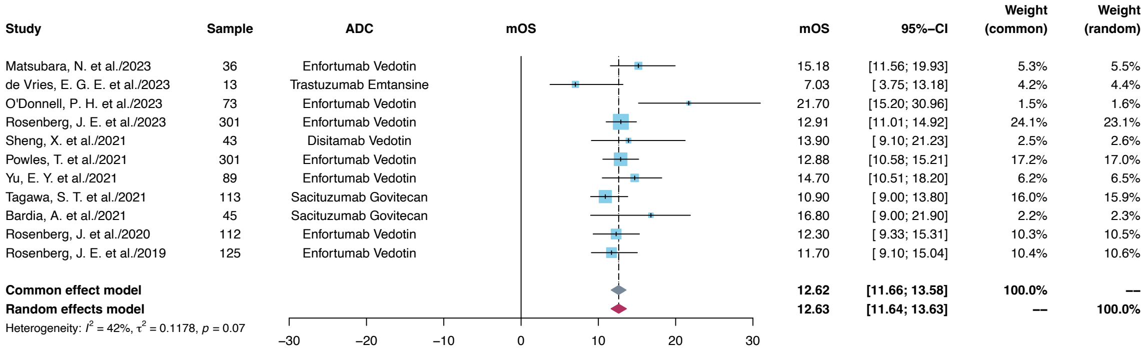

Figure S34 Forest plot of median OS.

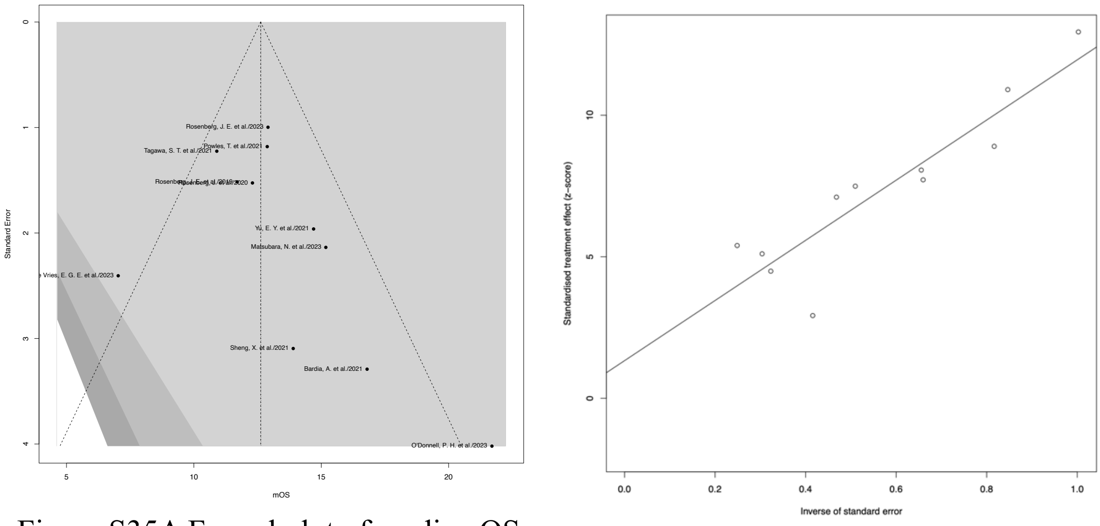

Figure S35A Funnel plot of median OS.

Figure S35B Egger test of median OS.

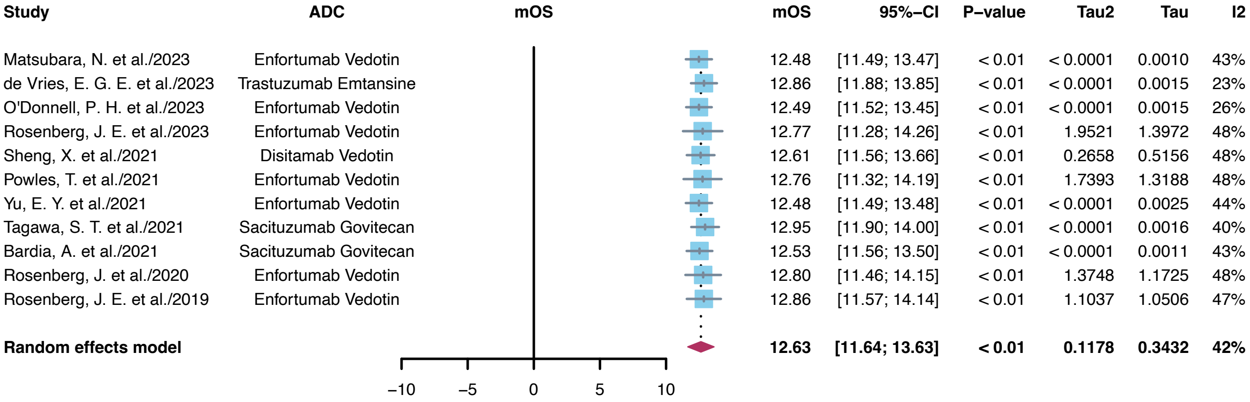

Figure S36 Sensitivity analysis of median OS.

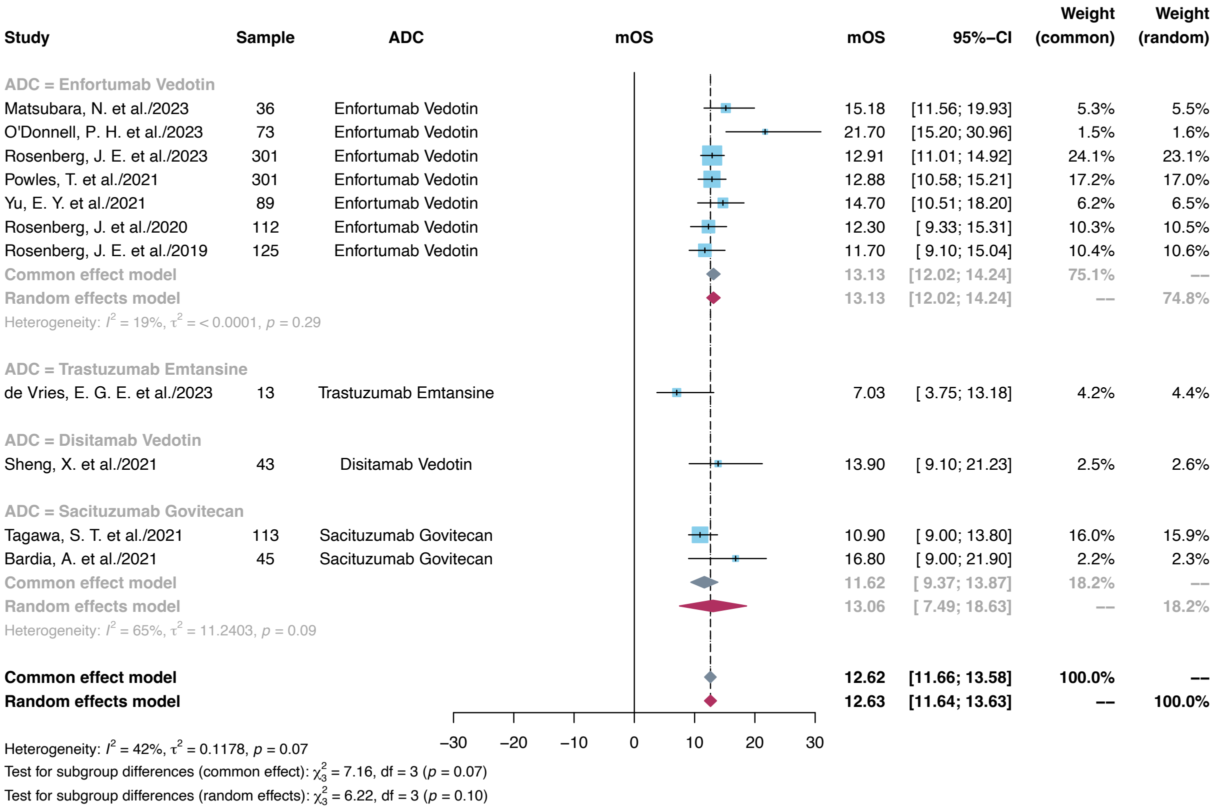

Figure S37 Forest plot of subgroup analysis of median OS based on ADC classes.

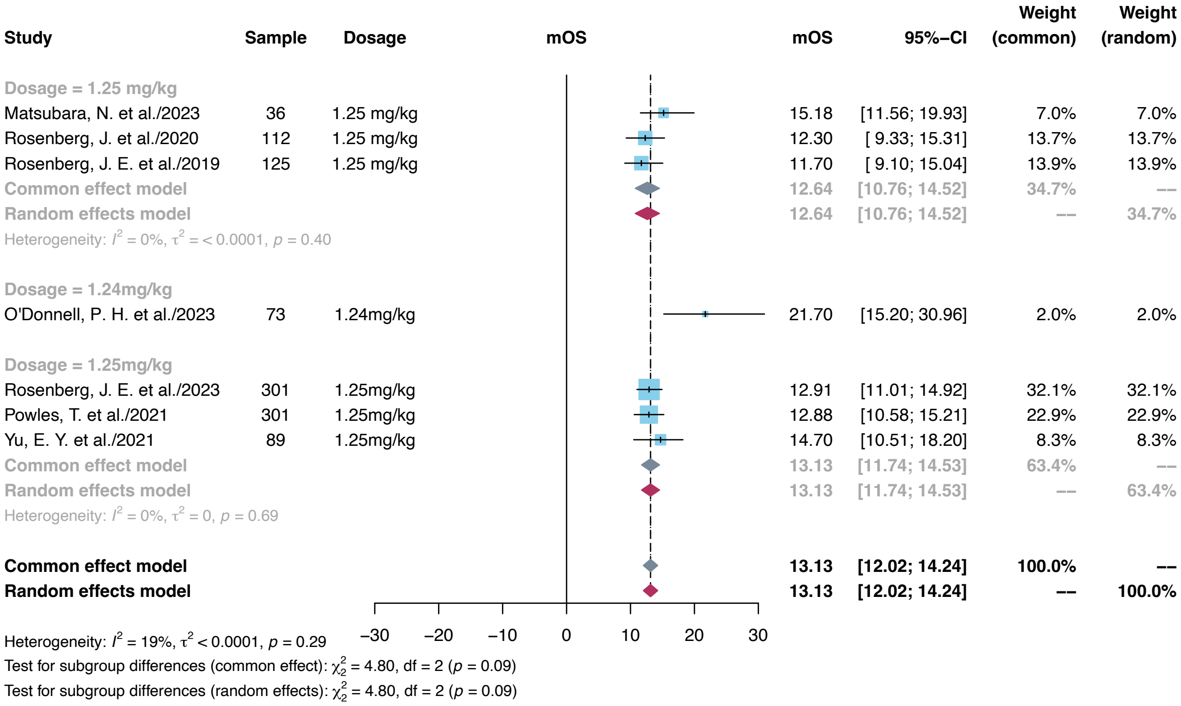

Figure S38 Forest plot of dosage analysis of median OS in EV cohorts.

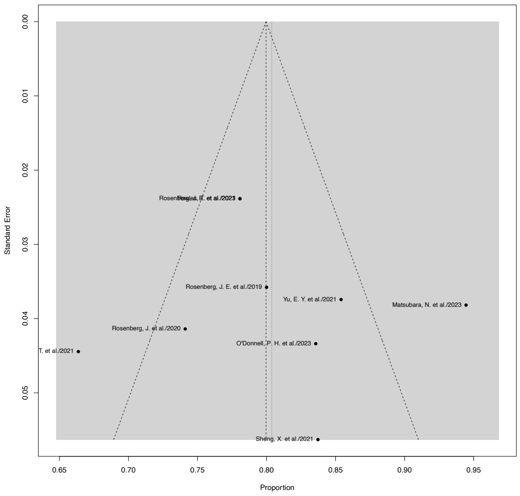

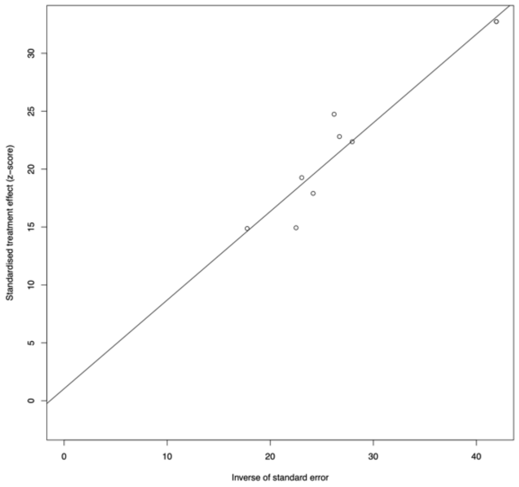

Figure S39A Funnel plot of 6-month OS.

Figure S39B Egger test of 6-month OS.

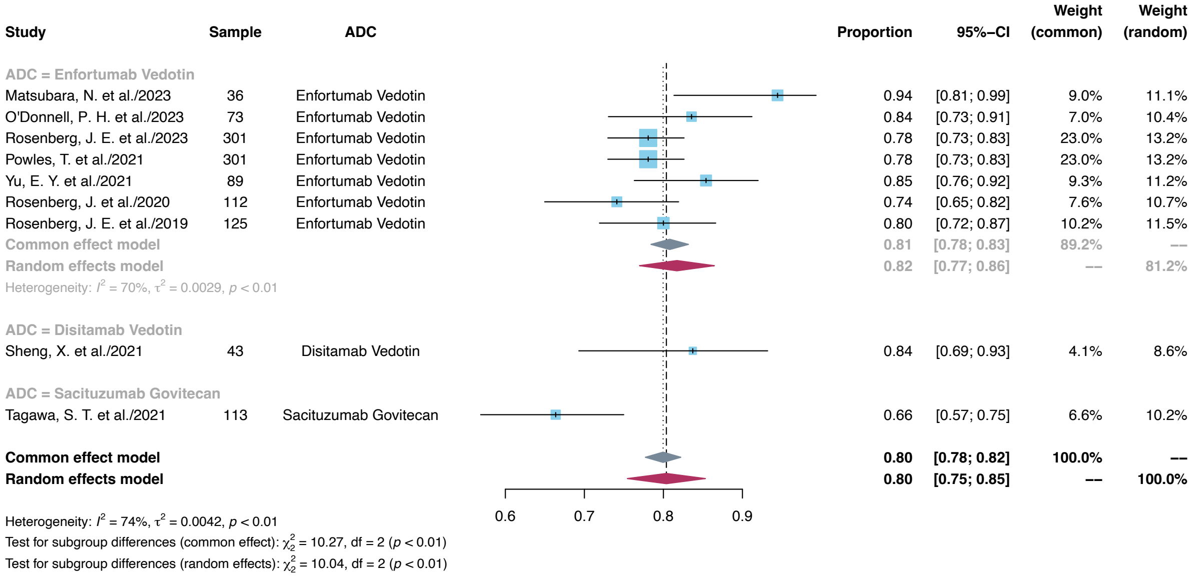

Figure S40 Forest plot of 6-month OS analysis based on ADC classes.

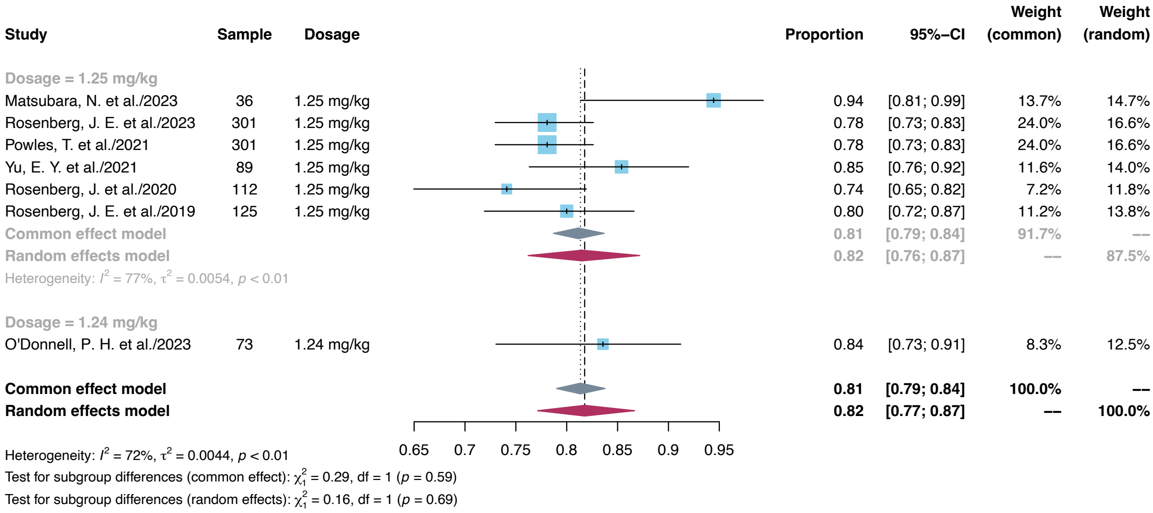

Figure S41 Forest plot of dosage analysis of 6-month OS in EV cohorts.

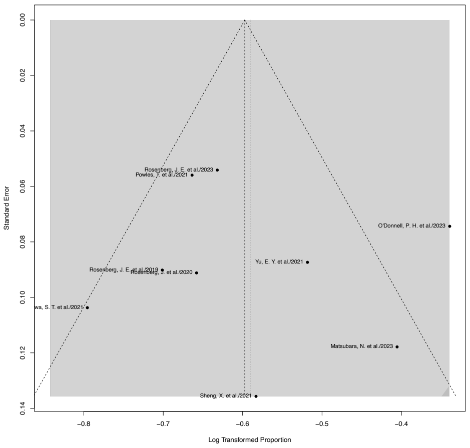

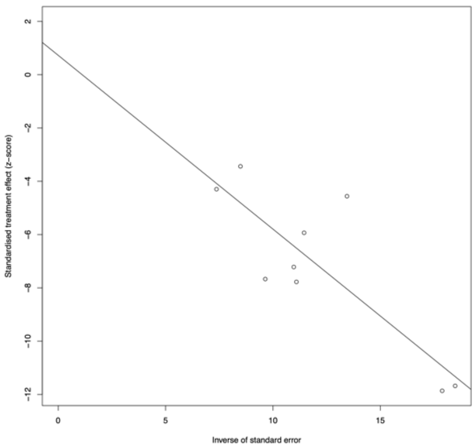

Figure S42A Funnel plot of 1-year OS

Figure S42B Egger test of 1-year OS.

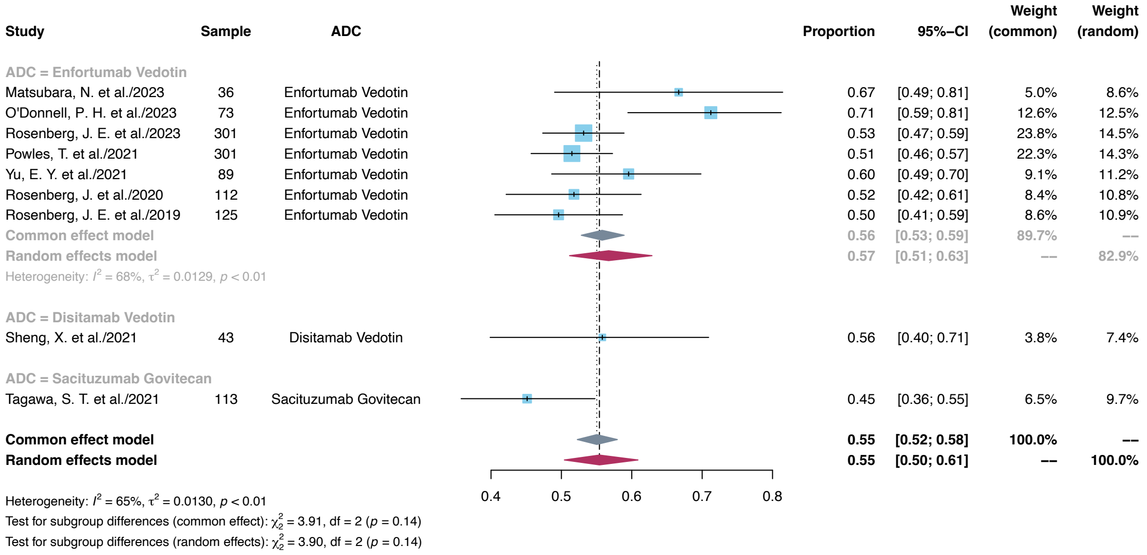

Figure S43 Forest plot of 1-year OS analysis based on ADC classes.

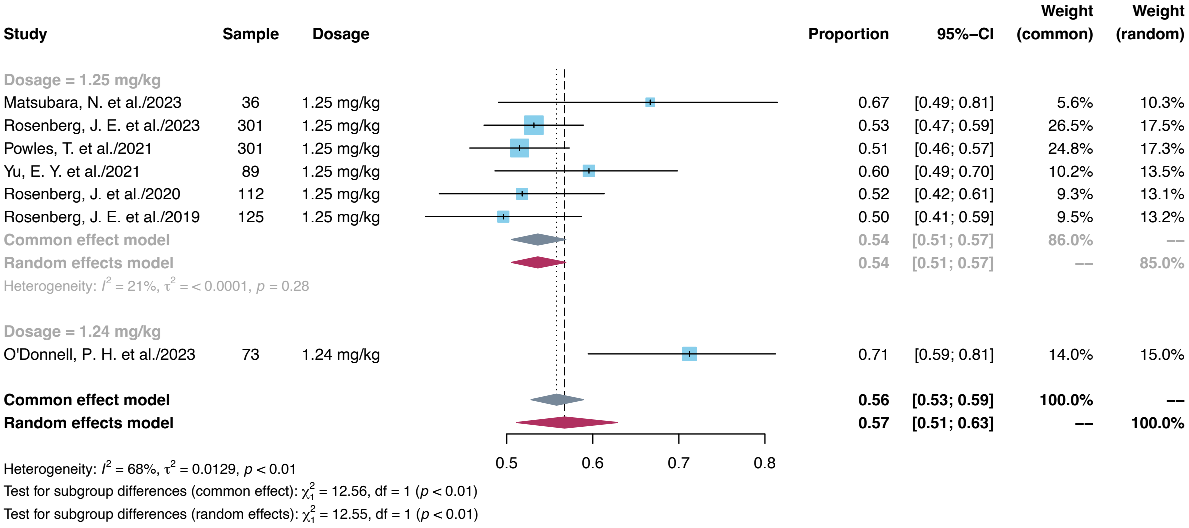

Figure S44 Forest plot of dosage analysis of 1-year OS in EV cohorts.

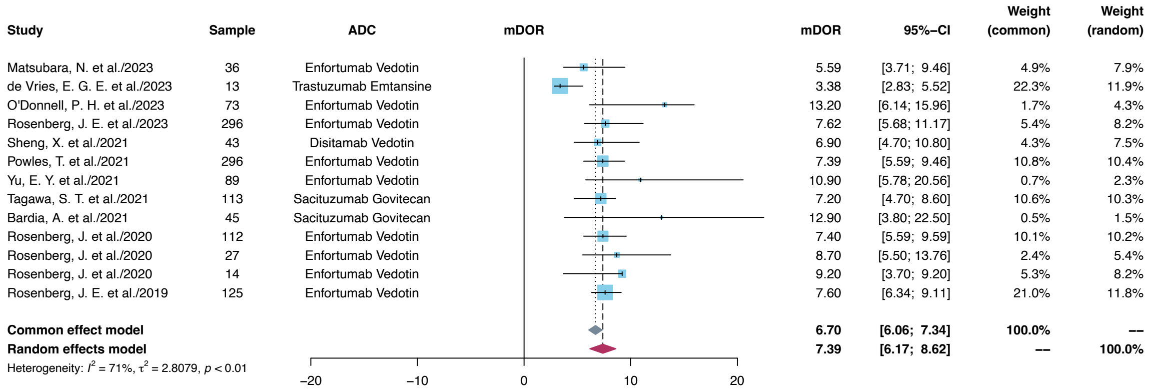

Figure S45 Forest plot of median DOR.

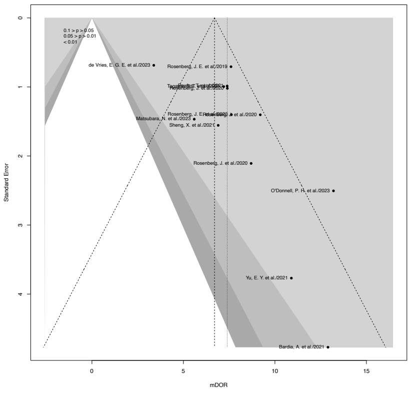

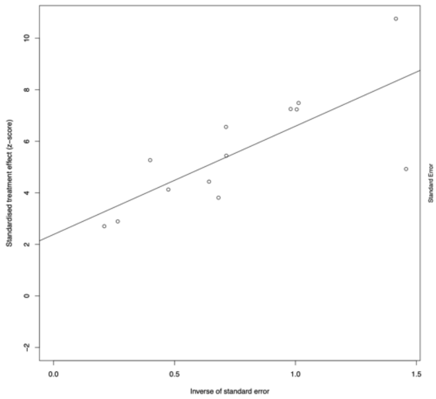

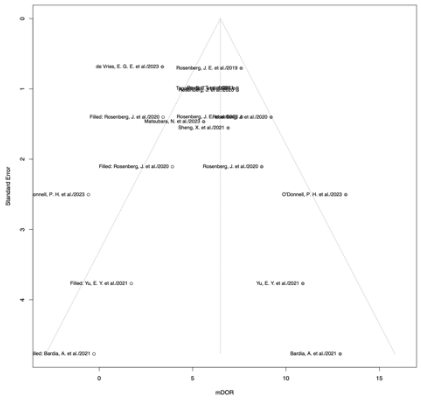

Figure S46A Funnel plot of median DOR.

Figure S46B Egger test of median DOR.

Figure S47A Funnel plot of median DOR with trim-and-fill method.

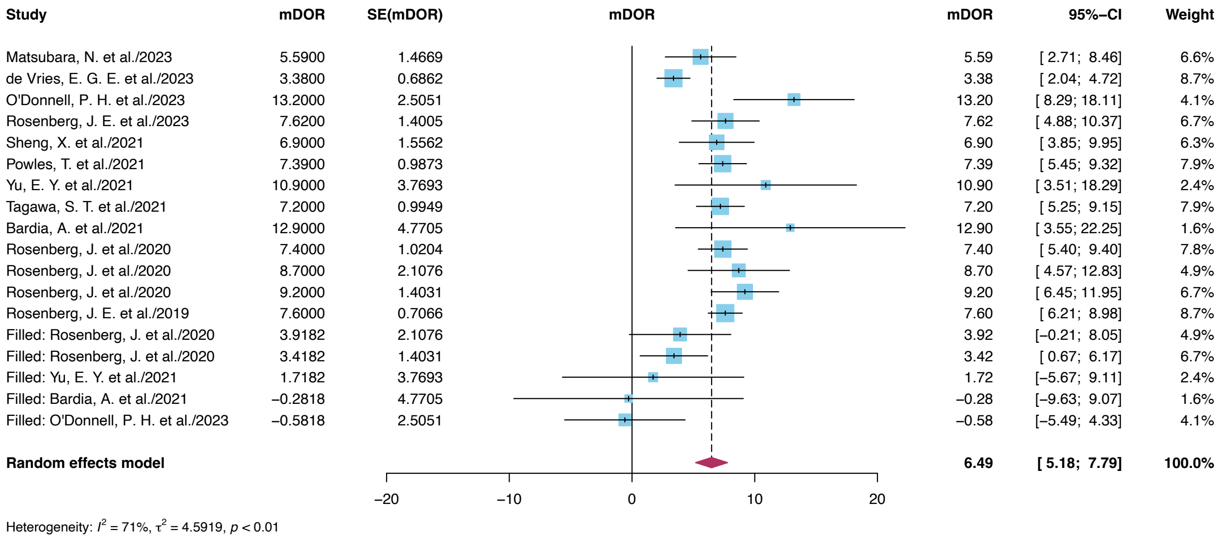

Figure S47B Forest plot of median DOR after trim-and-fill method.

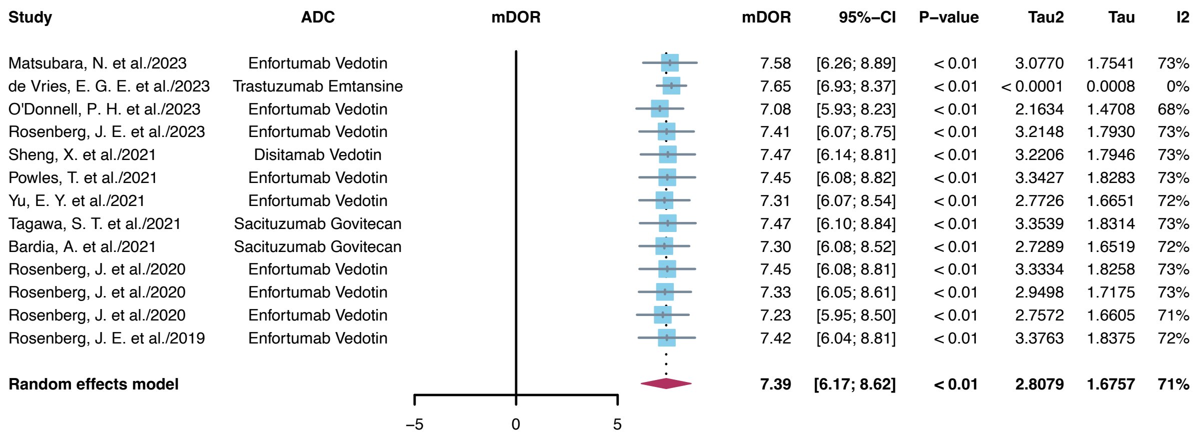

Figure S48 Sensitivity analysis of median DOR.

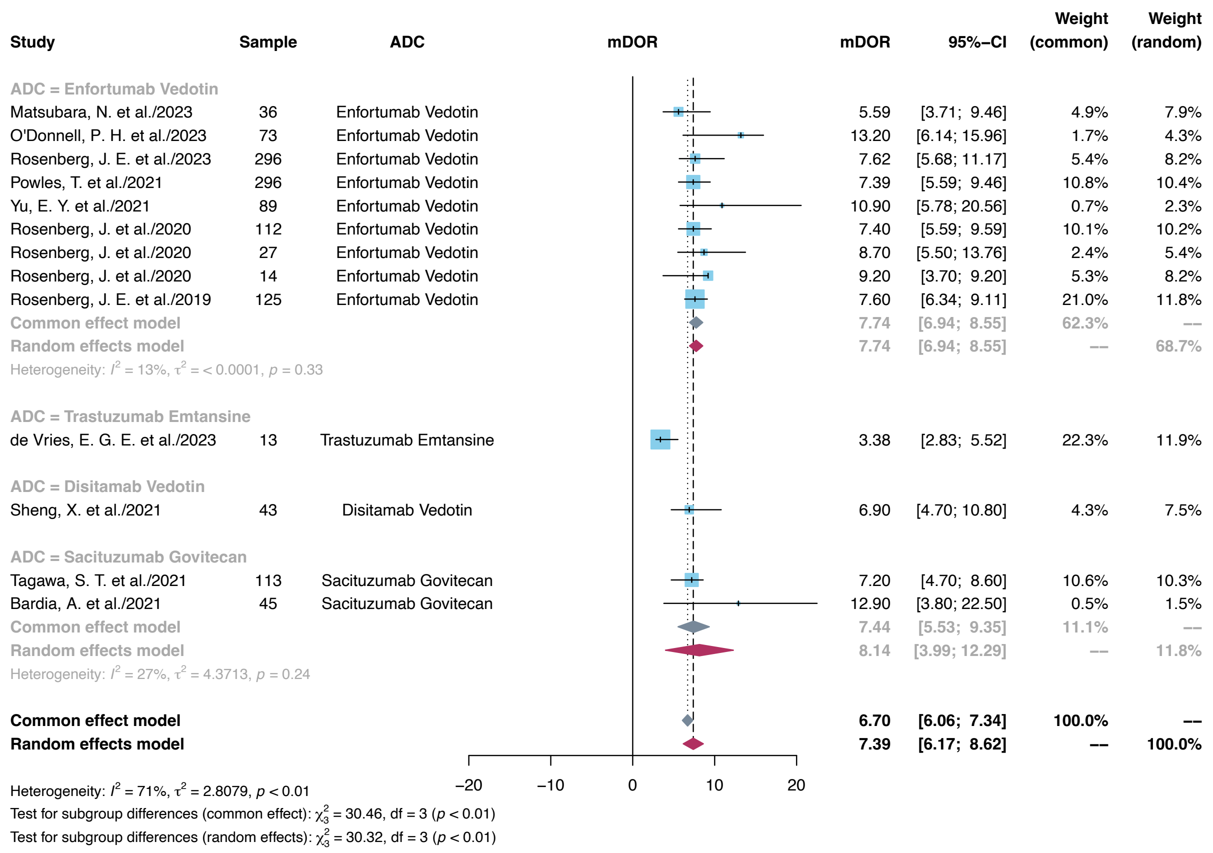

Figure S49 Forest plot of subgroup analysis of median DOR based on ADC classes.

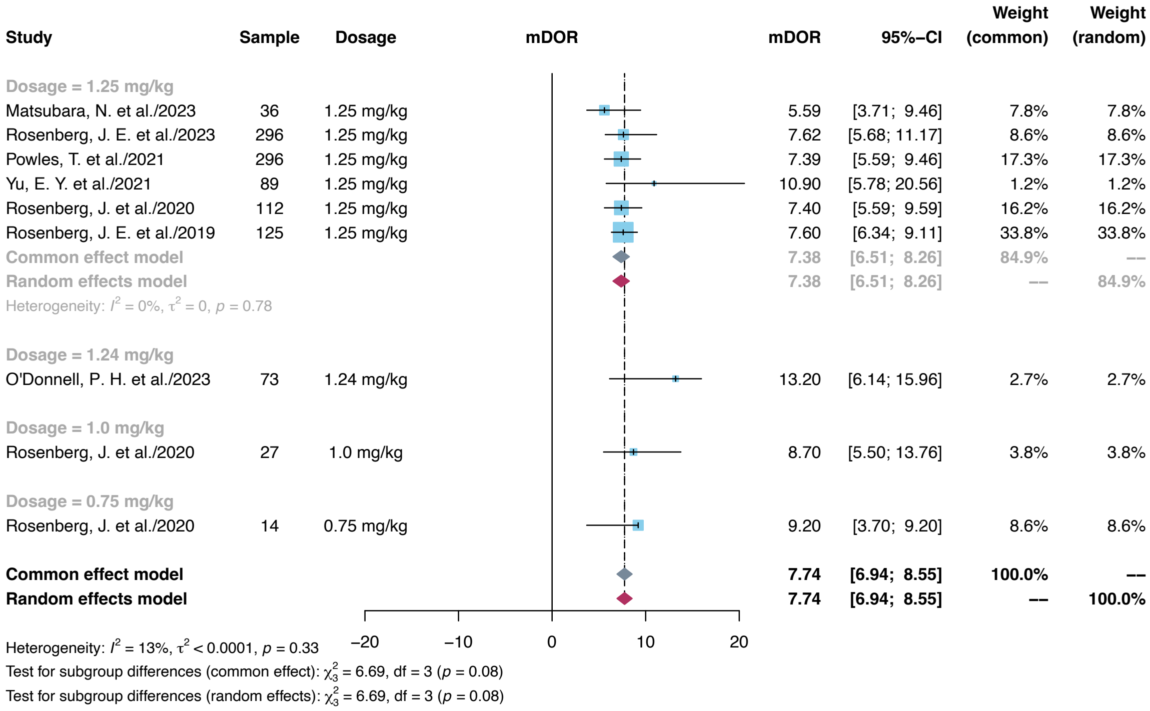

Figure S50 Forest plot of dosage analysis of median DOR in EV cohorts.

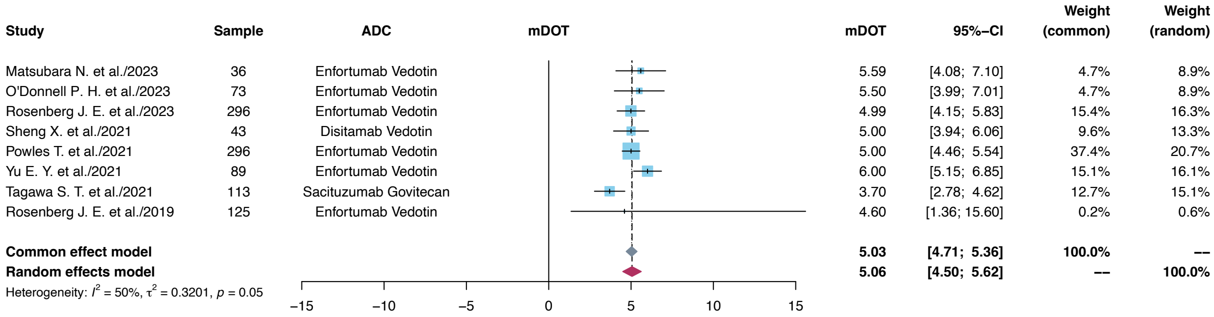

Figure S51 Forest plot of median DOT.

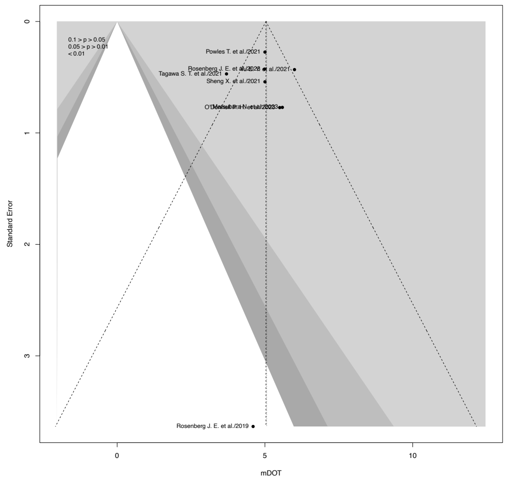

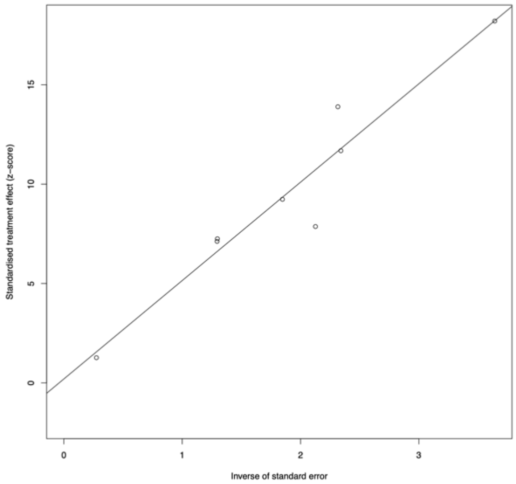

Figure S52A Funnel plot of median DOT.

Figure S52B Egger test of median DOT.

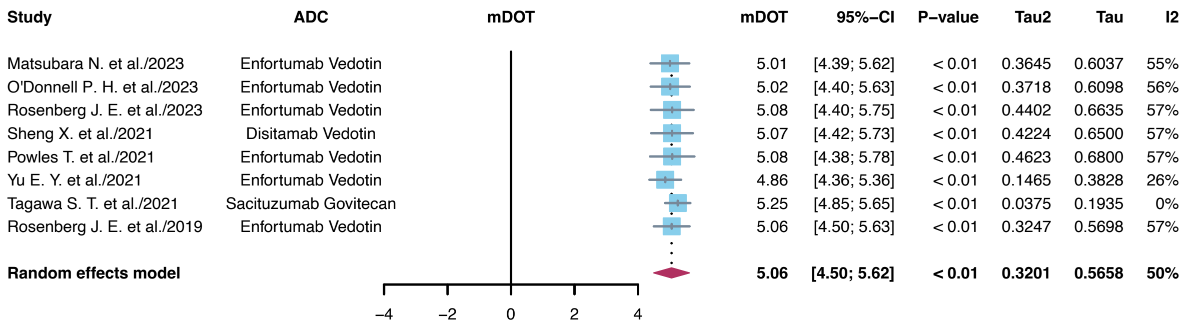

Figure S53 Sensitivity analysis of median DOT.

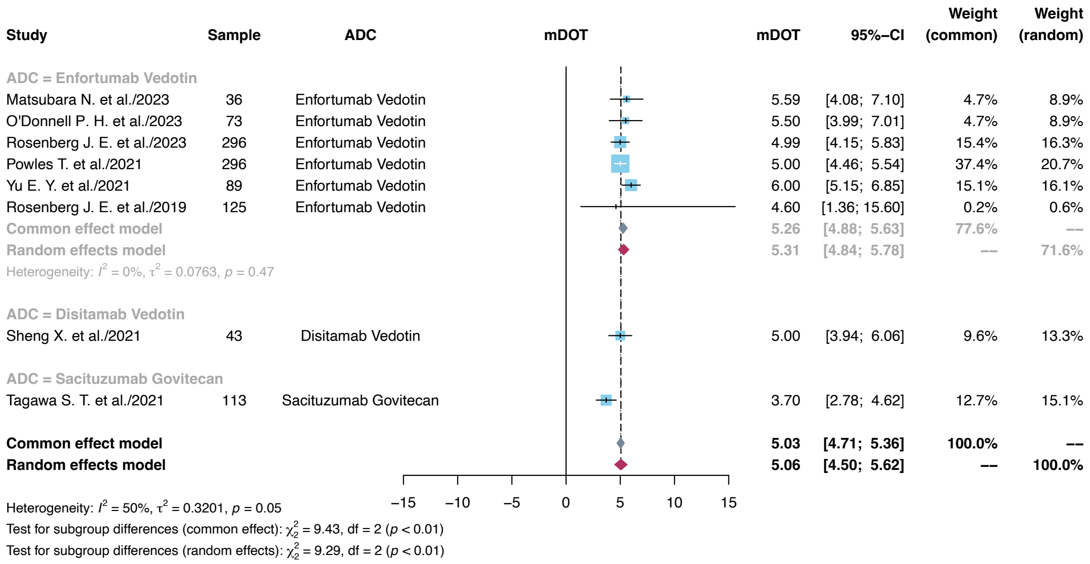

Figure S54 Forest plot of subgroup analysis of median DOT based on ADC classes.

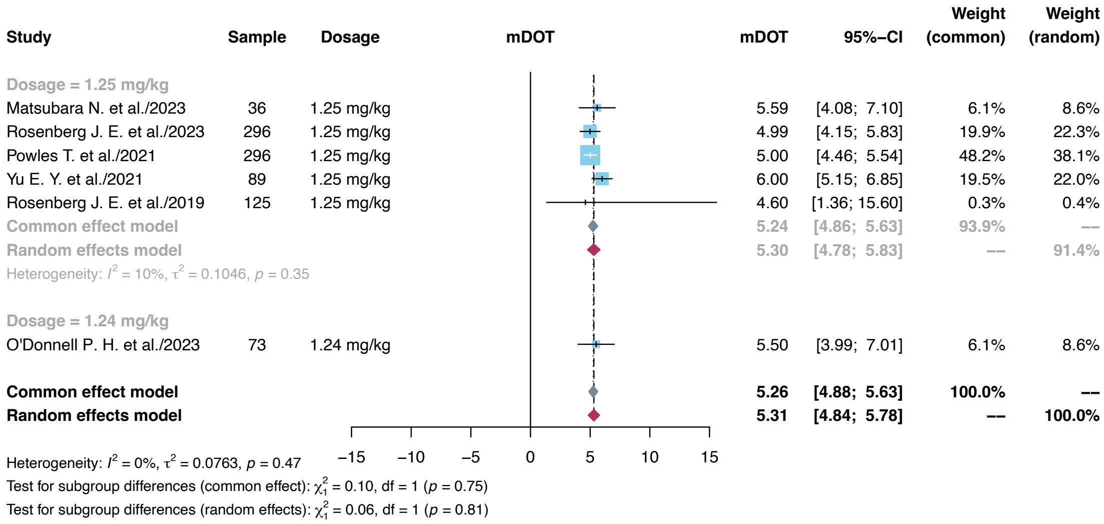

Figure S55 Forest plot of dosage analysis of median DOT in EV cohorts.

AE

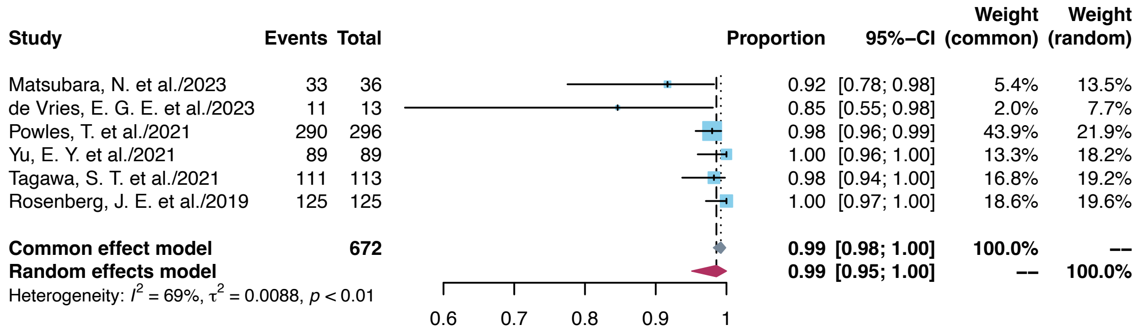

Figure S56 Forest plot of AEs rate.

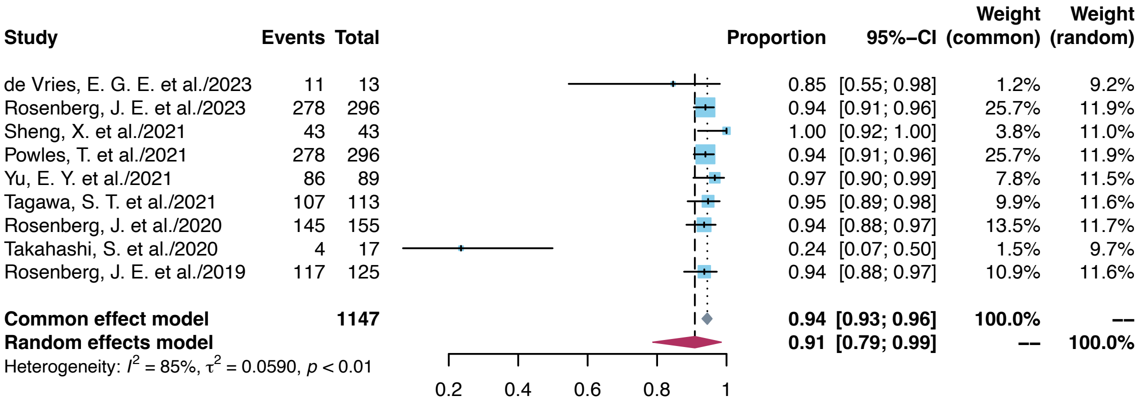

Figure S57 Forest plot of TRAEs rate (any grade).

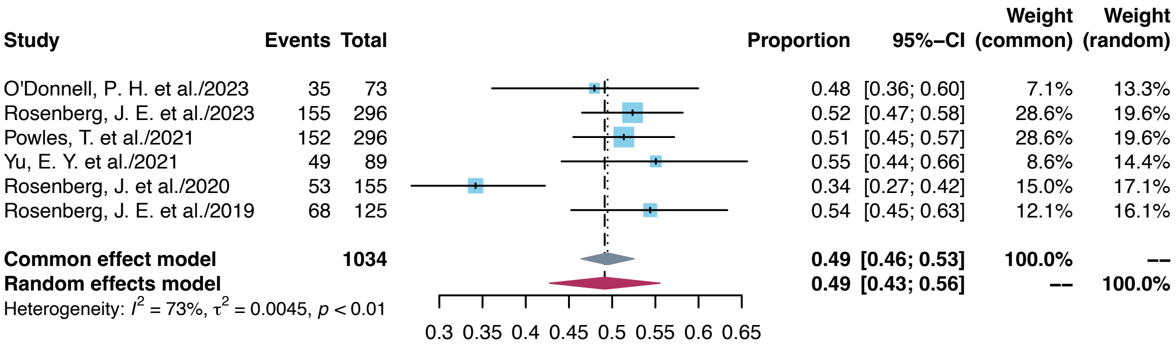

Figure S58 Forest plot of TRAEs rate (grade≥3).

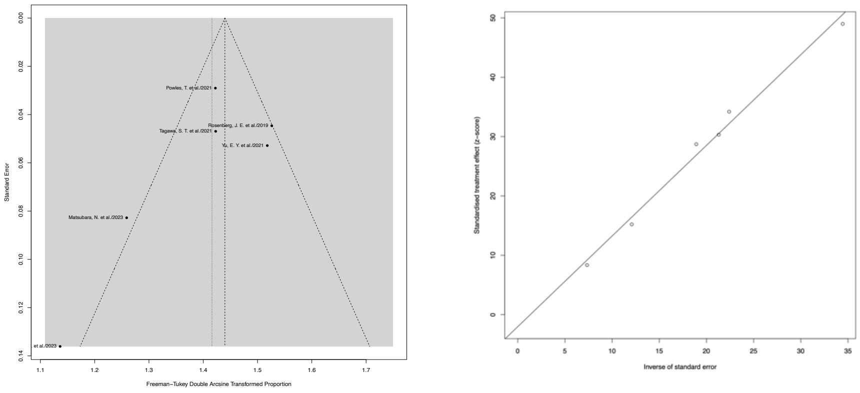

(A) AEs.

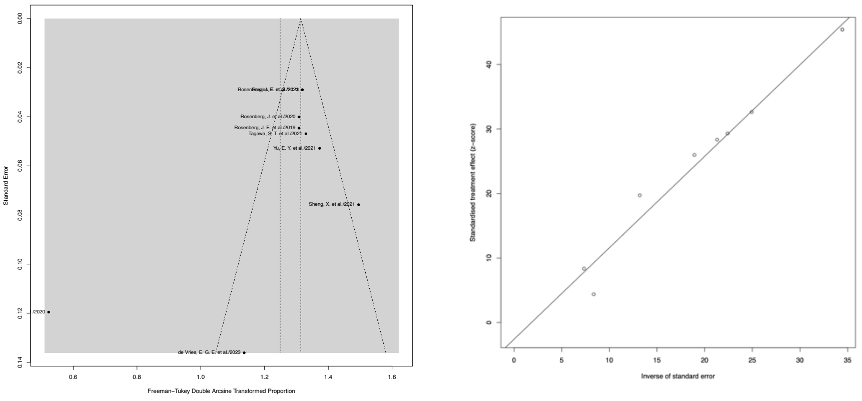

(B) Any grade TRAEs.

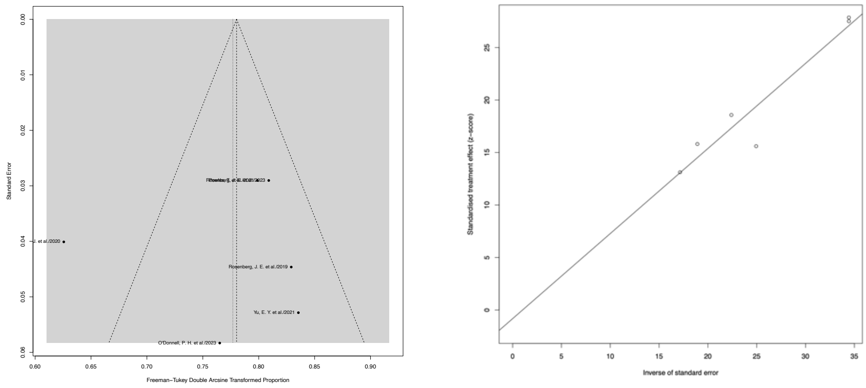

(C) Grade≥3 TRAEs.

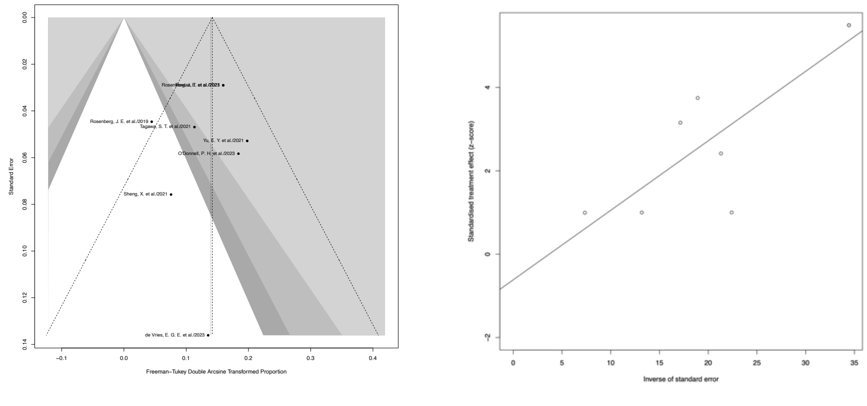

(D) TRAEs leading to death.

Figure S59 Funnel plot and Egger test of the prevalence rate of AEs, any grade TRAEs, grade≥3 TRAEs, and TRAEs leading to death. There is no evidence of publication bias.

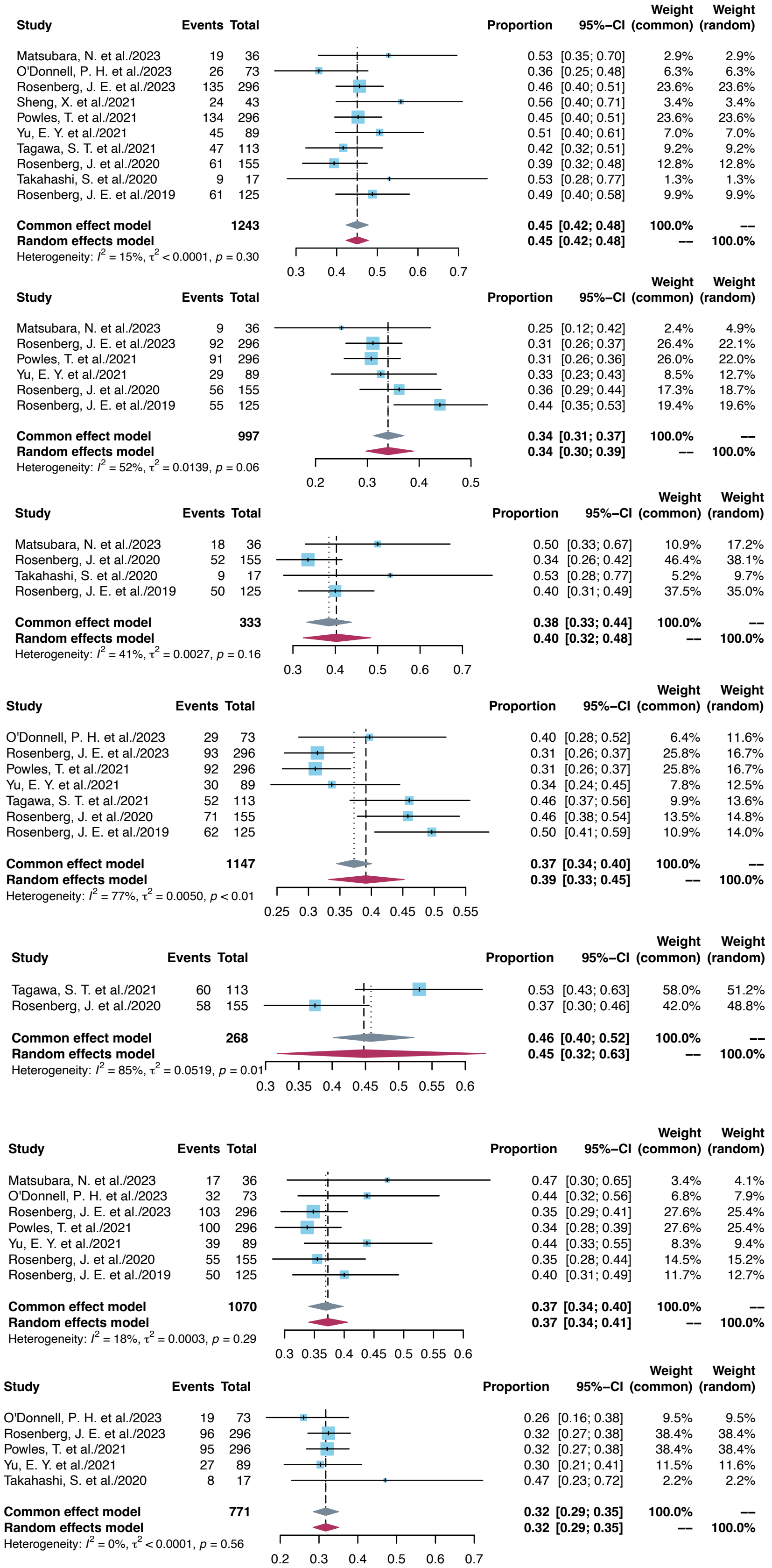

(A) Rate of alopecia.

(B) Rate of decreased appetite.

(C) Rate of dysgeusia.

(D) Rate of fatigue.

(E) Rate of nausea.

(F) Rate of peripheral sensory neuropathy.

(G) Rate of pruritus.

Figure S60A Forest plot of prevalence rate of seven most common TRAEs (any grade).

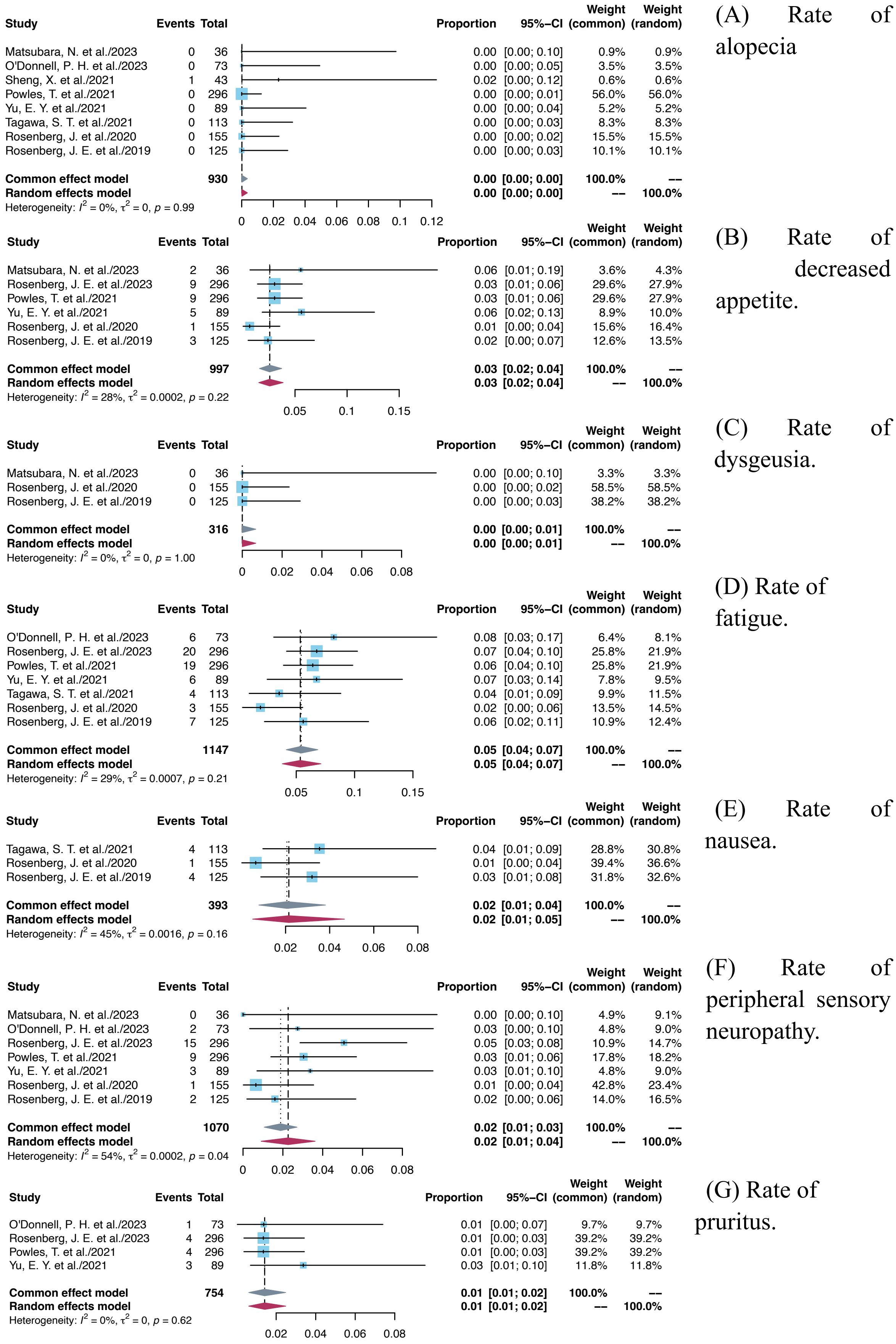

Figure S60B Forest plot of prevalence rate of seven most common TRAEs (grade≥3).

Table S1 Search Strategy for clinical trials of ADCs treated patients with UC.

Term	Population		Intervention		Study
	MeSH	Free words	MeSH	Free words	
	transitional cell carcinoma	cancer of the urothelium' OR 'carcinoma of the urothelium' OR 'carcinoma, transitional cell' OR 'metastatic transitional cell' OR 'metastatic urothelial' OR 'papillary TCC' OR 'papillary transitional cell carcinoma' OR 'papillary urothelial carcinoma' OR 'transitional cell cancer' OR 'transitional cell carcinomata' OR 'transitional cell carcinomatosis' OR 'upper tract urothelial carcinoma' OR 'urothelial cancer' OR 'urothelial carcinogenesis' OR 'urothelial carcinoma' OR 'urothelial cell cancer' OR 'urothelial cell carcinogenesis' OR 'urothelial cell carcinoma' OR 'urothelial malignancies' OR 'urothelial malignancy' OR 'urothelial metastases' OR 'urothelial metastasis' OR 'urothelium cancer' OR 'urothelium carcinogenesis' OR 'urothelium carcinoma'	antibody drug conjugate	'antibody-drug conjugate' OR 'drug-antibody conjugate' OR 'antibody drug conjugate' OR 'Enfortumab Vedotin' OR ' Sacituzumab Govitecan' OR 'Trastuzumab Deruxtecan' OR ' Disitamab Vedotin' OR 'ASG-15ME'	(clinical[tiab] AND trial[tiab]) OR "clinical trials as topic"[mesh] OR "clinical trial"[pt] OR random*[tiab] OR "random allocation"[mesh] OR "therapeutic use"[sh]

*The search terms for study designs are based on the filters announce by the Harvard Library.

Table S2 Overview of information.

Study	Trial number	Phase	RCT	Region	Sample	Mean	Median (range), y	Sex (M/F)	Cancer	Dosage, mg/Kg	Target	ADC	Endpoints	mFU, mo	PFS			OS			mDOR (95%CI), mo	mDOT (range), mo	ORR (95%CI), mo	CBR,%	DCR, 95%CI	CR	PR	SD	Death	AEs			TRAEs				Bellmunt risk score		Check	
															mPFS (95%CI), mo	PFS, 6m,%	PFS, 12m,%	mOS (95%CI), mo	OS, 6m,%	OS, 12m,%										n	sAEs	Grade ≥ 3	n	Grade ≥ 3	Serious	Death	0-1	≥2	IA	BICR
Matsubara, N. et al. /2023	NCT03474107	III	Y	Asia	36	69.86 (	70 (58, 81)	28/8	la/mUC	1.25	Nectin-4	EV	PFS, OS, DOR, DOT, ORR, DCR, AEs	10.71	6.47 (5.39, 12.94)	54	32	15.18 (11.56, -)	94.3	67.2	5.59 (3.71, 9.46)	5.59 (0.9, 19.4)	34.4 (18.57, 53.19)	NR	84.4 (67.21, 94.72)	2	9	16	10	33	NR	23	NR	NR	NR	NR	33	3	Y	N
de Vries, E. G. E. et al. /2023	NCT02999672	II	N	Europe	13	NR	62 (32, 78)	12/1	aUBC		HER2	TE	PFS, OS, DOR, ORR, AEs	7.39 (4.11, 10.02)	2.20 (1.18, 4.30)	NR	NR	7.03 (3.75, -)	NR	NR	3.38 (2.83, 5.52)	NR	38.5 (16.57, 64.52)	NR	46.15	0	5	1	3	11	6	7	11	NR	NR	0	NR	NR	Y	N
					7	NR	50 (38, 78)	6/1	aUBC	2.4	HER2	TE		NR	NR	NR	NR	NR	NR	NR	3.14 (2.83, 3.91)	NR	57.1 (22.53, 87.12)	NR	71.42	0	4	1	3	7	5	6	7	NR	NR	0	NR	NR	Y	N
					6	NR	66 (32, 69)	6/0	aUBC	3.6	HER2	TE		NR	NR	NR	NR	NR	NR	NR	5.52	NR	16.7 (0.85, 58.18)	NR	16.67	0	1	0	0	4	1	1	4	NR	NR	0	NR	NR	Y	N
O'Donnell, P. H. et al. /2023	NCT03288545	Ib/II	Y	Europe Americas	73	NR	73 (56, 89)	56/17	la/mUC	1.24	Nectin-4	EV	PFS, OS, DOR, DOT, ORR, DCR, AEs	15.0 (12.65, 17.35)	8.0 (6.05, 10.34)	62.4	35.8 (21.86, 49.89)	21.7 (15.20, -)	83.6	70.7 (58.12, 89.9)	13.2 (6.14, 15.97)	5.5 (0.5, 26.9)	45.2 (33.5, 57.2)	NR	79.5 (68.4, 88.1)	3	30	25	26	NR	NR	NR	NR	35	11	2	NR	NR	Y	Y
Rosenberg, J. E. et al. /2023	NCT03474107	III	Y	Europe Asia Americas Oceania	301	NR	68 (34, 85)	238/63	la/mUC	1.25	Nectin-4	EV	PFS, OS, DOR, DOT, ORR, DCR, AEs	23.75	5.55 (5.32, 6.28)	33.3	18.26	12.91 (11.01, 14.92)	78	53	7.62 (5.68, 11.17)	4.99 (0.5, 29.9)	41.32 (35.57, 47.25)	NR	71.88 (66.30, 76.99)	20	99	88	207	NR	NR	NR	278	155	67	7	NR	NR	Y	N
Sheng, X. et al. /2021	NCT03507166	II	N	Asia	43	62.3 (8.18)	64 (45, 75)	33/10	la/mUC	2	HER2	DV	PFS, OS, DOR, DOT, ORR, DCR, AEs	20.3 (19.9, 21.6)	6.9 (5.6, 8.9)	59.10 (42.60, 72.30)	21.9	13.9 (9.1, -)	84	55.8 (39.8, 69.1)	6.9 (4.7, 10.8)	5 (0.5, 14.7)	51.2 (35.5, 66.7)	NR	90.7 (77.9, 97.4)	0	22	17	0	0	NR	NR	43	NR	NR	NR	NR	NR	N	Y
Powles, T. et al. /2021	NCT03474107	III	Y	Europe Asia Americas Oceania	301	NR	68 (34, 85)	238/63	la/mUC	1.25	Nectin-4	EV	PFS, OS, DOR, DOT, ORR, DCR, AEs	11.1	5.55 (5.32, 5.82)	44	21.7	12.88 (10.58, 15.21)	78	51.5 (44.6, 58.0)	7.39 (5.59, 9.46)	5.0 (0.5, 19.4)	40.6 (34.90, 46.54)	NR	71.9 (66.30, 76.99)	14	103	90	134	290	138	NR	278	152	NR	7	201	90	Y	N
Yu, E. Y. et al. /2021	NCT03219333	II	N	Americas Asia Europe	89	NR	75 (68, 78)	66/23	la/mUC	1.25	Nectin-4	EV	PFS, OS, DOR, DOT, ORR, DCR, AEs	13.4 (11.3, 18.9)	5.8 (5.03, 8.28)	50 (38.60, 60.40)	33 (21.9, 43.6)	14.7 (10.51, 18.20)	85.3	59.4	10.9 (5.78, -)	6 (2.8, 8.3)	52 (41, 62)	NR	58.4 (47.5, 68.8)	18	28	27	8	89	35	NR	86	49	15	3	NR	NR	Y	Y
Tagawa, S. T. et al. /2021	NCT03547973	II	N	Americas	113	NR	66 (33, 90)	88/25	la/mUC	10	TROP2	SG	PFS, OS, DOR, DOT, ORR, CBR, AEs	9.1 (0, 19.9)	5.4 (3.5, 7.2)	44.1	12.5	10.9 (9.0, 13.8)	66.6	45	7.2 (4.7, 8.6)	3.7 (0, 20)	27.4 (19.5, 36.6)	37.2 (28.3, 46.8)	61.06	6	25	38	3	111	NR	NR	107	NR	NR	1	72	41	Y	Y

Study	Trial number	Phase	RCT	Region	Sample	Mean	Median (range), y	Sex (M/F)	Cancer	Dosage, mg/Kg	Target	ADC	Endpoints	mFU, mo	PFS			OS			mDOR (95%CI), mo	mDOT (range), mo	ORR (95%CI), mo	CBR,%	DCR, 95%CI	CR	PR	SD	Death	AEs			TRAEs				Bellmunt risk score		Check	
															mPFS (95%CI), mo	PFS, 6m,%	PFS, 12m,%	mOS (95%CI), mo	OS, 6m,%	OS, 12m,%										n	sAEs	Grade ≥ 3	n	Grade ≥ 3	Serious	Leading to death	0-1	≥2	IA	BICR
Bardia, A. et al. /2021	NCT01631552	I/II	N	Americas	45	NR	NR	NR	aUC	10	TROP2	SG	PFS, OS, DOR, ORR, CBR, AEs	NR	6.8 (3.6, 9.7)	NR	NR	16.8 (9.0, 21.9)	NR	NR	12.9 (3.8, 22.5)	NR	28.9 (16.4, 44.3)	44.4 (29.6, 60.0)	64.44	2	11	16	NR	NR	NR	NR	NR	NR	NR	NR	NR	NR	Y	N
Rosenberg, J. et al. /2020	NCT02091999	I	N	Americas	155	NR	67 (24, 86)	111/44	mUC		Nectin-4	EV	PFS, OS, DOR, ORR, DCR, AEs	NR	NR	NR	NR	NR	NR	NR	NR	NR	36.77	NR	70.32	8	49	52	4	NR	NR	NR	145	53	NR	NR	NR	NR	Y	N
					112	NR	NR	NR	mUC	1.25	Nectin-4	EV		16.4	5.4 (5.06, 6.28)	42.3	19.3	12.3 (9.33, 15.31)	74	51.8	7.4 (5.59, 9.59)	NR	42.9 (33.6, 52.6)	NR	71.4 (62.1, 79.6)	5	43	32	NR	NR	NR	NR	NR	NR	NR	NR	NR	NR	Y	N
					27	NR	NR	NR	mUC	1	Nectin-4	EV		NR	NR	NR	NR	NR	NR	NR	8.7 (5.5, -)	NR	18.5 (6.3, 38.1)	NR	63 (42.4, 80.6)	3	2	12	NR	NR	NR	NR	NR	NR	NR	NR	NR	NR	Y	N
					14	NR	NR	NR	mUC	0.75	Nectin-4	EV		NR	NR	NR	NR	NR	NR	NR	9.2 (3.7, 9.2)	NR	21.4 (4.7, 50.8)	NR	71.4 (41.9, 91.6)	0	3	7	NR	NR	NR	NR	NR	NR	NR	NR	NR	NR	Y	N
					2	NR	NR	NR	mUC	0.5	Nectin-4	EV		NR	NR	NR	NR	NR	NR	NR	9.3	NR	50 (1.26, 98.7)	NR	100 (15.8, 100.0)	0	1	1	NR	NR	NR	NR	NR	NR	NR	NR	NR	NR	Y	N
					23	NR	NR	NR	mUC	1.25	Nectin-4	EV		NR	NR	NR	NR	NR	NR	NR	NR	NR	43.48	NR	60.87	2	8	4	NR	NR	NR	NR	NR	NR	NR	NR	NR	NR	Y	N
					89	NR	NR	NR	mUC	1.25	Nectin-4	EV		NR	NR	NR	NR	12.3 (9.33, 16.10)	76	51	7.3 (4.2, 9.6)	NR	43 (32.3, 53.6)	NR	74 (63.8, 82.9)	3	35	28	NR	NR	NR	NR	NR	NR	NR	NR	NR	NR	Y	N
					74	NR	NR	NR	mUC	1.25	Nectin-4	EV		NR	6.6 (5.32, 8.15)	53.3	24.4	NR	NR	NR	7.5 (5.8, -)	NR	45 (33.0, 56.6)	NR	81 (70.3, 89.3)	8	25	27	NR	NR	NR	NR	NR	NR	NR	NR	NR	NR	N	Y
Takahashi, S. et al. /2020	NCT03070990	I	Y	Asia	17	NR	67 (57, 82)	15/2	la/mUC		Nectin-4	EV	PFS, ORR, DCR, AEs	NR	8.1 (3.5, -)	NR	NR	NR	NR	NR	NR	NR	35.3	NR	76.5	1	5	7	NR	NR	NR	10	4	NR	NR	NR	NR	NR	Y	N
					9	NR	67 (61, 82)	8/1	la/mUC	1	Nectin-4	EV		NR	NR	NR	NR	NR	NR	NR	NR	NR	44.4	NR	100	1	3	5	NR	NR	NR	5	2	NR	NR	NR	NR	NR	Y	N
					8	NR	67.5 (57, 78)	7/1	la/mUC	1.25	Nectin-4	EV		NR	NR	NR	NR	NR	NR	NR	NR	NR	25	NR	50	0	2	2	NR	NR	NR	5	2	NR	NR	NR	NR	NR	Y	N
Rosenberg, J. E. et al. /2019	NCT03219333	II	N	Americas Asia	125	NR	69 (40, 84)	88/37	la/mUC	1.25	Nectin-4	EV	PFS, OS, DOR, DOT, ORR, DCR, AEs	10.2 (0.5, 16.5)	5.8 (4.9, 7.5)	49.5	18.35	11.7 (9.1, -)	79.7	49.8	7.6 (6.34, 9.11)	4.6 (-, 15.6)	44 (35.1, 53.2)	NR	50 (41.3, 59.5)	15	40	35	7	125	59	NR	117	68	24	0	72	52	Y	Y

*The same study may contain multiple cohorts, with information from different cohorts presented separately. The results extracted using the data extraction tool only show the ratio without 95% CI. Most studies rely on investigator review. In cases where a study report presents both IA and BICR, the latter is chosen for inclusion in this table. CI, confidence interval; mUC, metastatic urothelial carcinoma; laUC, locally advanced urothelial carcinoma; aUBC, advanced urothelial bladder cancer; HER2, human epidermal growth factor receptor 2; Nectin-4, nectin cell adhesion molecule 4; TROP2, trophoblast cell-surface antigen 2; EV, Enfortumab Vedotin; TE, Trastuzumab Emtansine; DV, Disitamab Vedotin; SG, Sacituzumab Govitecan; BICR, blinded independent central review; IA, investigator review; RCT, randomized control trial; ORR, objective response rate; DCR, disease control rate; mPFS, median progression-free survival; mOS, median overall survival; mDOR, median duration of response; mDOT, median duration of treatment; CR, complete response; PR, partial response; SD, stable disease; CBR, clinical benefit rate; BOR, best overall response; mFU, median follow-up; sAE, serious adverse event; TRAE, treatment-related adverse event; Y, yes; N, no; NR, not report.

Figure A1 Risk of bias graph and risk of bias summary in RCT group.

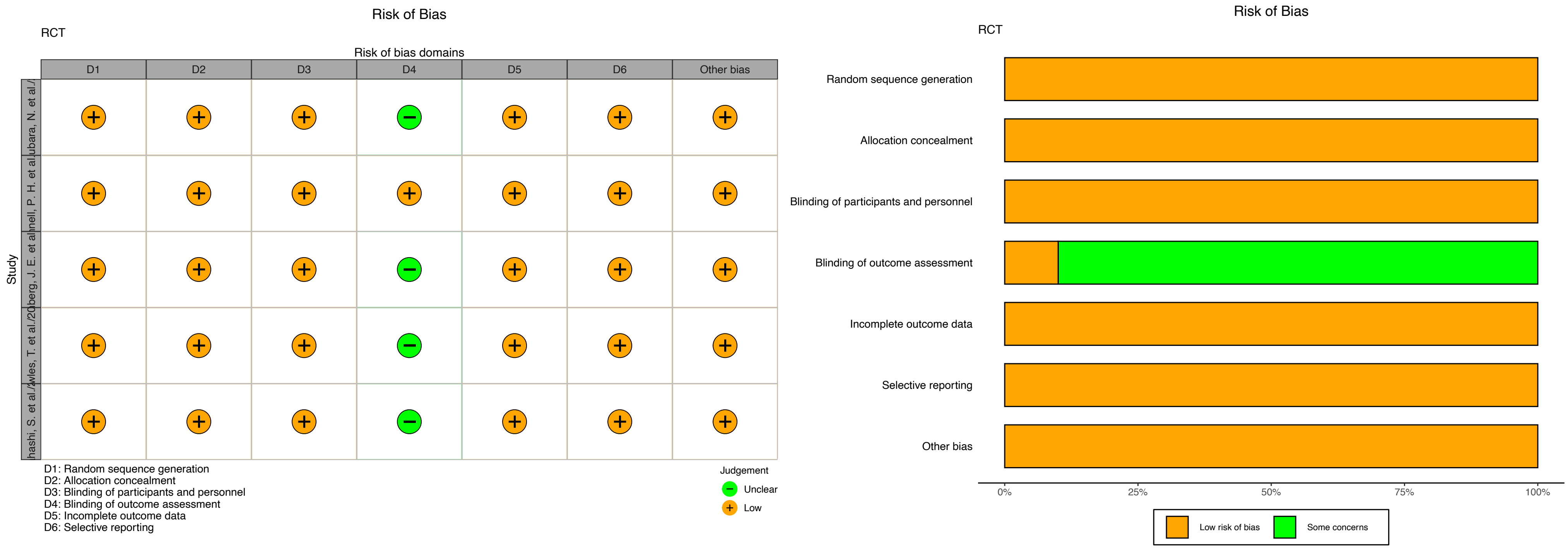

Table A1 Quality assessment of the single-arms in the meta-analysis by Newcastle–Ottawa Scale (NOS).

Study	Sample	Q1	Q2	Q3	Q4	Q5	Q6	Q7	Q8	Total
de Vries, E. G. E. et al. /2023	13	1	1	1	1	0	0	0	1	5
Sheng, X. et al. /2021	43	1	1	1	1	1	1	0	1	7
Yu, E. Y. et al. /2021	89	1	1	1	1	1	1	0	1	7
Tagawa, S. T. et al. /2021	113	1	1	1	1	0	0	0	1	5
Bardia, A. et al. /2021	45	1	1	1	1	0	0	0	0	4
Rosenberg, J. et al. /2020	155	1	1	1	1	1	0	0	0	5
Rosenberg, J. E. et al. /2019	125	1	1	1	1	1	1	0	1	7

* Numbers Q1-8 in heading signified as: Q1, Representativeness of the exposed cohort; Q2, Selection of the non-exposed cohort; Q3, Ascertainment of exposure; Q4, Demonstration that outcome of interest was not present at start of study; Q5, Comparability of cohorts on the basis of the design or analysis; Q6, Assessment of outcome; Q7, Follow-up was long enough for outcomes to occur; Q8, Adequacy of follow up of cohorts.

Method S1 R code for ORR subgroup analysis.

```
library(meta)
a <- read.csv('ORR(total).csv', header = TRUE)
n=a$Sample
events=a$Event

rate <- transform(a, p = events/n, log = log(events/n), logit = log((events/n)/(1 - events/n)), arcsin.size = asin(sqrt(events/(n + 1))), darcsin = 0.5 * (asin(sqrt(events/(n + 1)) )) + asin((sqrt(events + 1)/(n + 1))))
shapiro.test(rate$p) # PRAW
shapiro.test(rate$log) # PLN
shapiro.test(rate$logit) # PLOGIT
shapiro.test(rate$arcsin.size) # PAS
shapiro.test(rate$darcsin) # PFT

meta1 <- metaprop(events, n, data = a, studlab = paste(Author), sm = "PRAW", comb.random = T)
pdf(file="ORR(total).pdf",width =10, height = 7)
forest(meta1, family = 'sans', fontsize = 12, lwd = 2,
        col.diamond.common = 'lightslategrey',
        col.diamond.lines.common = 'lightslategrey',
        col.diamond.random = 'maroon',
        col.diamond.lines.random = 'maroon',
        col.square = 'skyblue',
        col.study = 'black',
        lty.common = 3,lty.random = 5, plotwidth = '7cm',
        colgap.forest.left = '0.5cm', colgap.forest.right = '0cm')
dev.off()

pdf(file="ORR(total)_funnel.pdf",width =12, height = 12)
funnel(meta1, common = T, pch =16,
        contour.levels = c(0.9, 0.95, 0.99),studlab = TRUE,
        col.contour = c("darkgray", "gray", "lightgray"))
legend(-2.00, 0.10, c("0.1 > p > 0.05", "0.05 > p > 0.01", "< 0.01"),bty="n",cex = 0.8)
dev.off()

pdf(file = "ORR(total)_Asymmetry.pdf", width = 10, height = 10)
# Begg
metabias(meta1, k.min = 10, method.bias = "Begg", correct = TRUE, plotit = TRUE)
# Egger
metabias(meta1, k.min = 10, method.bias = "Egger", plotit = TRUE)
dev.off()
```

```
pdf(file="ORR(total)_tf.pdf",width =12, height = 12)
tf <- trimfill(meta1,comb.random = TRUE)
summary(tf)
funnel(tf,studlab = T)
dev.off()

pdf(file="ORR(total)_subgroup1.pdf",width = 10,height = 10)
meta3 <- metaprop(events, n, data = a, studlab = paste(Author), sm = "PLOGIT",
                  comb.random = T, byvar = ADC)
forest(meta3, leftcols = c("Author","Sample", "ADC"),
digits=2,family="sans",fontsize=10,lwd=2,col.diamond.common="lightslategray",col.
diamond.lines.common="lightslategray",

col.diamond.random="maroon",col.diamond.lines.random="maroon",col.square="sky
blue",col.study="black",
       lty.common = 3,lty.random =
5,plotwidth="8cm",colgap.forest.left="1cm",colgap.forest.right="0.5cm",just.forest="
center",colgap.left="0.5cm",
       colgap.right="0.5cm")
dev.off()

pdf(file="ORR(total)_subgroup2.pdf",width = 10,height = 10)
meta3 <- metaprop(events, n, data = a, studlab = paste(Author), sm = "PLOGIT",
                  comb.random = T, byvar = NCT)
forest(meta3, leftcols = c("Author","Sample", "NCT"),
digits=2,family="sans",fontsize=10,lwd=2,col.diamond.common="lightslategray",col.
diamond.lines.common="lightslategray",

col.diamond.random="maroon",col.diamond.lines.random="maroon",col.square="sky
blue",col.study="black",
       lty.common = 3,lty.random =
5,plotwidth="8cm",colgap.forest.left="1cm",colgap.forest.right="0.5cm",just.forest="
center",colgap.left="0.5cm",
       colgap.right="0.5cm")
dev.off()
```

Glossary

UC	Urothelial carcinoma
BC	Bladder cancer
EV	Enfortumab Vedotin
TE	Trastuzumab Emtansine
DV	Disitamab Vedotin
SG	Sacituzumab Govitecan
BICR	Blinded independent central review
IA	Investigator review
RCT	Randomized control trial
NRSI	Non-randomized studies of interventions
RECIST	Response Evaluation Criteria in Solid Tumors
CTCAE	Common Terminology Criteria for Adverse Events
ROB	Risk of bias
NOS	Newcastle–Ottawa Scale
AE	Adverse event
TRAE	Treatment-related adverse events
ORR	Objective response rate
DCR	Disease control rate
PFS	Progression-free survival
OS	Overall survival
DOR	Duration of response
DOT	Duration of treatment
CR.	Complete response
PR	Partial response
SD	Stable disease
CBR	Clinical benefit rate
BOR	Best overall response
UP	Follow-up
HER2	human epidermal growth factor receptor 2
TROP2	trophoblast cell-surface antigen 2
Nectin-4	cell adhesion molecule 4 Nectin-4
